# Comparative Effectiveness of Non-Pharmacological Interventions for Reducing Heart Failure-Related Unplanned Readmissions: A Systematic Review and Network Meta-Analysis

**DOI:** 10.3390/jcm15145344

**Published:** 2026-07-08

**Authors:** Fangjia Shen, Yuxi Chen, Ziqian Huang

**Affiliations:** School of Nursing, Sun Yat-Sen University, Guangzhou 510000, China; shenfj@mail2.sysu.edu.cn (F.S.); chenyx856@mail2.sysu.edu.cn (Y.C.)

**Keywords:** non-pharmacological interventions, readmissions, heart failure, systematic review, network meta-analysis

## Abstract

**Background:** Heart failure (HF) is a major global health challenge associated with high morbidity, mortality, and frequent unplanned readmissions. These readmissions impose substantial physical, psychological, socioeconomic, and healthcare-system burdens. Although non-pharmacological interventions (NPIs) have shown promise in HF management, no network meta-analysis (NMA) has comprehensively compared their effects on HF-related unplanned readmissions. **Methods:** A computerized search was conducted on databases, including PubMed, Web of Science, Embase, the Cochrane Library, PsycINFO, and CINAHL for randomized controlled trials (RCTs) from inception to 15 March 2026. Study selection, data extraction, and risk-of-bias assessment were performed independently by two reviewers. A frequentist NMA was conducted to synthesize direct and indirect evidence. The outcome was HF-related unplanned readmissions. **Results:** A total of 39 studies with 12 NPIs and 7333 patients were included. The NMA suggested that several NPIs were associated with reduced HF-related unplanned readmissions compared with usual care. Exercise therapy combined with multidisciplinary team management showed the highest ranking probability, with an RR of 0.32 (95% CI: 0.15 to 0.65), followed by patient education combined with health information tracking (RR: 0.42, 95% CI: 0.25 to 0.72), patient education alone (RR: 0.56, 95% CI: 0.46 to 0.69), and patient education combined with multidisciplinary team management (RR: 0.57, 95% CI: 0.43 to 0.78). **Conclusions:** Our NMA suggests that several NPIs may reduce HF-related unplanned readmissions, with exercise therapy combined with multidisciplinary team management showing the highest ranking probability and patient education combined with health information tracking also ranking favorably. Given the sparse head-to-head comparisons, heterogeneity across studies, and low certainty of evidence in some comparisons, these ranking-based findings should be cautiously interpreted as exploratory. Further multicenter RCTs are needed to confirm these findings and evaluate long-term effectiveness and implementation outcomes.

## 1. Introduction

Heart failure (HF) represents a major global public health challenge, affecting more than 64.3 million people worldwide and accounting for over 2 million new cases annually [1]. The burden is particularly substantial in China, where the number of patients with heart failure is projected to increase from 13.1 million to 22.7 million by 2035 [2]. HF is associated with high morbidity, high mortality, and frequent hospital readmissions [3]. Approximately 20% to 50% of hospitalized HF patients are readmitted within 30 days to one year after discharge [4,5]. A substantial proportion of these readmissions is attributable to worsening HF symptoms or signs and are commonly defined as HF-related readmissions [6,7]. These events are associated with increased frailty, higher mortality risk, poorer quality of life, and substantial psychological distress, including anxiety and depression [8,9,10]. They also impose considerable economic pressure on patients and healthcare systems through direct medical costs, productivity losses, and increased healthcare utilization [11,12]. Therefore, reducing unplanned HF-related readmissions has become an important priority in HF management.

Pharmacological therapy is central to the management of HF. However, adverse drug effects, such as renal dysfunction and electrolyte disturbances, may affect treatment adherence and limit dose optimization or long-term implementation in some patients [13,14]. Consequently, increasing attention has been given to non-pharmacological interventions (NPIs) as complementary strategies for HF care. NPIs generally refer to structured care approaches that do not involve pharmaceutical agents or invasive procedures, including exercise training, psychological therapy, patient education, symptom and health status monitoring, and multidisciplinary team management [15,16,17]. These interventions may improve clinical outcomes through different mechanisms. For example, exercise training can enhance cardiorespiratory fitness and functional capacity, whereas patient education and self-management support can improve symptom recognition, medication adherence, and prompt care-seeking behavior when symptoms worsen [15,16]. Accordingly, NPIs may play an important role in reducing HF-related readmissions.

Previous systematic reviews and meta-analyses have suggested that NPIs are beneficial for patients with HF [18,19]. However, several limitations remain. First, many previous reviews focused mainly on patients with HF with reduced ejection fraction (HFrEF), while patients with preserved (HFpEF) or mildly reduced ejection fraction (HFmrEF) were less frequently considered [18]. Second, most analyses evaluated a limited range of interventions, such as exercise training or patient education compared with usual care (UC), and therefore could not determine the relative effectiveness of multiple NPIs [18,19]. Third, some reviews focused on surrogate outcomes, such as exercise capacity, rather than patient-centered outcomes such as unplanned readmissions [19]. As a result, the comparative effectiveness of different NPIs for reducing unplanned HF-related readmissions remains unclear.

Network meta-analysis (NMA) can address this evidence gap by combining direct and indirect evidence within a single analytical framework. This method is particularly useful when multiple interventions are available, but direct head-to-head comparisons are limited. It also allows the relative effectiveness of different interventions to be estimated and ranked according to their probability of being the most effective strategy [20,21]. Therefore, we conducted an NMA to compare the effects of different NPIs on reducing unplanned HF-related readmissions in patients with HF. Specifically, we aimed to identify and rank the most effective NPIs to inform clinical decision-making and support the optimization of HF management.

## 2. Materials and Methods

This study was registered with PROSPERO (CRD420261308772) and reported in accordance with the Preferred Reporting Items for Systematic Reviews and Meta-Analyses (PRISMA 2020) statement [22]. The PRISMA checklist is provided in Supplementary Table S1.

### 2.1. Data Sources and Search Strategy

A comprehensive literature search was conducted in six electronic databases, namely PubMed, Web of Science, Embase, the Cochrane Library, PsycINFO, and CINAHL, from database inception to 15 March 2026. The search strategy was developed by two reviewers (FJS and ZQH) using Medical Subject Headings (MeSH), together with free-text terms. These terms were combined using the Boolean operators “AND”, “OR”, and “NOT”. Terms related to HF were combined with terms related to NPIs, while records concerning surgical, invasive, device-based, or pharmacological treatments, as well as animal studies, were excluded. According to the predefined eligibility criteria, only RCTs were considered at the initial search stage. The full search strategies for all databases are presented in Supplementary Table S2. To further ensure the comprehensiveness of the search, the reference lists of all included studies and relevant published systematic reviews and meta-analyses were manually screened. In addition, the authors of eligible trials were contacted to obtain missing or additional information when necessary. The literature search and screening were independently conducted by the same two reviewers (FJS and ZQH). Any disagreements were resolved through discussion or, when necessary, consultation with a third reviewer (YXC).

### 2.2. Inclusion and Exclusion Criteria

This review used PICOS screening criteria to identify the included studies and retrieve full texts.

#### 2.2.1. Population

Participants recruited in the studies meeting the following criteria were included: (1) aged 18 years or above; and (2) diagnosed as HF, with no restrictions on HF subtypes (HFrEF, HFpEF, HFmrEF).

#### 2.2.2. Interventions

According to extant meta-analyses [21] and relevant international consensus guidelines [23,24], eligible NPIs comprise five core components:

(1) Exercise therapy (e.g., aerobic workouts, strength training, and high-intensity interval exercises);
(2) Psychological interventions (e.g., cognitive behavioral therapy and motivational interviewing);
(3) Patient education (e.g., structured self-management programs: weight monitoring, and health transition guidance);
(4) Health information tracking (e.g., structured home-based telemonitoring: transmission of symptoms, body weight, vital signs, electrocardiographic information, or other physiological data for remote clinical review, timely decision-making, and early outpatient intervention when deterioration is suspected);
(5) Multidisciplinary team management (e.g., nurse-led clinics with cardiologists, clinical pharmacists, dietitians, and physical therapists).

Interventions involving surgical procedures, invasive techniques, or implantable devices were excluded.

#### 2.2.3. Comparisons

The control group received either NPIs or UC. In this study, UC is defined as the receipt of guideline-recommended medical services and routine follow-up, including routine discharge instructions and outpatient health education.

#### 2.2.4. Outcomes

Rates of readmissions related to HF were the outcome of interest.

#### 2.2.5. Study Design

Only RCTs were eligible for inclusion. Editorials, conference abstracts, reviews, protocols, letters, comments, and studies with unavailable full texts or missing data were excluded.

### 2.3. Data Selection and Extraction

The retrieved records were imported into Zotero 7.0. After removing duplicates, two researchers (FJS and ZQH) independently screened the studies by reviewing the titles and abstracts. Potentially eligible articles were retrieved for full-text assessment and further evaluated against the eligibility criteria. Any disagreements were resolved through discussion between the two researchers or by consulting a third researcher when necessary (YXC). A structured data extraction form was used to collect relevant information from the eligible studies (Supplementary Table S3). The form captured publication data, participant characteristics (sample size, mean age, left ventricular ejection fraction [LVEF], New York Heart Association [NYHA] class, etc.), and intervention details (content, duration, frequency, delivery method, setting, and attrition rates), as well as outcomes, measurement methods, and findings. Data extraction focused primarily on intention-to-treat (ITT) analysis. The first phase of data extraction was carried out by one researcher (FJS), and a second researcher (YXC) verified its accuracy. A third researcher (ZQH) subsequently reviewed all the details and addressed any discrepancies.

### 2.4. Risk of Bias

Risk of bias was assessed using the revised Cochrane risk-of-bias tool for randomized trials (RoB 2) [25], covering five domains: randomization, intervention deviations, missing data, outcome measurement, and selective reporting. Each domain and overall bias were judged as low risk, some concerns, or high risk. Two reviewers (FJS, YXC) independently assessed studies; disagreements were resolved by involving a third reviewer (ZQH). The results were visualized as traffic-light plots using robvis [26].

### 2.5. Statistical Analysis

Pairwise meta-analyses were performed using Review Manager 5.4 (Cochrane Collaboration, Copenhagen, Denmark). Data were pooled when at least three studies evaluated the same intervention category for HF-related unplanned readmissions. Risk ratios (RRs) with 95% confidence intervals (CIs) were calculated for dichotomous outcomes. Statistical heterogeneity was assessed using the *I*^2^ statistic, with values greater than 50% indicating substantial heterogeneity. A random-effect model was used in the presence of substantial heterogeneity; otherwise, a fixed-effect model was applied.

Prespecified subgroup analyses were conducted according to follow-up duration, intervention duration, and disease severity. Follow-up duration was categorized as 3, 6, or 12 months; intervention duration was stratified by whether it exceeded 12 months; and disease severity was defined by the proportion of patients in NYHA class I or II, categorized as <40% or ≥40%. Sensitivity analyses were conducted by sequentially omitting individual studies and by excluding studies at high risk of bias [27]. Publication bias was assessed using funnel plots when at least 10 studies were available [28].

NMA was performed using a frequentist framework with the network package in Stata 17.0 (StataCorp LLC, College Station, TX, USA). A random-effect model was used to synthesize direct and indirect evidence. Network and ranking graphs were created to illustrate the network geometry. To assess global and local inconsistencies, we applied the design-by-treatment interaction model and node-splitting test, respectively [29,30,31]. The NMA was carried out with the assumption of transitivity, facilitating the evaluation of both direct and indirect evidence. We calculated the ranking probability for each non-pharmacological intervention through the surface under the cumulative ranking curve (SUCRA), which ranges from 0% to 100%. A higher SUCRA value suggests a stronger likelihood of the intervention achieving optimal results [32]. Furthermore, when the analysis included 10 or more studies, we employed Egger’s test along with a comparison-adjusted funnel plot to identify potential publication bias [33,34].

### 2.6. Certainty of Evidence

To evaluate the general certainty level of the evidence, this research applied the Grading of Recommendations Assessment, Development, and Evaluation (GRADE) framework. The results were summarized in a table generated with GRADE pro software utilizing Cochrane methods https://www.gradepro.org/ (accessed date 22 May 2026). Two independent researchers evaluated the overall certainty of the evidence, and any disagreements were deliberated to achieve consensus; if needed, a third researcher was consulted. Each comparison’s certainty of evidence was classified as high, moderate, low, or very low based on five criteria: risk of bias, inconsistency, indirectness, imprecision, and publication bias (Supplementary Table S5).

## 3. Results

### 3.1. Search Outcomes

**Figure 1:**
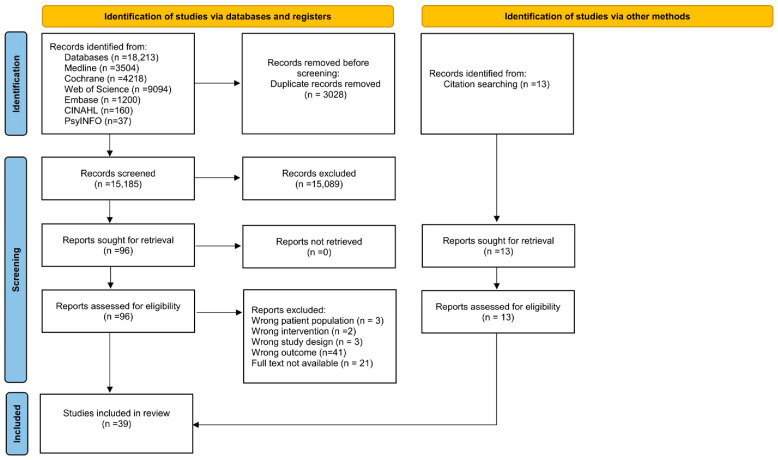
PRISMA flow diagram of study selection.

A total of 18,213 records were identified through database searches. After removal of duplicates, 15,185 records remained for title and abstract screening. Of these, 96 full-text articles identified from database searches were assessed for eligibility, and 70 were excluded for the following reasons: wrong patient population (*n* = 3), wrong intervention (*n* = 2), wrong study design (*n* = 3), wrong outcome (*n* = 41), and full text not available (*n* = 21). Thus, 26 articles from database searches met the inclusion criteria. In addition, 13 eligible articles were identified from the reference lists of included studies. Ultimately, 39 articles were included in the final analysis. The study selection process is presented in the PRISMA flow diagram (Figure 1).

### 3.2. Characteristics of Included Studies

Among the eligible studies, eight studies were conducted in the USA [35,36,37,38,39,40,41,42], six in Italy [43,44,45,46,47,48], four in China [49,50,51,52], three each in Australia [53,54,55] and Japan [56,57,58], and two each in Singapore [59,60], Switzerland [61,62], Germany [63,64], and the UK [65,66]. One study was conducted in each of the following countries: Israel [67], Bosnia-Herzegovina [68], Iran [69], Sweden [70], Argentina [71], and Spain [72]. One multinational study was included [73], which was conducted in the UK, Germany, and the Netherlands. A total of 7333 people with HF were included in this systematic review, with 3681 participants in the experimental group and 3652 in the control. The sample size varied from 20 [39] to 1360 [67]. The mean age of participants in the experimental groups ranged from 50.0 ± 12.0 years [64] to 79.6 ± 6.8 years [47], while that in the control groups ranged from 52.0 ± 8.0 years [64] to 80.9 ± 7.3 years [47].

Regarding clinical characteristics, mean LVEF values in the intervention groups varied across studies. The included participants were predominantly patients with HFrEF (LVEF < 40%) and HFmrEF (40% ≤ LVEF < 50%). Two studies also enrolled patients with HFpEF (LVEF ≥ 50%) [55,67]. Owing to the clinical and methodological heterogeneity across studies, subgroup meta-analyses according to HF phenotype were not performed. Heart failure phenotype, classified according to LVEF, was therefore summarized as a study-level characteristic rather than used for effect estimation. The majority of participants were in NYHA functional class II or III. Two studies [41,73] enrolled patients in whom 61% had NYHA class IV in the experimental group. With regard to comorbidities, hypertension was the most common one, with a prevalence ranging from 19.0% [46] to 91.1% [60]. Diabetes mellitus was the second most frequent comorbidity, with a prevalence ranging from 18.0% [65] to 63.1% [40]. Most of the included studies (*n* = 36) recruited two study arms, while the remaining studies used a three-arm design (*n* = 3) [57,60,73]. The characteristics of all included studies are summarized in Supplementary Table S3.

According to extant meta-analyses [21] and relevant international consensus guidelines [23,24], NPIs comprise 12 intervention categories derived from single and combined applications of 5 core components (Table 1). Multidisciplinary team management was classified only within combined intervention strategies and was not analyzed as a standalone intervention. Control interventions were categorized into UC or NPIs. Supplementary Table S3 details the precise care activities of each intervention, including key component content, session characteristics (number, duration, and frequency), delivery methods, and intervention settings.

### 3.3. Quality Appraisal of Included Studies

Fourteen studies [35,39,41,50,53,57,60,61,62,63,64,69,70,71] were rated as having a high risk of bias, primarily due to bias arising from deviations from intended interventions, incomplete outcome data, and selective reporting. In total, 25 studies [36,37,38,40,42,43,44,45,46,47,48,49,51,54,55,56,58,59,65,66,67,68,72,73] were rated as having some concerns due to the randomization process (lack of information on allocation concealment), lack of blinding, or insufficient information reported. The results of the quality appraisal are presented in Supplementary Figure S5.

### 3.4. Pairwise Comparison on the Effects of NPIs

**Figure 2:**
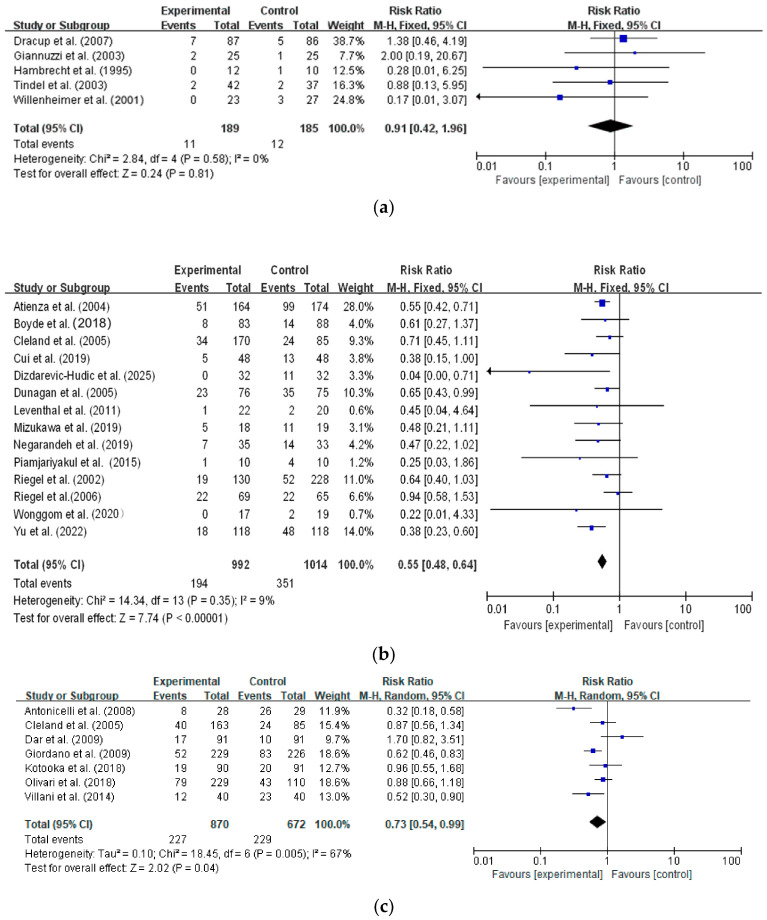

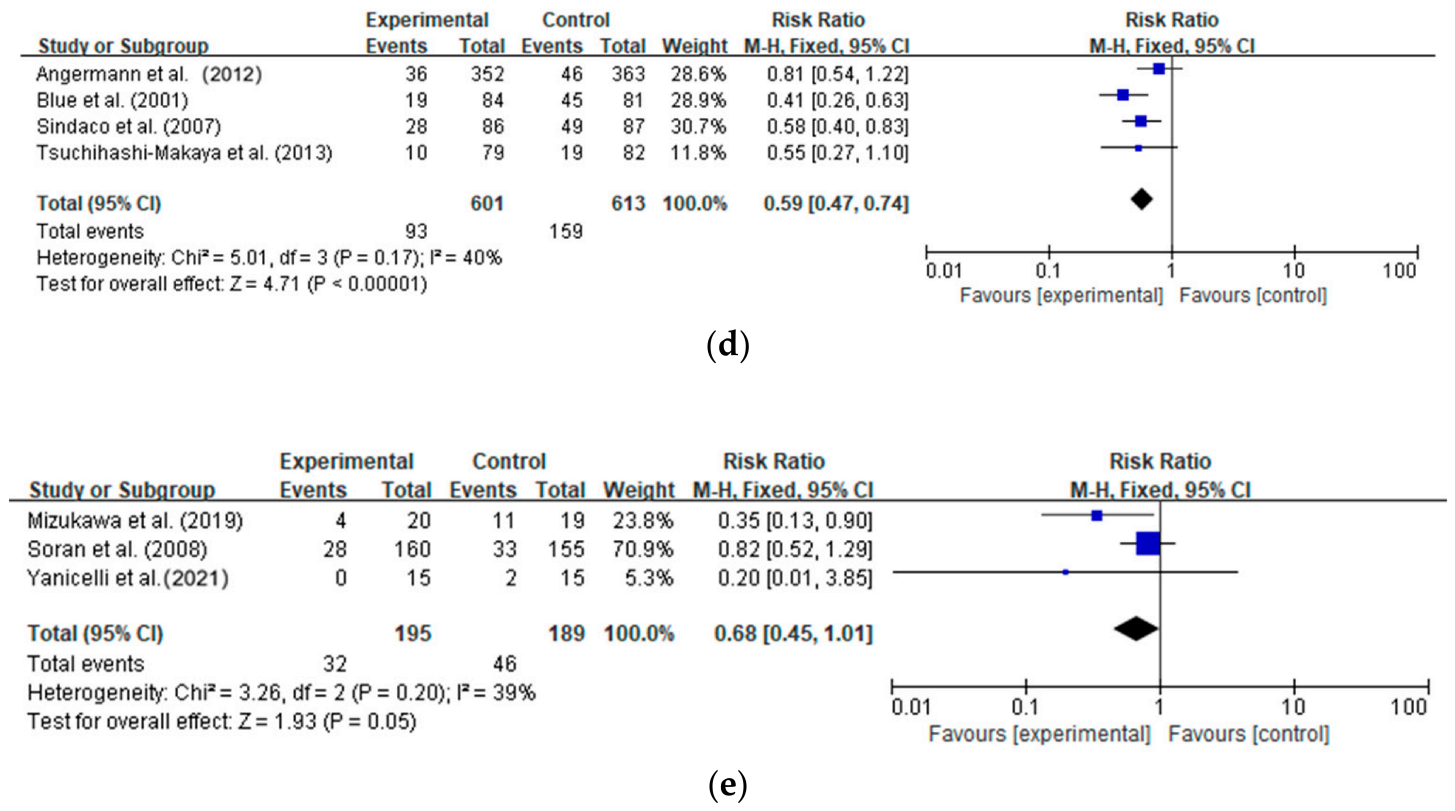
(**a**) Forest plot of pairwise meta-analysis for exercise therapy on HF-related readmissions: RR = 0.91, 95% CI: 0.42–1.96, Fixed-effect model, and *I*^2^ = 0%. (**b**) Forest plot of pairwise meta-analysis for patient education on HF-related readmissions: RR = 0.55, 95% CI: 0.48–1.64, Fixed-effect model, and *I*^2^ = 9%. (**c**) Forest plot of pairwise meta-analysis for health information tracking on HF-related readmissions: RR = 0.73, 95% CI: 0.54–0.99, Random-effect model, and *I*^2^ = 67%. (**d**) Forest plot of pairwise meta-analysis for multidisciplinary team management combined with patient education on HF-related readmissions: RR = 0.59, 95% CI: 0.47–0.74, Fixed-effect model, and *I*^2^ = 40%. (**e**) Forest plot of pairwise meta-analysis for health information tracking combined with patient education on HF-related readmissions: RR = 0.68, 95% CI: 0.45–1.01, Fixed-effect model, and *I*^2^ = 39%. Blue squares represent the weight of each study, with larger squares indicating greater weight. The horizontal lines through the squares denote the 95% confidence intervals for each study’s effect size. The black diamond at the bottom represents the pooled summary estimate from the meta-analysis, with its width indicating the 95% confidence interval of the overall effect [35,37,38,39,40,41,42,43,44,45,46,47,48,51,52,53,55,56,57,58,61,63,64,65,66,68,69,70,71,72,73].

A total of 33 studies were included in the pairwise meta-analysis involving exercise therapy (*n* = 5), patient education (*n* = 14), health information tracking (*n* = 7), multidisciplinary team management combined with patient education (*n* = 4), and patient education combined with health information tracking (*n* = 3) (as some multi-arm studies provided data for more than one comparison, the numbers across intervention categories are not mutually exclusive). Figure 2 shows the forest plots comparing the effects of various NPIs against the control intervention on HF-related unplanned readmissions.

#### 3.4.1. Effects of Patient Education on HF-Related Readmissions

Fourteen trials involving 2006 patients examined the effect of patient education on HF-related readmissions [38,39,40,41,51,52,53,55,57,61,68,69,72,73]. The results indicated that patient education notably decreased HF-related readmissions (RR: 0.55, 95% CI: 0.48 to 0.64, *p* < 0.001, *I*^2^ = 9%, as shown in Figure 2b). Furthermore, the pooled RR remained unchanged following a sensitivity analysis, which demonstrates the stability of these findings (Supplementary Table S4b, Figure S1b). Subgroup analysis revealed that patient education significantly reduced HF-related readmissions during both 3-month and 12-month periods (Supplementary Figure S2(a_1_,c_1_)). However, in the 6-month subgroup, no statistically significant effects were identified. Additionally, Egger’s test showed no evidence of publication bias (*p* = 0.156). The certainty of evidence was rated as high (Supplementary Table S6).

#### 3.4.2. Effects of Health Information Tracking on HF-Related Readmissions

A total of 7 trials involving 1542 patients examined the effect of health information tracking on HF-related readmissions [43,46,47,48,56,66,73]. The results showed that health information tracking significantly reduced HF-related readmissions (RR: 0.73, 95% CI: 0.54 to 0.99, *p* = 0.04, *I*^2^ = 67%, as shown in Figure 2c). Furthermore, the pooled RR remained unchanged following a sensitivity analysis, demonstrating the stability of the findings (Supplementary Table S4c, Figure S1c). A subgroup analysis showed a significant effect of health information tracking on HF-related readmissions when the intervention duration exceeded 12 months (RR: 0.64, 95% CI: 0.46 to 0.89, *p* = 0.008, *I*^2^ = 67%, as shown in Supplementary Figure S2(a_3_)). The certainty of evidence was moderate (Supplementary Table S6).

#### 3.4.3. Effects of Multidisciplinary Team Management Combined with Patient Education on HF-Related Readmissions

Four trials involving 1214 patients examined the effect of multidisciplinary team management plus patient education on HF-related readmissions [44,58,63,65]. The results showed that multidisciplinary team management combined with patient education significantly reduced HF-related readmissions (RR: 0.59, 95% CI: 0.47 to 0.74, *p* < 0.001, *I*^2^ = 40%, as shown in Figure 2d). Furthermore, the pooled RR remained unchanged in the sensitivity analysis, indicating the reliability of the findings (Supplementary Material Table S4d, Figure S1d). Subgroup analysis revealed a significant reduction in HF-related readmissions in studies where the proportion of NYHA class I and II patients was <40% (RR: 0.50, 95% CI: 0.38 to 0.65, *p* < 0.001, *I*^2^ = 33%, Supplementary Figure S2(a_2_)). The certainty of evidence was assessed as moderate (Supplementary Table S6).

Exercise therapy alone and patient education combined with health information tracking did not show statistically significant effects in the pairwise meta-analyses (Figure 2a,e).

### 3.5. NMA on the Comparative Effectiveness of the NPIs

**Figure 3:**
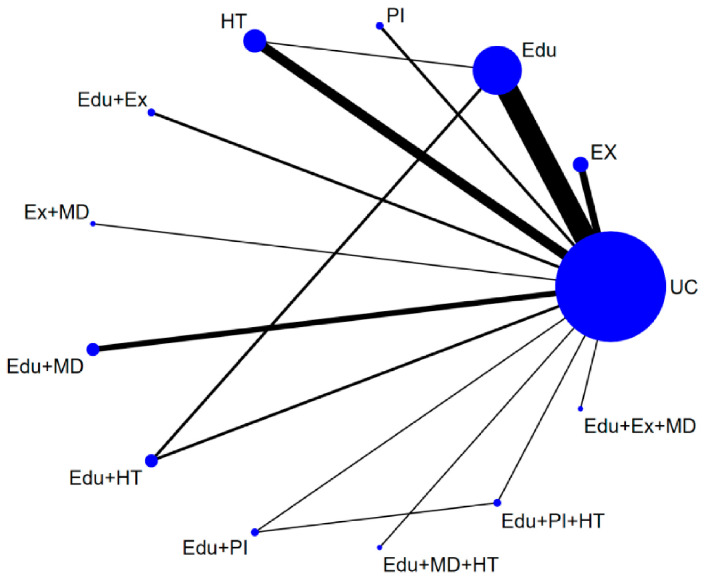
Network plots of NPIs for HF-related readmissions. Abbreviations: UC, Usual Care, Ex, Exercise Therapy, Edu, Patient Education, PI, Psychological Interventions, HT, Health Information Tracking.

**Figure 4:**
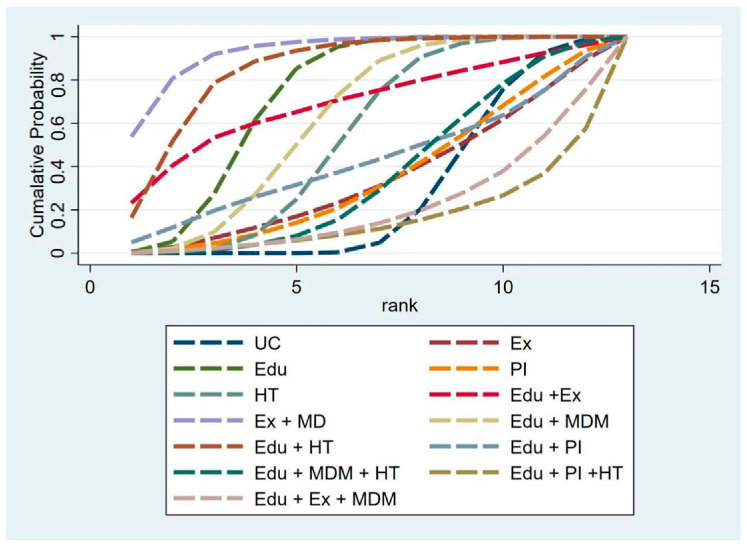
The effectiveness cumulative rank probabilities among different NPIs for HF-related readmissions. Abbreviations: UC, Usual Care, Ex, Exercise Therapy, Edu, Patient Education, PI, Psychological Interventions, HT, Health Information Tracking.

The network geometry for HF-related readmissions is shown in Figure 3, comprising 12 NPIs and 7333 patients. Although the number of studies for some interventions was limited, closed-loop structures were formed. To assess the plausibility of the transitivity assumption, we summarized potential effect modifiers across intervention nodes in Supplementary Table S7, including sample size, mean age, LVEF, NYHA class, comorbidities, intervention frequency and duration, delivery format, intervention setting, and follow-up duration. Overall, age, LVEF, and NYHA class were broadly comparable across most intervention nodes, although differences remained in follow-up duration, intervention intensity, delivery mode, and healthcare setting. Global inconsistency was assessed using the design-by-treatment interaction model in Stata 17.0, which showed no evidence of global inconsistency (*p* = 0.96 > 0.05). For local inconsistency, the node-splitting method revealed no significant differences between direct and indirect comparisons (all *p* > 0.05). Consequently, the consistency model was applied. Table 2 presents the SUCRA rankings, and the cumulative rank probability plots are shown in Figure 4. The league table (Supplementary Table S5) showed that, compared with UC, the following interventions were associated with reduced HF-related readmissions: health information tracking (RR: 0.72, 95% CI: 0.58 to 0.90), exercise therapy combined with multidisciplinary team management (RR: 0.32, 95% CI: 0.15 to 0.65), patient education combined with multidisciplinary team management (RR: 0.57, 95% CI: 0.43 to 0.78), patient education combined with health information tracking (RR: 0.42, 95% CI: 0.25 to 0.72), and patient education (RR: 0.56, 95% CI: 0.46 to 0.69). Furthermore, exercise therapy combined with multidisciplinary team management was associated with a lower risk of HF-related readmissions than psychological interventions (RR: 0.33, 95% CI: 0.12 to 0.89) and health information tracking (RR: 0.44, 95% CI: 0.21 to 0.93) (Supplementary Figure S4). Based on the SUCRA rankings, exercise therapy combined with multidisciplinary team management showed the highest ranking probability (SUCRA = 93.4%), followed by patient education combined with health information tracking (SUCRA = 84.9%). Given the limited number of studies supporting some intervention combinations, these SUCRA-based rankings should be cautiously interpreted as exploratory findings.

### 3.6. Publication Bias

**Figure 5:**
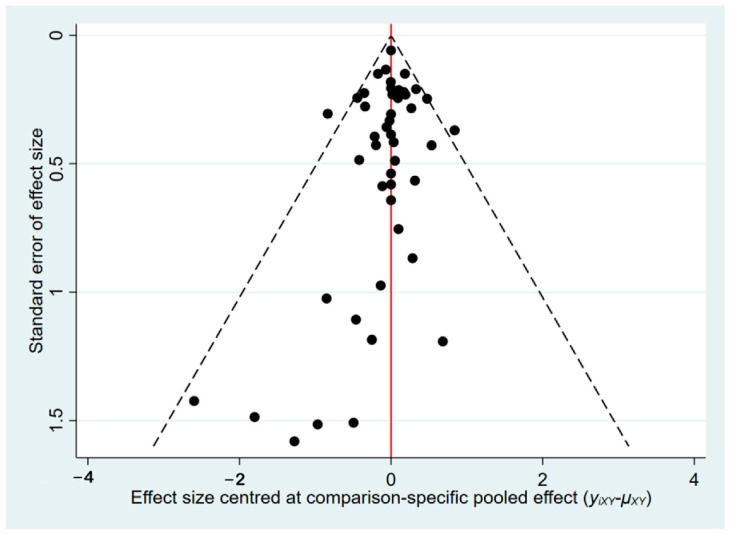
Funnel plot of comparison: effect of different NPIs on HF-related readmissions. Note: the central red vertical line represents the pooled RR value; in the absence of bias, studies should be evenly distributed on both sides of this line. The two diagonal lines on the left and right indicate the confidence region of the funnel plot; ideally, if the plotted points fall within this region, it suggests that heterogeneity may not be a major concern.

The funnel plot, adjusted for comparison, was created to evaluate publication bias, with the effect size (Log RR) displayed along the horizontal axis and the standard error depicted on the vertical axis (see Figure 5). A visual assessment indicated that the study points were mostly symmetrically arranged on either side of the vertical line (X = 0). Although a few studies fell outside the funnel limits, suggesting potential small-study effects or minor publication bias, the overall distribution remained balanced. Furthermore, Egger’s test also revealed no publication bias (*p* = 0.099).

## 4. Discussion

This study evaluated the comparative effectiveness of diverse NPIs in reducing HF-related unplanned readmissions. In the pairwise meta-analysis, patient education, health information tracking, and multidisciplinary team management combined with patient education were associated with significant reductions in HF-related readmissions. In the NMA, exercise therapy combined with multidisciplinary team management had the highest SUCRA ranking, followed by patient education combined with health information tracking. However, these rankings should be interpreted cautiously and regarded as exploratory; because several intervention categories were supported by few studies, most evidence came from comparisons with UC, and direct head-to-head comparisons between active interventions were limited.

Exercise-based multidisciplinary team management, which essentially aligns with the principles of comprehensive exercise-based cardiac rehabilitation (CR), showed the highest SUCRA ranking probability for reducing HF-related readmissions in our analysis [54,74]. This finding is consistent with a 2019 Cochrane review, which demonstrated that patients with HF who participated in exercise-based CR had a 41% reduction in HF-related hospitalization rates [75]. Furthermore, current clinical guidelines have designated CR as a Class I recommendation [76,77]. Despite robust evidence and guideline endorsements, CR remains globally underutilized due to factors such as referral barriers, restrictive coverage, and poor patient adherence [78,79,80]. Emerging solutions such as automatic referral systems, home-based CR, and electronic health (e-health) CR may enhance engagement and accessibility [81,82,83,84]. Li et al. [19] demonstrated that e-health CR nearly doubled the odds of patient adherence (OR: 1.91, 95% CI: 1.34 to 2.73), highlighting its potential to overcome low engagement. Nevertheless, owing to the limited number of studies focusing on exercise-based multidisciplinary team management, our results should be interpreted with caution.

Patient education combined with health information tracking showed a favorable effect on HF-related readmissions in the NMA and ranked second in the SUCRA analysis. However, the direct pairwise meta-analysis did not show a statistically significant advantage over UC, suggesting that the favorable NMA ranking may have been partly influenced by indirect evidence. Therefore, this result should be considered exploratory. Despite this uncertainty, the integrated approach is clinically plausible. In contemporary home-based HF management, health information tracking is often delivered through structured telemonitoring, which may facilitate early detection of clinical deterioration by monitoring body weight, blood pressure, heart rate, and symptoms [85]. This may support timely medication adjustment or urgent outpatient assessment, thereby potentially reducing HF-related readmissions [86,87]. Patient education may further enhance monitoring by improving health literacy, interpretation of physiological data, adherence, and self-management, particularly among older patients who may have difficulty using complex monitoring technologies [88,89,90]. Interactive support such as video consultations or telephone coaching may also reduce anxiety and reinforce positive health behaviors [89]. Nevertheless, although NMA integrates direct and indirect evidence and may improve precision, it cannot fully overcome sparse direct evidence, limited power, or clinical and methodological heterogeneity. Further adequately powered head-to-head trials are needed to confirm whether this combined intervention can reduce HF-related readmissions.

Although patient education did not have the highest SUCRA ranking probability, it ranked relatively highly in the NMA, suggesting that it remains an important component of clinical HF management. This may be attributed to the complex nature of HF self-management, which requires patients to acquire essential skills, such as precise volume management and adherence to dietary restrictions. Structured patient education can deepen patients’ understanding of disease-related knowledge and improve their self-management behaviors [91].

Moreover, the combination of patient education and multidisciplinary team management demonstrated favorable clinical effects, consistent with previous studies [92,93]. In pairwise comparisons, this combined intervention was associated with a significant reduction in HF-related readmissions, and it ranked fourth in the NMA, further supporting its potential value in HF management. Patient education may improve treatment adherence, symptom monitoring, and timely help-seeking behaviors, whereas multidisciplinary team management provides coordinated, continuous, and individualized care through collaboration among healthcare professionals [94,95]. This integrated approach may facilitate early detection of HF deterioration, continuity of post-discharge care, and ultimately reduce the risk of HF-related readmissions.

Our findings have important implications for HF management. Exercise therapy combined with multidisciplinary team management had the highest SUCRA ranking probability for HF-related unplanned readmissions, supporting the potential value of comprehensive exercise-based CR in post-discharge care. However, this ranking should be interpreted cautiously because of the limited number of direct head-to-head comparisons and the uncertainty associated with indirect evidence. Nevertheless, its implementation may be limited by referral barriers, insufficient rehabilitation resources, poor accessibility, and low adherence. Strategies such as automatic referral systems, home-based rehabilitation, e-health CR, and individualized exercise prescriptions may help improve uptake and sustainability. Patient education combined with health information tracking may be considered a clinically plausible strategy, but its high NMA ranking should be interpreted cautiously because direct pairwise evidence did not show a statistically significant effect. In clinical practice, monitoring systems should be combined with structured education, timely feedback, and professional decision-making rather than used as passive data-collection tools. Such an integrated approach may improve patients’ interpretation of physiological changes, promote self-management, and enable early intervention for HF deterioration.

Beyond readmission prevention, health information tracking and telemonitoring may also support risk stratification and transitional management in selected high-risk patients. In patients with newly diagnosed or transient HFrEF and LVEF < 35%, the period of guideline-directed medical therapy optimization is clinically vulnerable because left ventricular function may recover in some patients, whereas others remain at risk of ventricular arrhythmias, sudden cardiac death, and HF decompensation before reassessment. Although not directly evaluated in our NMA, wearable cardioverter-defibrillators may provide temporary protection during this period and help avoid unnecessary implantable cardioverter-defibrillator implantation [96,97].

Several limitations should be considered. First, most included studies compared active interventions with usual care, whereas direct head-to-head comparisons between active non-pharmacological interventions were limited. This may have reduced the robustness of indirect comparisons in the network meta-analysis. Future trials should prioritize direct comparisons among active interventions. Second, several intervention categories were supported by only a small number of studies with limited sample sizes, resulting in imprecise effect estimates and uncertainty in ranking probabilities. This was particularly evident for patient education combined with health information tracking, which ranked highly in the NMA but did not show a statistically significant effect in pairwise analyses. Third, clinical and methodological heterogeneity remained across studies, despite the assessment of key effect modifiers. Sources of heterogeneity included differences in intervention intensity, delivery format, healthcare setting, follow-up duration, and outcome definitions, as well as incomplete reporting of comorbidities and HF phenotypes. Accordingly, SUCRA rankings should be interpreted as exploratory. Future research should standardize intervention and outcome definitions and improve the reporting of intervention implementation and patient-level characteristics. Finally, blinding was often not feasible in trials of non-pharmacological interventions, which may have introduced performance bias. Future studies should strengthen methodological rigor where possible and incorporate digital biomarkers and wearable sensor-derived measures, such as step counts and heart rate variability, to obtain continuous and objective data rather than relying solely on patient-reported logs.

## 5. Conclusions

This systematic review and NMA suggest that several NPIs may reduce HF-related unplanned readmissions. Exercise therapy combined with multidisciplinary team management had the highest SUCRA ranking probability, followed by patient education combined with health information tracking. Patient education and patient education combined with multidisciplinary team management also showed potential benefits. However, given the limited number of head-to-head comparisons, heterogeneity across studies, sparse direct evidence for some interventions, and low certainty of evidence for several comparisons, these results, particularly SUCRA-based rankings, should be interpreted as exploratory. Future large-scale, multicenter randomized controlled trials are needed to directly compare active NPIs and evaluate their long-term effectiveness, safety, cost effectiveness, and implementation feasibility.

## Figures and Tables

**Table 1:** NPIs for reducing HF-related readmissions.

Types of NPIs	Abbreviation	Included Studies	Explanation
Exercise therapy	Ex	(*n* = 5) [35,37,45,64,70]	Exercise therapy refers to an individualized and progressive program of physical activities designed for patients under the guidance of rehabilitation professionals.
Patient education	Edu	(*n* = 14) [38,39,40,41,51,52,53,55,57,61,68,69,72,73]	Patient education refers to the systematic imparting of disease-related knowledge and self-management skills to patients through face-to-face instruction, distribution of manuals, or multimedia formats.
Psychological interventions	PI	(*n* = 2) [36,59]	Psychological intervention refers to the professional strategies implemented to address the negative psychological states and cognition.
Health information tracking	HT	(*n* = 7) [43,46,47,48,56,66,73]	Health information tracking refers to a structured home-based telemonitoring intervention involving the real-time recording, monitoring, and transmission of patient health data, such as symptoms, body weight, vital signs, or other physiological data, for remote clinical review, timely decision-making, and early outpatient intervention when deterioration is suspected.
Multidisciplinary team management	MD	/	Multidisciplinary team management involves a team composed of cardiologists, specialist nurses, pharmacists, dietitians, and rehabilitation therapists who collaboratively develop a holistic and personalized management plan for patients.
Patient education + Exercise therapy	Edu + Ex	(*n* = 2) [50,62]	/
Exercise therapy + Multidisciplinary team management	Ex + MD	(*n* = 1) [54]	/
Multidisciplinary team management + Patient education	Edu + MD	(*n* = 4) [44,58,63,65]	/
Patient education + Health information tracking	Edu + HT	(*n* = 3) [42,57,71]	/
Patient education + Psychological interventions	Edu + PI	(*n* = 1) [60]	/
Patient education + Multidisciplinary team management + Health information tracking	Edu + MD + HT	(*n* = 1) [67]	/
Patient education + Psychological interventions + Health information tracking	Edu + PI + HT	(*n* = 1) [60]	/
Patient education + Multidisciplinary team management + Exercise therapy	Edu + Ex + MD	(*n* = 1) [49]	/

**Table 2:** Rank probabilities of SUCRA Value.

Types of NPIs	SUCRA Value of the HF-Related Readmissions	Rank
Ex + MD	93.4	1
Edu + HT	84.9	2
Edu	71.3	3
Edu + MD	70.5	4
Edu + Ex	69.6	5
HT	52.9	6
Edu + PI	41.4	7
Edu + MD + HT	35.0	8
PI	34.4	9
Ex	33.4	10
UC	28.2	11
Edu + Ex + MD	20.3	12
Edu + PI + HT	14.7	13

Abbreviations: UC, Usual Care, Ex, Exercise Therapy, Edu, Patient Education, PI, Psychological Interventions, HT, Health Information Tracking.

## Data Availability

The data supporting the findings of this study are available from the corresponding author upon reasonable request.

## Supplementary Materials

The following supporting information can be downloaded at: https://www.mdpi.com/article/10.3390/jcm15145344/s1. Table S1. PRISMA Checklist; Table S2. Search strategy; Table S3. Characteristics of included studies; Table S4. Sensitivity analysis of NPIs effects on HF-related readmission rates. (a) Exercise therapy, (b) patient education, (c) health information tracking, (d) integrated care + patient education, and (e) health information tracking + patient education; Table S5. Network Meta-Analysis of Effectiveness of NPIs (RR and 95%CI); Table S6. The evidence findings for all comparisons; Table S7. Transitivity assessment of potential effect modifiers across intervention nodes; Figure S1. Sensitivity analysis of NPIs effects on HF-related readmission rates. (a) Exercise therapy, (b) patient education, (c) health information tracking, (d) integrated care + patient education, and (e) health information tracking + patient education; Figure S2. Subgroup analyses of the effects of NPIs on HF-related readmission rates. Panels (a1, b1, c1) show the effects of patient education stratified by intervention duration: (a1) 12 months, (b1) 6 months, and (c1) 3 months. Panels (a2 and b2) show integrated care + patient education stratified by the proportion of NYHA class I/II patients: (a2) <40% and (b2) >40%. Panels (a3 and b3) show health information tracking stratified by intervention duration: (a3) 6 months and (b3) ≥12 months. Note: All comparisons are versus usual care; Figure S3. Funnel plots for the effects of patient education on HF-related readmission rates; Figure S4. Forest plots for NPIs on overall HF-related readmission rates; Figure S5. (a) Risk of bias summary; (b) Risk of bias graph.

## Abbreviations

The following abbreviations are used in this manuscript:

HF	Heart failure
NPIs	Non-pharmacological interventions
NMA	Network meta-analysis
RCTs	Randomized controlled trials
UC	Usual care
CR	Cardiac rehabilitation
e-health	electronic health
MeSH	Medical Subject Headings
ITT	Intention-to-treat
CIs	Confidence intervals
RR	Risk ratio
SUCRA	Surface under the cumulative ranking curve
RoB 2	Revised Cochrane risk-of-bias tool for randomized trials
GRADE	Grading of Recommendations Assessment, Development and Evaluation
LVEF	Left ventricular ejection fraction
NYHA	New York Heart Association
HFrEF	Heart failure with reduced ejection fraction
HFmrEF	Heart failure with mildly reduced ejection fraction
HFpEF	Heart failure with preserved ejection fraction
ICD	Implantable cardioverter-defibrillator
WCD	Wearable cardioverter-defibrillator

## References

[1] Savarese G., Becher P.M., Lund L.H., Seferovic P., Rosano G.M.C., Coats A.J.S. (2023). Global burden of heart failure: A comprehensive and updated review of epidemiology. Cardiovasc. Res..

[2] Wang H., Zhou H., Chai K., Yan S., Liu Y., Kuhn M., Prettner K., Cao Z., Du M., Wang T. (2026). Heart failure in China: A macroeconomic modelling study of intervention strategies. Eur. Heart J..

[3] Liu S., Graves N., Ma C., Pan J., Xie Y., Lee S.Y.A., Senanayake S., Kularatna S. (2025). Preventability of readmissions for patients with heart failure-A scoping review. Heart Lung.

[4] Kimmoun A., Takagi K., Gall E., Ishihara S., Hammoum P., El Bèze N., Bourgeois A., Chassard G., Pegorer-Sfes H., Gayat E. (2021). Temporal trends in mortality and readmission after acute heart failure: A systematic review and meta-regression in the past four decades. Eur. J. Heart Fail..

[5] Lan T., Liao Y.H., Zhang J., Yang Z.P., Xu G.S., Zhu L., Fan D.M. (2021). Mortality and Readmission Rates After Heart Failure: A Systematic Review and Meta-Analysis. Ther. Clin. Risk Manag..

[6] Eltelbany M., Chan S., Gottlieb S. (2019). Specific Causes of 30-Day and 1-Year Readmissions in Heart Failure Patients. J. Card. Fail..

[7] Khan M.S., Sreenivasan J., Lateef N., Abougergi M.S., Greene S.J., Ahmad T., Anker S.D., Fonarow G.C., Butler J. (2021). Trends in 30- and 90-Day Readmission Rates for Heart Failure. Circ. Heart Fail..

[8] Lahoz R., Fagan A., McSharry M., Proudfoot C., Corda S., Studer R. (2020). Recurrent heart failure hospitalizations are associated with increased cardiovascular mortality in patients with heart failure in Clinical Practice Research Datalink. ESC Heart Fail..

[9] Lindmark K., Boman K., Stålhammar J., Olofsson M., Lahoz R., Studer R., Proudfoot C., Corda S., Fonseca A.F., Costa-Scharplatz M. (2021). Recurrent heart failure hospitalizations increase the risk of cardiovascular and all-cause mortality in patients with heart failure in Sweden: A real-world study. ESC Heart Fail..

[10] Ashokkumar S., Teperman J., Russo J.J., Brown A., Jaijee S. (2025). Qualitative Content Analysis of Unplanned Readmissions in Patients With Acute Heart Failure. Heart Lung Circ..

[11] Hessel F.P. (2021). Overview of the socio-economic consequences of heart failure. Cardiovasc. Diagn. Ther..

[12] Tsao C.W., Aday A.W., Almarzooq Z.I., Alonso A., Beaton A.Z., Bittencourt M.S., Boehme A.K., Buxton A.E., Carson A.P., Commodore-Mensah Y. (2022). Heart Disease and Stroke Statistics-2022 Update: A Report From the American Heart Association. Circulation.

[13] Rasmussen A.A., Wiggers H., Jensen M., Berg S.K., Rasmussen T.B., Borregaard B., Thrysoee L., Thorup C.B., Mols R.E., Larsen S.H. (2021). Patient-reported outcomes and medication adherence in patients with heart failure. Eur. Heart J. Cardiovasc. Pharmacother..

[14] Fibbi G., Sato R., Vatic M., Genreith F.P., von Haehling S. (2024). Pharmacological management of heart failure: A patient-centred approach. Expert. Opin. Pharmacother..

[15] Tian C., Zhang J., Rong J., Ma W., Yang H. (2024). Impact of nurse-led education on the prognosis of heart failure patients: A systematic review and meta-analysis. Int. Nurs. Rev..

[16] Molloy C.D., Long L., Mordi I.R., Bridges C., Sagar V.A., Davies E.J., Coats A.J.S., Dalal H., Rees K., Singh S.J. (2023). Exercise-based cardiac rehabilitation for adults with heart failure—2023 Cochrane systematic review and meta-analysis. Eur. J. Heart Fail..

[17] Ski C.F., Taylor R.S., McGuigan K., Long L., Lambert J.D., Richards S.H., Thompson D.R. (2024). Psychological interventions for depression and anxiety in patients with coronary heart disease, heart failure or atrial fibrillation. Cochrane Database Syst. Rev..

[18] Alnomasy N., Still C.H. (2023). Nonpharmacological Interventions for Preventing Rehospitalization Among Patients with Heart Failure: A Systematic Review and Meta-Analysis. SAGE Open Nurs..

[19] Li Y., He W., Jiang J., Zhang J., Ding M., Li G., Luo X., Ma Z., Li J., Ma Y. (2024). Non-Pharmacological Interventions in Patients With Heart Failure With Reduced Ejection Fraction: A Systematic Review and Network Meta-analysis. Arch. Phys. Med. Rehabil..

[20] Sun Y., Ji M., Leng M., Li X., Zhang X., Wang Z. (2022). Comparative efficacy of 11 non-pharmacological interventions on depression, anxiety, quality of life, and caregiver burden for informal caregivers of people with dementia: A systematic review and network meta-analysis. Int. J. Nurs. Stud..

[21] Zhang N., Li Q., Chen S., Wu Y., Xin B., Wan Q., Shi P., He Y., Yang S., Jiang W. (2024). Effectiveness of nurse-led electronic health interventions on illness management in patients with chronic heart failure: A systematic review and meta-analysis. Int. J. Nurs. Stud..

[22] Hutton B., Salanti G., Caldwell D.M., Chaimani A., Schmid C.H., Cameron C., Ioannidis J.P., Straus S., Thorlund K., Jansen J.P. (2015). The PRISMA extension statement for reporting of systematic reviews incorporating network meta-analyses of health care interventions: Checklist and explanations. Ann. Intern. Med..

[23] Jaarsma T., Hill L., Bayes-Genis A., La Rocca H.B., Castiello T., Čelutkienė J., Marques-Sule E., Plymen C.M., Piper S.E., Riegel B. (2021). Self-care of heart failure patients: Practical management recommendations from the Heart Failure Association of the European Society of Cardiology. Eur. J. Heart Fail..

[24] Heidenreich P.A., Bozkurt B., Aguilar D., Allen L.A., Byun J.J., Colvin M.M., Deswal A., Drazner M.H., Dunlay S.M., Evers L.R. (2022). 2022 AHA/ACC/HFSA Guideline for the Management of Heart Failure: A Report of the American College of Cardiology/American Heart Association Joint Committee on Clinical Practice Guidelines. Circulation.

[25] Sterne J., Savović J., Page M., Elbers R., Blencowe N., Boutron I., Cates C., Cheng H.-Y., Corbett M., Eldridge S. (2019). RoB 2: A revised tool for assessing risk of bias in randomised trials. BMJ.

[26] McGuinness L.A., Higgins J.P.T. (2021). Risk-of-bias VISualization (robvis): An R package and Shiny web app for visualizing risk-of-bias assessments. Res. Synth. Methods.

[27] Patsopoulos N.A., Evangelou E., Ioannidis J.P. (2008). Sensitivity of between-study heterogeneity in meta-analysis: Proposed metrics and empirical evaluation. Int. J. Epidemiol..

[28] Sterne J.A., Sutton A.J., Ioannidis J.P., Terrin N., Jones D.R., Lau J., Carpenter J., Rücker G., Harbord R.M., Schmid C.H. (2011). Recommendations for examining and interpreting funnel plot asymmetry in meta-analyses of randomised controlled trials. BMJ.

[29] Salanti G., Del Giovane C., Chaimani A., Caldwell D.M., Higgins J.P. (2014). Evaluating the quality of evidence from a network meta-analysis. PLoS ONE.

[30] Dias S., Welton N.J., Caldwell D.M., Ades A.E. (2010). Checking consistency in mixed treatment comparison meta-analysis. Stat. Med..

[31] Higgins J.P., Jackson D., Barrett J.K., Lu G., Ades A.E., White I.R. (2012). Consistency and inconsistency in network meta-analysis: Concepts and models for multi-arm studies. Res. Synth. Methods.

[32] Brooks S.P., Gelman A. (1998). General Methods for Monitoring Convergence of Iterative Simulations. J. Comput. Graph. Stat..

[33] Egger M., Davey Smith G., Schneider M., Minder C. (1997). Bias in meta-analysis detected by a simple, graphical test. BMJ.

[34] Sedgwick P., Marston L. (2015). How to read a funnel plot in a meta-analysis. BMJ.

[35] Corvera-Tindel T., Doering L.V., Woo M.A., Khan S., Dracup K. (2004). Effects of a home walking exercise program on functional status and symptoms in heart failure. Am. Heart J..

[36] Dekker R.L., Moser D.K., Peden A.R., Lennie T.A. (2012). Cognitive therapy improves three-month outcomes in hospitalized patients with heart failure. J. Card. Fail..

[37] Dracup K., Evangelista L.S., Hamilton M.A., Erickson V., Hage A., Moriguchi J., Canary C., MacLellan W.R., Fonarow G.C. (2007). Effects of a home-based exercise program on clinical outcomes in heart failure. Am. Heart J..

[38] Dunagan W.C., Littenberg B., Ewald G.A., Jones C.A., Emery V.B., Waterman B.M., Silverman D.C., Rogers J.G. (2005). Randomized trial of a nurse-administered, telephone-based disease management program for patients with heart failure. J. Card. Fail..

[39] Piamjariyakul U., Werkowitch M., Wick J., Russell C., Vacek J.L., Smith C.E. (2015). Caregiver coaching program effect: Reducing heart failure patient rehospitalizations and improving caregiver outcomes among African Americans. Heart Lung.

[40] Riegel B., Carlson B., Glaser D., Romero T. (2006). Randomized controlled trial of telephone case management in Hispanics of Mexican origin with heart failure. J. Card. Fail..

[41] Riegel B., Carlson B., Kopp Z., LePetri B., Glaser D., Unger A. (2002). Effect of a standardized nurse case-management telephone intervention on resource use in patients with chronic heart failure. Arch. Intern. Med..

[42] Soran O.Z., Pina I.L., Lamas G.A., Kelsey S.F., Selzer F., Pilotte J., Lave J.R., Feldman A.M. (2008). A randomized clinical trial of the clinical effects of enhanced heart failure monitoring using a computer-based telephonic monitoring system in older minorities and women. J. Card. Fail..

[43] Antonicelli R., Testarmata P., Spazzafumo L., Gagliardi C., Bilo G., Valentini M., Olivieri F., Parati G. (2008). Impact of telemonitoring at home on the management of elderly patients with congestive heart failure. J. Telemed. Telecare.

[44] Del Sindaco D., Pulignano G., Minardi G., Apostoli A., Guerrieri L., Rotoloni M., Petri G., Fabrizi L., Caroselli A., Venusti R. (2007). Two-year outcome of a prospective, controlled study of a disease management programme for elderly patients with heart failure. J. Cardiovasc. Med..

[45] Giannuzzi P., Temporelli P.L., Corra U., Tavazzi L., Group E.-C.S. (2003). Antiremodeling effect of long-term exercise training in patients with stable chronic heart failure: Results of the Exercise in Left Ventricular Dysfunction and Chronic Heart Failure (ELVD-CHF) Trial. Circulation.

[46] Giordano A., Scalvini S., Zanelli E., Corra U., Longobardi G.L., Ricci V.A., Baiardi P., Glisenti F. (2009). Multicenter randomised trial on home-based telemanagement to prevent hospital readmission of patients with chronic heart failure. Int. J. Cardiol..

[47] Olivari Z., Giacomelli S., Gubian L., Mancin S., Visentin E., Di Francesco V., Iliceto S., Penzo M., Zanocco A., Marcon C. (2018). The effectiveness of remote monitoring of elderly patients after hospitalisation for heart failure: The renewing health European project. Int. J. Cardiol..

[48] Villani A., Malfatto G., Compare A., Della Rosa F., Bellardita L., Branzi G., Molinari E., Parati G. (2014). Clinical and psychological telemonitoring and telecare of high risk heart failure patients. J. Telemed. Telecare.

[49] Chen Y., Funk M., Wen J., Tang X., He G., Liu H. (2018). Effectiveness of a multidisciplinary disease management program on outcomes in patients with heart failure in China: A randomized controlled single center study. Heart Lung.

[50] Chen Y.W., Wang C.Y., Lai Y.H., Liao Y.C., Wen Y.K., Chang S.T., Huang J.L., Wu T.J. (2018). Home-based cardiac rehabilitation improves quality of life, aerobic capacity, and readmission rates in patients with chronic heart failure. Medicine.

[51] Cui X., Zhou X., Ma L.L., Sun T.W., Bishop L., Gardiner F.W., Wang L. (2019). A nurse-led structured education program improves self-management skills and reduces hospital readmissions in patients with chronic heart failure: A randomized and controlled trial in China. Rural. Remote Health.

[52] Yu D.S., Li P.W., Li S.X., Smith R.D., Yue S.C., Yan B.P.Y. (2022). Effectiveness and Cost-effectiveness of an Empowerment-Based Self-care Education Program on Health Outcomes Among Patients With Heart Failure: A Randomized Clinical Trial. JAMA Netw. Open.

[53] Boyde M., Peters R., New N., Hwang R., Ha T., Korczyk D. (2018). Self-care educational intervention to reduce hospitalisations in heart failure: A randomised controlled trial. Eur. J. Cardiovasc. Nurs..

[54] Davidson P.M., Cockburn J., Newton P.J., Webster J.K., Betihavas V., Howes L., Owensby D.O. (2010). Can a heart failure-specific cardiac rehabilitation program decrease hospitalizations and improve outcomes in high-risk patients?. Eur. J. Prev. Cardiol..

[55] Wonggom P., Nolan P., Clark R.A., Barry T., Burdeniuk C., Nesbitt K., O’Toole K., Du H. (2020). Effectiveness of an avatar educational application for improving heart failure patients’ knowledge and self-care behaviors: A pragmatic randomized controlled trial. J. Adv. Nurs..

[56] Kotooka N., Kitakaze M., Nagashima K., Asaka M., Kinugasa Y., Nochioka K., Mizuno A., Nagatomo D., Mine D., Yamada Y. (2018). The first multicenter, randomized, controlled trial of home telemonitoring for Japanese patients with heart failure: Home telemonitoring study for patients with heart failure (HOMES-HF). Heart Vessel..

[57] Mizukawa M., Moriyama M., Yamamoto H., Rahman M.M., Naka M., Kitagawa T., Kobayashi S., Oda N., Yasunobu Y., Tomiyama M. (2019). Nurse-Led Collaborative Management Using Telemonitoring Improves Quality of Life and Prevention of Rehospitalization in Patients with Heart Failure. Int. Heart J..

[58] Tsuchihashi-Makaya M., Matsuo H., Kakinoki S., Takechi S., Kinugawa S., Tsutsui H. (2013). for the J-HOMECARE Investigators. Home-based disease management program to improve psychological status in patients with heart failure in Japan. Circ. J..

[59] Chew H.S.J., Sim K.L.D., Choi K.C., Chair S.Y. (2021). Effectiveness of a nurse-led temporal self-regulation theory-based program on heart failure self-care: A randomized controlled trial. Int. J. Nurs. Stud..

[60] Jiang Y., Koh K.W.L., Ramachandran H.J., Nguyen H.D., Lim S., Tay Y.K., Shorey S., Wang W. (2021). The effectiveness of a nurse-led home-based heart failure self-management programme (the HOM-HEMP) for patients with chronic heart failure: A three-arm stratified randomized controlled trial. Int. J. Nurs. Stud..

[61] Leventhal M.E., Denhaerynck K., Brunner-La Rocca H.P., Burnand B., Conca-Zeller A., Bernasconi A.T., Mahrer-Imhof R., Froelicher E.S., De Geest S. (2011). Swiss Interdisciplinary Management Programme for Heart Failure (SWIM-HF): A randomised controlled trial study of an outpatient inter-professional management programme for heart failure patients in Switzerland. Swiss Med. Wkly..

[62] Mueller L., Myers J., Kottman W., Oswald U., Boesch C., Arbrol N., Dubach P. (2007). Exercise capacity, physical activity patterns and outcomes six years after cardiac rehabilitation in patients with heart failure. Clin. Rehabil..

[63] Angermann C.E., Stork S., Gelbrich G., Faller H., Jahns R., Frantz S., Loeffler M., Ertl G. (2012). Mode of action and effects of standardized collaborative disease management on mortality and morbidity in patients with systolic heart failure: The Interdisciplinary Network for Heart Failure (INH) study. Circ. Heart Fail..

[64] Hambrecht R., Niebauer J., Fiehn E., Kälberer B., Offner B., Hauer K., Riede U., Schlierf G., Kübler W., Schuler G. (1995). Physical training in patients with stable chronic heart failure: Effects on cardiorespiratory fitness and ultrastructural abnormalities of leg muscles. J. Am. Coll. Cardiol..

[65] Blue L., Lang E., McMurray J.J., Davie A.P., McDonagh T.A., Murdoch D.R., Petrie M.C., Connolly E., Norrie J., Round C.E. (2001). Randomised controlled trial of specialist nurse intervention in heart failure. BMJ.

[66] Dar O., Riley J., Chapman C., Dubrey S.W., Morris S., Rosen S.D., Roughton M., Cowie M.R. (2009). A randomized trial of home telemonitoring in a typical elderly heart failure population in North West London: Results of the Home-HF study. Eur. J. Heart Fail..

[67] Kalter-Leibovici O., Freimark D., Freedman L.S., Kaufman G., Ziv A., Murad H., Benderly M., Silverman B.G., Friedman N., Cukierman-Yaffe T. (2017). Disease management in the treatment of patients with chronic heart failure who have universal access to health care: A randomized controlled trial. BMC Med..

[68] Dizdarevic-Hudic L., Halilovic E., Brkic S., Loncar D., Avdic Jahic N., Hudic I., Mujic Ibralic A., Suljic Z. (2025). The Impact of Patient Education on Rehospitalization Rate and Quality of Life in Heart Failure Patients. Int. J. Cardiovasc. Acad..

[69] Negarandeh R., Zolfaghari M., Bashi N., Kiarsi M. (2019). Evaluating the Effect of Monitoring through Telephone (Tele-Monitoring) on Self-Care Behaviors and Readmission of Patients with Heart Failure after Discharge. Appl. Clin. Inform..

[70] Willenheimer R., Rydberg E., Cline C., Broms K., Hillberger B., Oberg L., Erhardt L. (2001). Effects on quality of life, symptoms and daily activity 6 months after termination of an exercise training programme in heart failure patients. Int. J. Cardiol..

[71] Yanicelli L.M., Goy C.B., González V.D.C., Palacios G.N., Martínez E.C., Herrera M.C. (2021). Non-invasive home telemonitoring system for heart failure patients: A randomized clinical trial. J. Telemed. Telecare.

[72] Atienza F., Anguita M., Martinez-Alzamora N., Osca J., Ojeda S., Almenar L., Ridocci F., Vallés F., de Velasco J.A. (2004). Multicenter randomized trial of a comprehensive hospital discharge and outpatient heart failure management program. Eur. J. Heart Fail..

[73] Cleland J.G., Louis A.A., Rigby A.S., Janssens U., Balk A.H. (2005). Noninvasive home telemonitoring for patients with heart failure at high risk of recurrent admission and death: The Trans-European Network-Home-Care Management System (TEN-HMS) study. J. Am. Coll. Cardiol..

[74] Bozkurt B., Fonarow G.C., Goldberg L.R., Guglin M., Josephson R.A., Forman D.E., Lin G., Lindenfeld J., O’cOnnor C., Panjrath G. (2021). Cardiac Rehabilitation for Patients With Heart Failure. JACC.

[75] Long L., Mordi I.R., Bridges C., Sagar V.A., Davies E.J., Coats A.J., Dalal H., Rees K., Singh S.J., Taylor R.S. (2019). Exercise-based cardiac rehabilitation for adults with heart failure. Cochrane Database Syst. Rev..

[76] Yancy C.W., Jessup M., Bozkurt B., Butler J., Casey D.E., Drazner M.H., Fonarow G.C., Geraci S.A., Horwich T., Januzzi J.L. (2013). 2013 ACCF/AHA guideline for the management of heart failure: A report of the American College of Cardiology Foundation/American Heart Association Task Force on practice guidelines. Circulation.

[77] Piepoli M.F. (2017). 2016 European Guidelines on cardiovascular disease prevention in clinical practice: The Sixth Joint Task Force of the European Society of Cardiology and Other Societies on Cardiovascular Disease Prevention in Clinical Practice (constituted by representatives of 10 societies and by invited experts). Int. J. Behav. Med..

[78] Bracewell N.J., Plasschaert J., Conti C.R., Keeley E.C., Conti J.B. (2022). Cardiac rehabilitation: Effective yet underutilized in patients with cardiovascular disease. Clin. Cardiol..

[79] Zhang Y., Huang S., Liu B., Li Y. (2025). Seize the advantages of cardiac rehabilitation. Ann. Med..

[80] Khadanga S., Savage P., Keteyian S., Yant B., Gaalema D., Ades P. (2024). Cardiac rehabilitation: The gateway for secondary prevention. Heart.

[81] Supervía M., Medina-Inojosa J.R., Yeung C., Lopez-Jimenez F., Squires R.W., Pérez-Terzic C.M., Brewer L.C., Leth S.E., Thomas R.J. (2017). Cardiac Rehabilitation for Women: A Systematic Review of Barriers and Solutions. Mayo Clin. Proc..

[82] Grace S.L., Kotseva K., Whooley M.A. (2021). Cardiac Rehabilitation: Under-Utilized Globally. Curr. Cardiol. Rep..

[83] Mathews L., Brewer L.C. (2021). A Review of Disparities in Cardiac Rehabilitation: EVIDENCE, DRIVERS, AND SOLUTIONS. J. Cardiopulm. Rehabil. Prev..

[84] Taylor R.S., Dalal H.M., McDonagh S.T.J. (2022). The role of cardiac rehabilitation in improving cardiovascular outcomes. Nat. Rev. Cardiol..

[85] De Lathauwer I.L.J., Nieuwenhuys W.W., Hafkamp F., Regis M., Brouwers R.W.M., Funk M., Kemps H.M.C. (2025). Remote patient monitoring in heart failure: A comprehensive meta-analysis of effective programme components for hospitalization and mortality reduction. Eur. J. Heart Fail..

[86] Stevenson L., Ross H., Rathman L., Boehmer J. (2023). Remote Monitoring for Heart Failure Management at Home. J. Am. Coll. Cardiol..

[87] Matteucci A., Bonanni M., Centioni M., Zanin F., Geuna F., Massaro G., Sangiorgi G. (2021). Home Management of Heart Failure and Arrhythmias in Patients with Cardiac Devices during Pandemic. J. Clin. Med..

[88] Ong M.K., Romano P.S., Edgington S., Aronow H.U., Auerbach A.D., Black J.T., De Marco T., Escarce J.J., Evangelista L.S., Hanna B. (2016). Effectiveness of Remote Patient Monitoring After Discharge of Hospitalized Patients With Heart Failure: The Better Effectiveness After Transition -- Heart Failure (BEAT-HF) Randomized Clinical Trial. JAMA Intern. Med..

[89] Black J.T., Romano P.S., Sadeghi B., Auerbach A.D., Ganiats T.G., Greenfield S., Kaplan S.H., Ong M.K. (2014). A remote monitoring and telephone nurse coaching intervention to reduce readmissions among patients with heart failure: Study protocol for the Better Effectiveness After Transition-Heart Failure (BEAT-HF) randomized controlled trial. Trials.

[90] Maguire R., Connaghan J., Arber A., Klepacz N., Blyth K.G., McPhelim J., Murray P., Rupani H., Chauhan A., Williams P. (2020). Advanced Symptom Management System for Patients with Malignant Pleural Mesothelioma (ASyMSmeso): Mixed Methods Study. J. Med. Internet Res..

[91] Saharan P., Srinivasa Rao V. (2025). Evaluating the effectiveness of digital health interventions on reduci ng hospital readmissions in heart failure patients. J. Neonatal Surg..

[92] Chan C., Li P.W.-C., Lee D., Fong E., Ng I., Chiu S.K.-M., Fok C.W.-S., Li F.K.-W., Lee S., Ho K. (2025). Nurse-coordinated multidisciplinary comprehensive heart failure management programme: A propensity-matched trial. ESC Heart Fail..

[93] Hajaj A., Abdel-Rahman M., Babu G., Hadi M., Badr A., Turk-Adawi K. (2025). The impact of multidisciplinary disease management programmes on readmissions in heart failure: A systematic review and meta-analysis. Eur. J. Cardiovasc. Nurs..

[94] Ghobadi P., Gholami M., Hasanvand S., Toulabi T., Moradifar N., Birjandi M. (2022). Effects of a multidisciplinary management program on symptom burden and medication adherence in heart failure patients with comorbidities: A randomized controlled trial. BMC Nurs..

[95] Hajaj A., Abdel-Rahman M., Hadi A., Badr A., Turk-Adawi K. (2026). Does multidisciplinary disease management program lower hospitalization and mortality among patients with heart failure? A systematic review and meta-analysis of randomized controlled trials. BMC Health Serv. Res..

[96] Matteucci A., Bonanni M., Sgarra L., Pignalberi C., Aquilani S., Di Fusco S.A., Mariani M.V., Pierucci N., Lavalle C., Fedele S. (2025). Wearable cardioverter defibrillator for transient arrhythmic risk and sudden cardiac death prevention: A systematic review and updated meta-analysis. Open Heart.

[97] Matteucci A., Pignalberi C., Di Fusco S., Aiello A., Aquilani S., Nardi F., Colivicchi F. (2024). Appropriate use of wearable defibrillators with multiparametric evaluation to avoid unnecessary defibrillator implantation. Open Heart.

