# Supplementary material for: Comparative Effectiveness of Non-Pharmacological Interventions for Reducing Heart Failure-Related Unplanned Readmissions: A Systematic Review and Network Meta-Analysis
Source: J Clin Med. 2026 Jul 8;15(14):5344. doi: 10.3390/jcm15145344 (PMC13410378; doi:10.3390/jcm15145344)
Supplement: Supplementary file 1 [file jcm-15-05344-s001.zip › jcm-4365810-supplementary.pdf]

Supplementary Material

Supplementary Table S1. PRISMA Checklist

Section and Topic	Item #	Checklist item	Location where item is reported
**TITLE**			
Title	1	Identify the report as a systematic review.	Title page, Page 1
**ABSTRACT**			
Abstract	2	See the PRISMA 2020 for Abstracts checklist.	Abstract, Page 1
**INTRODUCTION**			
Rationale	3	Describe the rationale for the review in the context of existing knowledge.	Introduction, Pages 1-2
Objectives	4	Provide an explicit statement of the objective(s) or question(s) the review addresses.	Introduction, Page 2
**METHODS**			
Eligibility criteria	5	Specify the inclusion and exclusion criteria for the review and how studies were grouped for the syntheses.	Methods, Page 3
Information sources	6	Specify all databases, registers, websites, organisations, reference lists and other sources searched or consulted to identify studies. Specify the date when each source was last searched or consulted.	Methods, Page 3
Search strategy	7	Present the full search strategies for all databases, registers and websites, including any filters and limits used.	Methods, Page 3, Supplementary Table 2
Selection process	8	Specify the methods used to decide whether a study met the inclusion criteria of the review, including how many reviewers screened each record and each report retrieved, whether they worked independently, and if applicable, details of automation tools used in the process.	Methods, Page 3
Data collection process	9	Specify the methods used to collect data from reports, including how many reviewers collected data from each report, whether they worked independently, any processes for obtaining or confirming data from study investigators, and if applicable, details of automation tools used in the process.	Methods, Page 4
Data items	10a	List and define all outcomes for which data were sought. Specify whether all results that were compatible with each outcome domain in each study were sought (e.g. for all measures, time points, analyses), and if not, the methods used to decide which results to collect.	Methods, Page 4, Supplementary Table 3
	10b	List and define all other variables for which data were sought (e.g. participant and intervention characteristics, funding sources). Describe any assumptions made about any missing or unclear information.	Methods, Page 4, Supplementary Table 3
Study risk of bias assessment	11	Specify the methods used to assess risk of bias in the included studies, including details of the tool(s) used, how many reviewers assessed each study and whether they worked independently, and if applicable, details of automation tools used in the process.	Methods, Page 4
Effect measures	12	Specify for each outcome the effect measure(s) (e.g. risk ratio, mean difference) used in the synthesis or presentation of results.	Methods, Page 4

Section and Topic	Item #	Checklist item	Location where item is reported
Synthesis methods	13a	Describe the processes used to decide which studies were eligible for each synthesis (e.g. tabulating the study intervention characteristics and comparing against the planned groups for each synthesis (item #5)).	Methods, Page 4, Supplementary Table 3
	13b	Describe any methods required to prepare the data for presentation or synthesis, such as handling of missing summary statistics, or data conversions.	Methods, Page 4
	13c	Describe any methods used to tabulate or visually display results of individual studies and syntheses.	Methods, Page 4
	13d	Describe any methods used to synthesize results and provide a rationale for the choice(s). If meta-analysis was performed, describe the model(s), method(s) to identify the presence and extent of statistical heterogeneity, and software package(s) used.	Methods, Page 4
	13e	Describe any methods used to explore possible causes of heterogeneity among study results (e.g. subgroup analysis, meta-regression).	Methods, Page 4-5
	13f	Describe any sensitivity analyses conducted to assess robustness of the synthesized results.	Methods, Page 4
Reporting bias assessment	14	Describe any methods used to assess risk of bias due to missing results in a synthesis (arising from reporting biases).	Methods,Page 4
Certainty assessment	15	Describe any methods used to assess certainty (or confidence) in the body of evidence for an outcome.	Methods, Page 5
**RESULTS**			
Study selection	16a	Describe the results of the search and selection process, from the number of records identified in the search to the number of studies included in the review, ideally using a flow diagram.	Results, Page 5
	16b	Cite studies that might appear to meet the inclusion criteria, but which were excluded, and explain why they were excluded.	Results,Page 5
Study characteristics	17	Cite each included study and present its characteristics.	Results, Page 6
Risk of bias in studies	18	Present assessments of risk of bias for each included study.	Results, Page 11-12
Results of individual studies	19	For all outcomes, present, for each study: (a) summary statistics for each group (where appropriate) and (b) an effect estimate and its precision (e.g. confidence/credible interval), ideally using structured tables or plots.	Results, Page 7-10
Results of syntheses	20a	For each synthesis, briefly summarise the characteristics and risk of bias among contributing studies.	Results, Page 7-12
	20b	Present results of all statistical syntheses conducted. If meta-analysis was done, present for each the summary estimate and its precision (e.g. confidence/credible interval) and measures of statistical heterogeneity. If comparing groups, describe the direction of the effect.	Results, Page 7-10
	20c	Present results of all investigations of possible causes of heterogeneity among study results.	Results, Page 7-10
	20d	Present results of all sensitivity analyses conducted to assess the robustness of the synthesized results.	Results, Page 7-10

Section and Topic	Item #	Checklist item	Location where item is reported
Reporting biases	21	Present assessments of risk of bias due to missing results (arising from reporting biases) for each synthesis assessed.	Results, Page 12
Certainty of evidence	22	Present assessments of certainty (or confidence) in the body of evidence for each outcome assessed.	Results, Page 7-10
**DISCUSSION**			
Discussion	23a	Provide a general interpretation of the results in the context of other evidence.	Discussion, Page 12-14
	23b	Discuss any limitations of the evidence included in the review.	Discussion, Pages 14
	23c	Discuss any limitations of the review processes used.	Discussion, Pages 14
	23d	Discuss implications of the results for practice, policy, and future research.	Discussion, Page 13-14
**OTHER INFORMATION**			
Registration and protocol	24a	Provide registration information for the review, including register name and registration number, or state that the review was not registered.	Methods, Page 2-3
	24b	Indicate where the review protocol can be accessed, or state that a protocol was not prepared.	Methods, Page 2-3
	24c	Describe and explain any amendments to information provided at registration or in the protocol.	Methods, Page 2-3
Support	25	Describe sources of financial or non-financial support for the review, and the role of the funders or sponsors in the review.	Page 15
Competing interests	26	Declare any competing interests of review authors.	Page 15
Availability of data, code and other materials	27	Report which of the following are publicly available and where they can be found: template data collection forms; data extracted from included studies; data used for all analyses; analytic code; any other materials used in the review.	Page 15

Table S2. Search strategy

PubMed/Medline (English database)

Number	Search items	Count
1	(cardiac failure OR heart failure OR heart decompensation[MeSH Terms])[Title/Abstract]	396,771
2	(exercise OR motor activity OR physical activity OR yoga OR Tai Chi OR Qi gong OR mind-body therapies OR cognitive behavioral therapy OR mindfulness OR meditation OR music OR dance OR moxibustion OR Traditional Chinese Medicine OR acupuncture OR diet therapy OR nutrition therapy OR nutrition support OR Micronutrients OR health education OR patient education OR health promotion OR mentoring OR self-management [MeSH Terms]) OR (non-pharmacological intervention OR non-pharmacological therapy OR non-pharmacological management OR non-pharmacological care OR non-pharmacological prevention OR non-drug intervention OR non-drug therapy OR non-drug management OR non-drug care OR non-drug prevention OR traditional chinese exercises OR Baduanjin OR acceptance and commitment OR dialectical behavioral therapy OR interpersonal psychotherapy OR IPT OR Interpersonal therapy OR supportive psychotherapy OR cupping OR Traditional Chinese Medicine External Therapy OR acupoint application OR auricular acupoint OR press needle OR thumb-tack needle OR dietary intervention OR dietary support OR Nutraceutical Supplementation OR nutrition intervention OR Sodium Restriction OR Fluid Restriction OR Dietary Changes OR health coaching OR health consultation OR health counseling OR training OR peer education OR peer support) [Title/Abstract]	4,083,873
3	(Coronary Artery Bypass Grafting OR Percutaneous Coronary Intervention OR cardiac resynchronization therapy OR Implantable Cardioverter Defibrillator OR intra-aortic balloon pump OR Extracorporeal Membrane Oxygenation OR ECMO OR heart transplantation OR heart-assist devices [MeSH Terms]) OR (Medication OR drug OR CABG OR cardiovascular implantable electronic devices OR CIED OR ICD OR insertable cardiac monitor OR ICM OR Cardiac contractility modulation OR CCM OR IABP OR cardiac transplantation OR left ventricular assist device OR LVAD) [Title/Abstract]	2,010,226
4	(rabbit OR mouse OR rat OR monkey OR pig OR cattle OR cow OR sheep OR hedgehog) [Title/Abstract]	2,242,005
5	#1 AND #2 NOT #3 NOT #4	3504

Embase

Number	Search items	Count
1	'heart failure'/exp OR 'cardiac failure':ti,ab,kw OR 'heart failure':ti,ab,kw OR 'heart decompensation':ti,ab,kw	871,467
2	('exercise'/exp OR 'motor activity'/exp OR 'physical activity'/exp OR 'yoga'/exp OR 'tai chi'/exp OR 'qigong'/exp OR 'cognitive behavioral therapy'/exp OR 'mindfulness'/exp OR 'meditation'/exp OR 'music'/exp OR 'dance'/exp OR 'moxibustion'/exp OR 'traditional chinese medicine'/exp OR 'acupuncture'/exp OR 'diet therapy'/exp OR 'nutrition therapy'/exp OR 'micronutrients'/exp OR 'health education'/exp OR 'patient education'/exp OR 'health promotion'/exp OR 'mentoring'/exp OR 'self management'/exp OR 'non-pharmacological intervention' OR 'non-pharmacological therapy' OR 'acceptance'/exp) AND 'commitment'/exp OR 'dialectical behavioral therapy'/exp OR 'interpersonal therapy'/exp OR 'interpersonal psychotherapy'/exp OR 'supportive psychotherapy'/exp OR 'cupping therapy'/exp OR 'dietary intervention'/exp OR 'sodium restriction'/exp OR 'fluid restriction'/exp OR 'health coaching'/exp OR 'training'/exp OR 'peer education'/exp OR 'peer support'/exp OR 'non-pharmacological management':ti,ab,kw OR 'non-pharmacological care':ti,ab,kw OR 'non-pharmacological prevention':ti,ab,kw OR 'non-drug intervention':ti,ab,kw OR 'non-drug therapy':ti,ab,kw OR 'non-drug management':ti,ab,kw OR 'non-drug care':ti,ab,kw OR 'non-drug prevention':ti,ab,kw OR 'traditional chinese exercises':ti,ab,kw OR baduanjin:ti,ab,kw OR 'mind-body therapies':ti,ab,kw OR ipt:ti,ab,kw OR 'traditional chinese medicine external therapy':ti,ab,kw OR 'acupoint application':ti,ab,kw OR 'auricular acupoint':ti,ab,kw OR 'press needle':ti,ab,kw OR 'thumb-tack needle':ti,ab,kw OR 'dietary support':ti,ab,kw OR 'nutraceutical supplementation':ti,ab,kw OR 'nutrition intervention':ti,ab,kw OR 'dietary changes':ti,ab,kw OR 'health consultation':ti,ab,kw OR 'health counseling':ti,ab,kw OR 'nutrition support':ti,ab,kw	673,008
3	'coronary artery bypass grafting'/exp OR 'percutaneous coronary intervention'/exp OR 'cardiac resynchronization therapy'/exp OR 'implantable cardioverter defibrillator'/exp OR 'intra-aortic balloon pump'/exp OR 'extracorporeal membrane oxygenation'/exp OR ecmo OR 'heart transplantation'/exp OR 'heart-assist devices'/exp OR medication:ti,ab,kw OR drug:ti,ab,kw OR cabg:ti,ab,kw OR 'cardiovascular implantable electronic devices':ti,ab,kw OR cied:ti,ab,kw OR icd:ti,ab,kw OR 'insertable cardiac monitor':ti,ab,kw OR icm:ti,ab,kw OR 'cardiac contractility modulation':ti,ab,kw OR ccm:ti,ab,kw OR iabp:ti,ab,kw OR 'cardiac transplantation':ti,ab,kw OR 'left ventricular assist device':ti,ab,kw OR lvad:ti,ab,kw	3,383,680
4	rabbit:ab,ti OR mouse:ab,ti OR rat:ab,ti OR monkey:ab,ti OR pig:ab,ti OR cattle:ab,ti OR cow:ab,ti OR sheep:ab,ti OR hedgehog:ab,ti	2,691,597
5	#1 AND #2 NOT #3 NOT #4	1200

Web of Science Core Collection

Number	Search items	Count

1	TI (cardiac failure OR heart failure OR heart decompensation)	54244
2	TS (exercise OR motor activity OR physical activity OR yoga OR Tai Chi OR Qi gong OR mind-body therapies OR cognitive behavioral therapy OR mindfulness OR meditation OR music OR dance OR moxibustion OR Traditional Chinese Medicine OR acupuncture OR diet therapy OR nutrition therapy OR nutrition support OR Micronutrients OR health education OR patient education OR health promotion OR mentoring OR self-management OR non-pharmacological intervention OR non-pharmacological therapy OR non-pharmacological management OR non-pharmacological care OR non-pharmacological prevention OR non-drug intervention OR non-drug therapy OR non-drug management OR non-drug care OR non-drug prevention OR traditional chinese exercises OR Baduanjin OR acceptance and commitment OR dialectical behavioral therapy OR interpersonal psychotherapy OR IPT OR Interpersonal therapy OR supportive psychotherapy OR cupping OR Traditional Chinese Medicine External Therapy OR acupoint application OR auricular acupoint OR press needle OR thumb-tack needle OR dietary intervention OR dietary support OR Nutraceutical Supplementation OR nutrition intervention OR Sodium Restriction OR Fluid Restriction OR Dietary Changes OR health coaching OR health consultation OR health counseling OR training OR peer education OR peer support)	2266389
3	TI (Coronary Artery Bypass Grafting OR Percutaneous Coronary Intervention OR cardiac resynchronization therapy OR Implantable Cardioverter Defibrillator OR intra-aortic balloon pump OR Extracorporeal Membrane Oxygenation OR ECMO OR heart transplantation OR heart-assist devices OR Medication OR drug OR CABG OR cardiovascular implantable electronic devices OR CIED OR ICD OR insertable cardiac monitor OR ICM OR Cardiac contractility modulation OR CCM OR IABP OR cardiac transplantation OR left ventricular assist device OR LVAD)	326137
4	TI (rabbit OR mouse OR rat OR monkey OR pig OR cattle OR cow OR sheep OR hedgehog)	955837
5	#1 AND #2 NOT #3 NOT #4	9094

Cochrane Central Library

Number	Search items	Count
#1	MeSH descriptor: [Heart Failure] this term only	14660

#2	(heart failure OR heart decompensation OR cardiac dysfunction):ti,ab,kw	54059
#3	#1 OR #2	54059
#4	MeSH descriptor: [Exercise] this term only	26399
#5	MeSH descriptor: [Motor Activity] this term only	4271
#6	(physical activity):ti,ab,kw	65252
#7	MeSH descriptor: [Yoga] this term only	1272
#8	MeSH descriptor: [Tai Ji] this term only	608
#9	MeSH descriptor: [Qigong] this term only	182
#10	MeSH descriptor: [Mind-Body Therapies] this term only	227
#11	MeSH descriptor: [Cognitive Behavioral Therapy] this term only	12300
#12	MeSH descriptor: [Mindfulness] this term only	2557
#13	MeSH descriptor: [Meditation] this term only	1119
#14	MeSH descriptor: [Music Therapy] this term only	1414
#15	MeSH descriptor: [Dancing] this term only	312
#16	MeSH descriptor: [Moxibustion] this term only	698
#17	MeSH descriptor: [Medicine, Chinese Traditional] this term only	1530
#18	MeSH descriptor: [Acupuncture] this term only	216
#19	MeSH descriptor: [Diet Therapy] this term only	479
#20	MeSH descriptor: [Nutrition Therapy] this term only	317
#21	(nutrition support):ti,ab,kw	5841
#22	MeSH descriptor: [Micronutrients] this term only	1274
#23	(health education):ti,ab,kw	57136
#24	MeSH descriptor: [Patient Education as Topic] this term only	11013
#25	MeSH descriptor: [Health Promotion] this term only	8093
#26	(mentoring):ti,ab,kw	1549
#27	MeSH descriptor: [Self-Management] this term only	1455
#28	(non-pharmacological intervention OR non-pharmacological therapy OR non-pharmacological management OR non-pharmacological care OR non-pharmacological prevention OR non-drug intervention OR non-drug therapy OR non-drug management OR non-drug care OR non-drug prevention OR traditional chinese exercises OR Baduanjin OR acceptance and commitment OR dialectical behavioral therapy OR interpersonal psychotherapy OR IPT OR Interpersonal therapy OR supportive psychotherapy OR cupping OR Traditional Chinese Medicine External Therapy OR acupoint application OR auricular acupoint OR press needle OR thumb-tack needle OR dietary intervention OR dietary support OR Nutraceutical Supplementation OR nutrition intervention OR Sodium Restriction OR Fluid Restriction OR Dietary Changes OR health coaching OR health consultation OR health counseling OR training OR peer education OR peer support):ti,ab,kw	237860
#29	#4 OR #5 OR #6 OR #7 OR #8 OR #9 OR #10 OR #11 OR #12 OR #13 OR #14 OR #15 OR #16 OR #17 OR #18 OR #19 OR #20 OR #21 OR #22 OR #23 OR #24 OR #25 OR #26 OR #27 OR #28	337054
#30	MeSH descriptor: [Coronary Artery Bypass] this term only	6395
#31	MeSH descriptor: [Percutaneous Coronary Intervention] this term only	4484
#32	MeSH descriptor: [Cardiac Resynchronization Therapy] this term only	617

#33	MeSH descriptor: [Defibrillators, Implantable] this term only	1502
#34	MeSH descriptor: [Intra-Aortic Balloon Pumping] this term only	229
#35	MeSH descriptor: [Extracorporeal Membrane Oxygenation] this term only	386
#36	MeSH descriptor: [Heart Transplantation] this term only	854
#37	MeSH descriptor: [Heart-Assist Devices] this term only	411
#38	(Medication OR drug OR CABG OR cardiovascular implantable electronic devices OR CIED OR ICD OR insertable cardiac monitor OR ICM OR Cardiac contractility modulation OR CCM OR IABP OR cardiac transplantation OR left ventricular assist device OR LVAD):ti,ab,kw	815421
#39	#30 OR #31 OR #32 OR #33 OR #34 OR #35 OR #36 OR #37 OR #38	820063
#40	(rabbit OR mouse OR rat OR monkey OR pig OR cattle OR cow OR sheep OR hedgehog):ti,ab,kw	14969
#41	#3 AND #29 NOT #39 NOT #40	4218

CINAHL

Number	Search items	Count
1	SU (cardiac failure OR heart failure OR heart decompensation OR cardiac dysfunction)	707
2	SU (non-pharmacological intervention OR non-pharmacological therapy OR non-pharmacological management OR non-pharmacological care OR non-pharmacological prevention OR non-drug	13476

	intervention OR non-drug therapy OR non-drug management OR non-drug care OR non-drug prevention OR exercise OR motor activity OR physical activity OR yoga OR Tai Chi OR Qi gong OR traditional chinese exercises OR Baduanjin OR mind-body therapies OR cognitive behavioral therapy OR mindfulness OR meditation OR acceptance and commitment OR dialectical behavioral therapy OR music OR dance OR interpersonal psychotherapy OR IPT OR Interpersonal therapy OR moxibustion OR supportive psychotherapy OR Traditional Chinese Medicine Techniques OR moxibustion OR acupuncture OR cupping OR Traditional Chinese Medicine External Therapy OR acupoint application OR auricular acupoint OR press needle OR thumb-tack needle OR dietary intervention OR dietary therapy OR dietary support OR Nutraceutical Supplementation OR nutrition intervention OR nutrition therapy OR nutrition support OR Sodium Restriction OR Fluid Restriction OR Dietary Changes OR Micronutrient OR health education OR patient education OR health coaching OR health consultation OR health counseling OR health promotion OR training OR behavior change OR self-management OR peer education OR peer support)	
3	SU (Coronary Artery Bypass Grafting OR CABG Percutaneous Coronary Intervention OR PCI OR cardiovascular implantable electronic devices OR CIED OR cardiac resynchronization therapy OR CRT OR Implantable Cardioverter Defibrillator OR ICD OR insertable cardiac monitor OR ICM Cardiac contractility modulation OR CCM OR intra-aortic balloon pump OR IABP OR Extracorporeal Membrane Oxygenation OR ECMO OR heart transplantation OR cardiac transplantation OR left ventricular assist device OR LVAD)	118
4	SU (rabbit OR mouse OR rat OR monkey OR pig OR cattle OR cow OR sheep OR hedgedog)	62
5	#1 AND #2 NOT #3 NOT #4	160

APA PsyINFO/ APA PsycArticles/ Psychology and Behavioral Sciences Collection

Number	Search items	Count
1	SU (cardiac failure OR heart failure OR heart decompensation OR cardiac dysfunction)	725
2	SU (non-pharmacological intervention OR non-pharmacological therapy OR non-pharmacological management OR non-pharmacological care OR non-pharmacological prevention OR non-drug intervention OR non-drug therapy OR non-drug management OR non-drug care OR non-drug prevention OR exercise OR motor activity OR physical activity OR yoga OR Tai Chi OR Qi gong OR traditional chinese	71870

	exercises OR Baduanjin OR mind-body therapies OR cognitive behavioral therapy OR mindfulness OR meditation OR acceptance and commitment OR dialectical behavioral therapy OR music OR dance OR interpersonal psychotherapy OR IPT OR Interpersonal therapy OR moxibustion OR supportive psychotherapy OR Traditional Chinese Medicine Techniques OR moxibustion OR acupuncture OR cupping OR Traditional Chinese Medicine External Therapy OR acupoint application OR auricular acupoint OR press needle OR thumb-tack needle OR dietary intervention OR dietary therapy OR dietary support OR Nutraceutical Supplementation OR nutrition intervention OR nutrition therapy OR nutrition support OR Sodium Restriction OR Fluid Restriction OR Dietary Changes OR Micronutrient OR health education OR patient education OR health coaching OR health consultation OR health counseling OR health promotion OR training OR behavior change OR self-management OR peer education OR peer support)	
3	SU (Coronary Artery Bypass Grafting OR CABG Percutaneous Coronary Intervention OR PCI OR cardiovascular implantable electronic devices OR CIED OR cardiac resynchronization therapy OR CRT OR Implantable Cardioverter Defibrillator OR ICD OR insertable cardiac monitor OR ICM Cardiac contractility modulation OR CCM OR intra-aortic balloon pump OR IABP OR Extracorporeal Membrane Oxygenation OR ECMO OR heart transplantation OR cardiac transplantation OR left ventricular assist device OR LVAD)	252
4	SU (rabbit OR mouse OR rat OR monkey OR pig OR cattle OR cow OR sheep OR hedgedog)	11277
5	#1 AND #2 NOT #3 NOT #4	37

Table S3. Characteristics of included studies

Study/Country	Participants (EG/CG)					Intervention		Duration/Follow-up (Attrition rate)	Outcomes/Measures	Findings
	Sample size	Mean age	LVEF (%)	NYHA (%)	Comorbidities (%)	EG	CG			
1.Chen et al. (2018)/Taiwan	19/18	61±11/60±16	36±9/32±11	Not reported	ICM(31.6/16.7), RHD(0/5.6), DCM(68.4/83.3)	**Content:** individualized cardiac rehabilitation program (including home-based cardiac rehabilitation exercise, dietary education, and daily activity management)<br>**No./length/frequency of session:** at least 3 sessions per week/30 minutes per session<br>**Format/delivery mode:** individualized; developed based on CPET and 6MWT results; telephone follow-up once every 2 weeks<br>**Setting:** home	Standard care and maintaining baseline activity levels	3 months/3 months (45.7%/55.0%)	VO_2 peak; 6MWD; AT; MLHFQ score; HF-related readmission rate; Hemodynamic parameters (cardiac output, stroke volume, left ventricular ejection fraction, thoracic fluid index, etc.)	VO_2 peak, 6MWD, AT, MLHFQ score, and HF-related readmission rate ↑ at 3 months. No significant differences were found in hemodynamic parameters.
2.Davidson et al. (2010)/Australia	53/52	71.6/73.9	Not reported	Class I(3.8/0), Class II(37.7/32.7),Class III(60.4/67.3)	COPD(11.0/13.0), DM(26.4/26.9), PVD(28.3/32.7), AF(47.1/46.1), Previous AMI(62.3/55.8), OSA(11.3/7.7), Stroke(5.7/11.5), Arthritis(60.4/53.9), Hypertension(79.3/69.2),	**Content:** Individualized exercise program (including endurance training and resistance training) + multidisciplinary education (self-management, symptom monitoring, medication adherence) + home-based exercise guidance<br>**No./length/frequency of session:**12-week program; 1 session of hospital-based gym training per week (30 minutes of endurance training + resistance training); monthly telephone follow-up<br>**Format/delivery mode:** Group education + individual exercise guidance; combined with home-based exercise plan (no ECG monitoring; intermittent monitoring of heart rate, blood pressure, and blood oxygen saturation)<br>**Setting:** Hospital outpatient clinic	Standard care	3 months/ 3 months, 12 months (0%)	All-cause readmission rate; HF-related readmission rate; Survival rate; MLHFQ; 6MWD; NYHA classification; HFNAQ scale	All-cause readmission rate, HF-related readmission rate, and survival rate ↑ at 12 months. MLHFQ and HFNAQ scores↑ at 3 months. No significant differences were found in MLWHFQ and HFNAQ scores at 12 months.

3.Corvera-Tindel et al. (2003)/USA	42/37	63.8±10.1/ 61.3±11.1	29.1±8.5/24.7±8.8	Class II(76.2/83.8), Class III-IV(21.4/16.2)	Arthritis(42.9/32.4), Hypertension(76.2/73.0), DM(40.5/29.7)	**Content:** Progressive home walking exercise (intensity: 40%–65% maximal heart rate; duration: 10–60 minutes)<br>**No./length/frequency of session:** 5 days/week, once daily, progressive increase from 10 to 60 minutes<br>**Format/delivery mode:** Individual; pedometer for tracking, weekly nurse home visits (first 6 weeks) and biweekly visits (last 6 weeks)<br>**Setting:** home	Usual activity (maintain normal daily activities without regular exercise program)	3 month/3 month (11.9%/13.5%)	VO_2 peak, 6MWD, HFFSI, DFI, PGRS, HF-related readmission rate(3month).	6MWD and PGRS ↑ at 3 month. No significant differences were found in VO_2 peak, HFFSI, and DFI at 3 month.
4.Dracup et al. (2007)/USA	86/87	53.3±12.7/ 54.6±12.5	26.7±6.7/26.1±7.0	Class II(32.3/21.8),Class III(59.3/66.7), Class IV(8.1/11.5)	CAD(53.5/34.9), Hypertension(45.3/45.3), DM(31.4/19.8)	**Content:** Graduated low-level exercise protocol (aerobic training: 10–45 min at 40%–60% maximal heart rate; resistive training: 2 sets of 10 repetitions at 80% of one repetition maximum for upper/lower extremities)<br>**No./length/frequency of session:** Aerobic exercise 4 times weekly; resistive training 3 days/week (on non-walking days)<br>**Format/delivery mode:** Home-based; self-administered with initial research nurse guidance; pedometer and daily log for compliance monitoring<br>**Setting:** home	Usual care (maintained usual daily activities without structured exercise; monthly home visits by research nurses for attention control)	12 months / Baseline-3 months-6 months-12 months (1.15%)	All-cause hospitalizations, HF-related readmission rate(12month), emergency department admissions, urgent transplantation,death rate , VO_2 peak, Anaerobic threshold; 6MWD, MWHFQ scale, Multiple Affect Adjective Checklist for anxiety, depression, hostility.	No significant differences were found in all-cause hospitalizations, HF-related readmission rate(12month); emergency department admissions, urgent transplantation, and death rate at 12 month. No significant differences were found in VO_2 peak, anaerobic threshold, 6MWD, MLWHFQ scale, and Multiple Affect Adjective Checklist for anxiety, depression, hostility at

										baseline, 3 months, 6 months and 12 months. Hospitalizations per patient and total no. of hospitalizations in patients having ≥2 readmissions,↑ at 12 months.
5.Willenheimer et al. (2001)/Sweden	17/20	64±5/64±8	35±12/ 38±10	2.1±0.7/ 2.4±0.7	IHD(85.0/78.0)	**Content:** Bicycle ergometer interval training at 80% of peak VO (or Borg scale 15 for atrial fibrillation) **No./length/frequency of session:** Gradually increased from 15 min twice/week to 45 min three times/week (total 16 weeks) **Format/delivery mode:** Group-based, supervised by physiotherapist **Setting:** hospital	Usual activity	4-month /6-month follow-up (24.5%)	PGACQoL, DFI, Habitual physical activity score, HF-related readmission rate.	No significant differences.
6.Hambrecht et al. (1995)/Germany	12/10	50±12/52±8	26±9/27±10	Class II (50/60), Class III (50/40)	Not reported	**Content:**Outpatient exercise training (including in-hospital supervised training and home-based training, combined with bicycle ergometer exercise, walking, gymnastics, and ball games) **No./length/frequency of session:** Initial 3-week in-hospital training: 6 sessions per day, 10 minutes per session, intensity at 70% of peak oxygen consumption (VO_2peak); Post-discharge home-based training: 2 sessions per day, total ≥40 minutes; at least 2 sessions of 60-minute group training per week; target heart rate corresponding to 70% of VO_2peak **Format/delivery mode:** Individualized supervision (in-hospital) + home-based self-administered training (combined with ECG heart rate monitoring) + group training **Setting:** Hospital (first 3 weeks) + home + group training venue	Usual care	6months/Baseline-6 months (25%/10%)	VO_2peak, VO_2 at ventilatory threshold, Exercise duration, Mitochondrial volume density of the quadriceps, Cardiac output, Lower limb blood flow and oxygen consumption; Heart rate; Blood pressure, Femoral venous lactate concentration, NYHA classification, HF-related readmission rate(6month)	VO_2peak, VO_2 at ventilatory threshold, Exercise duration, Mitochondrial volume density of the quadriceps, Cardiac output, and NYHA classification ↑ at 6 months.
7.Mueller et al.	25/25	55.0±10	<40	Not	Not reported	**Content:** Educational sessions, low-fat diet, and	Usual	1month/6.2±1.4	peak VO_2, exercise	peak VO_2 ↑ at 1

(2007)/Switzerland				reported		individualized exercise prescription (2 hours of exercise daily)<br>**No./length/frequency of session:** 1-month intervention period; 2 hours of exercise daily (indoor cycling 5 times/week, 30 minutes per session; outdoor walking twice daily, 45 minutes per session)<br>**Format/delivery mode:** Individualized exercise prescription combined with educational sessions and dietary guidance<br>**Setting:** Residential rehabilitation centre	care	years (40%/56%)	time, peak watts, energy expenditure from recreational activities, kcal/week, all-cause mortality, HF-related readmission rate	months. No significant differences were found in peak VO_2, kcal/week. Energy expenditure from recreational activities ↑ at 6 years. All-cause mortality and HF-related readmission rate↑ at 6 years.
8.Giannuzzi et al. (2003)/Italy	45/45	60±7/61±7	25/25	Class II (62/73), Class III (38/27)	Hypertension(66.0/60.0)	**Content**: Moderate-intensity exercise training (including supervised bicycle ergometer training, home-based brisk walking, and gymnastics)<br>**No./length/frequency of session:** 3–5 sessions per week, 30 minutes per session (gradually increasing intensity and duration initially); Home-based training: Brisk walking for ≥30 minutes daily + intermittent gymnastics (30 minutes per session)<br>**Format/delivery mode:** Supervised group training + individual home-based training; combined with exercise diary documentation<br>**Setting:** Cardiac rehabilitation center (supervised training) + home (unsupervised training)	Usual care	6months/Baseline-6 months (0%)	EDV, ESV, LVEF, VO_2peak, Exercise duration; Work capacity, 6MWD, MWHFQ scale, HF-related readmission rate(6month)	EDV, ESV, LVEF, VO_2peak, Exercise duration, Work capacity, 6MWD, MWHFQ scale ↑ at 6 months. HF-related readmission rate↑ at 6months.
9.Tsuchihashi-Makaya et al. (2013)/Japan	79/82	76.9±10.9/ 75.8±12.1	47.4±16.6/47.4±15.7	Class I(10.1/17.1),Class II(84.8/76.8),Class III(5.1/6.1)	Hypertension (51.9/50.0), DM(25.3/22.0), Hyperuricemia (44.3/42.7), previous MI(21.5/18.3), stroke (17.7/14.6), COPD (10.1/4.9), AF (43.0/62.2)	**Content:**Symptom monitoring, weight monitoring, patient education (including HF pathophysiology, treatment, diet, activity, self-management, etc.), psychological counseling, medication optimization, multidisciplinary team support (cardiologist, dietitian, pharmacist, social worker)<br>**No./length/frequency of session:** First home visit within 14 days after discharge; home visits every 2 weeks for the first 2 months; monthly telephone follow-up thereafter until 6 months<br>**Format/delivery mode:** Nurse-led; home visits (face-to-face) + telephone follow-up<br>**Setting:** home	Allied health and follow up services	1 year / Baseline-2 months-6 months-12 months (5.9%/2.4%)	HADS, SF-8, all-cause mortality, HF-related readmission rate	HADS score, and SF-8 score ↑ at 2,6 and 12 months. HF-related readmission rate↑ at 2,6 and 12 months. No significant differences were found in all-cause mortality.

10.Chen et al. (2017)/China	31/31	61.1±14.2/ 62.4±14.9	39.9/47 .1	Class II (3.2/3.2) , Class III (48.4/51. 6), Class IV (48.4/45. 2)	Hypertension (48.4/54.8), DM(19.4/25.8), previous MI(19.4/12.9), stroke (9.7/6.5), Renal dysfunction (12.9/6.5), AF (38.7/35.3), Anemia(29.0/4 1.9)	**Content:** Discharge education (HF definition, symptoms, weight control, medication adherence, diet), physical training (individualized based on SPPB scores: respiratory exercises, muscle strengthening, walking, stair stepping), follow-up visits, telephone calls, home visits, depression management (psychiatrist consultation if PHQ-9 ≥10)<br>**No./length/frequency of session:** Home visit at 2 weeks post-discharge; telephone calls every 2 weeks; intensified education at 90 and 180 days; physical training 3 sessions/week (progressing from 20 to 40 min/session)<br>**Format/delivery mode:**Multidisciplinary team (cardiologists, nurses, dietitian, psychiatrist); individual; face-to-face (discharge education, home visits, clinic follow-up), telephone calls<br>**Setting:** Hospital, home, outpatient clinic	Usual care	6 month/Baselin e-3 month- 6 month (0%/6.5%)	MWHFQ scale, SPPB scale, PHQ-9, EHFScBS, Mortality or HF-related readmission rate	MWHFQ scale, PHQ-9, and EHFScBS↑ at 6 months.<br>No significant differences were found in SPPB scale, mortality and HF-related readmission rate
11. Blue et al. (2001)/UK	84/81	74.4±8.6/7 5.6±7.9	Not report ed	Class II (23.0/20. 0), Class III (34.0/42. 0), Class IV (43.0/38. 0)	Hypertension (43.0/52.0), DM(18.0/19.0), previous MI(55.0/51.0), Chronic lung disease(27.0/22 .0), AF (35.0/30.0), Angina(29.0/41 .9), Valve disease(18.0/15 .0)	**Content:** Patient education (heart failure [HF] knowledge and treatment, dietary guidance, exercise advice), medication optimization (adjustment of ACEIs, diuretics, and digoxin), electrolyte monitoring, self-management skills (early identification of decompensation symptoms), psychological support, and multidisciplinary collaboration<br>**No./length/frequency of session:** Planned home visits (decreasing frequency) and telephone follow-up as needed for 1 year<br>**Format/delivery mode:** Individualized; home visits + telephone follow-up<br>**Setting:** home	Usual care	12 months/12 months (2.4%/ 0%)	All-cause mortality, HF-related readmission(12-momth), total number of admissions, HF-related hospital days	HF-related readmission, total number of admissions, and HF-related hospital days ↑ at 12 months.<br>No significant differences were found in all-cause mortality.
12. Leventhal et al. (2011)/Switzerlan d	22/20	76.7±7.1/7 7.6±6.0	45.0 (30.0~6 0.0)/42. 0(28.0~ 57.5)	Class I(16.7/1 5.0), Class II (35.0/56. 3), Class	DM(31.8/20.0)	**Content:**Self-care education (heart failure [HF] knowledge, symptom recognition, medication management, diet/weight monitoring), individualized goal-setting, psychosocial support; accompanied by the Swiss Heart Foundation toolkit<br>**No./length/frequency of session:**1 home visit	Usual Care	12 months/12 months (27.3%/30.0%)	All-cause mortality, All-cause hospital readmission, HF-related hospital	EQ-5D ↑ at 12 months. No significant differences were found in all-cause mortality,

				III (50.0/25.0)		(approximately 1 week post-intervention) + 17 telephone follow-ups (decreasing frequency: once weekly for the first 4 weeks, once every 2 weeks for the next 4 weeks, and once monthly for the subsequent 6 months)<br>**Format/delivery mode:** Home visits (face-to-face) + telephone follow-up; individualized care plan<br>**Setting:** Outpatient clinic/home			readmission(12month), Cardiac non-HF hospital readmission, Quality of life (EQ-5D, MLHF Questionnaire)	all-cause hospital readmission, HF-related hospital readmission, Cardiac non-HF hospital readmission, MLHF Questionnaire.
13. Kalter-Leibovici et al. (2017)/Israel	682/678	70.8±11.6/70.7±11.0	Preserved(>50):(20.2/16.6), Reduced(<50): (79.8/83.4)	Class I (0.7/0.6), Class II(11.9/17.1), Class III(79.9/78.0), Class IV(7.5/4.3)	Hypertension (74.2/74.7), DM(52.2/50.0), COPD (18.0/15.5), Chronic lung disease(27.0/22.0), Chronic AF (23.4/26.9), Renal failure (56.5/56.8)	**Content:** Multidisciplinary team-coordinated care, patient education, symptom monitoring, medication adherence management, medication titration, home-based remote monitoring (weight, blood pressure, heart rate)<br>**No./length/frequency of session:**Remote nurse contacts (27.3±17.8 times in the first year; 18.8±13.7 times per year overall); follow-up at the cardiac center ≥once every 6 months<br>**Format/delivery mode:** Multidisciplinary team (nurses, cardiologists, dietitians, social workers); remote monitoring (Medic4All® device); telephone/video communication.<br>**Setting:** Community cardiac center, home.	Standard health care	2.7 years (range: 0-5.0 years)/2.7 years (range: 0-5.0 years) (36.%/39.8%)	Time to first heart failure (HF) hospitalization, all-cause mortality , Total number of hospitalizations,number of HF-related hospitalizations, all-cause mortality, SF-36,PHQ-9, NYHA classification, 6MWT, HF-related readmission rate	SF-36, PHQ-9 ↑. No significant differences were found in all-cause mortality , Total number of hospitalizations, number of HF-related hospitalizations, all-cause mortality, HF-related readmission rate.
14.Del Sindaco et al. (2007)/Italy	86/87	77.4±5.9/77.5±5.7	33.5±11/32.5±10	Class II (37.2/39.1), Class III (51.2/56.3) Class IV (11.6/4.6)	Hypertension (67.4/62.1), DM(32.6/31.0), COPD (35.9/33.3), previous MI(51.2/55.1)	**Content:**Discharge planning, patient education (low-sodium diet, medication therapy, self-monitoring of blood pressure/symptoms, weight management, etc.), treatment optimization, improved physician-patient communication, early symptom recognition, flexible diuretic adjustment plan<br>**No./length/frequency of session:**<br>Hospital follow-up: Initiated within 14 days after discharge, followed by visits at 1 month, 3 months, and every 6 months (mean 4.9 times per patient);<br>Nurse telephone follow-up: Mean 8.5 times per patient (total 731 sessions, ~15 minutes per session)<br>**Format/delivery mode:** Multidisciplinary collaboration (cardiologists + nurse coordinators +	Usual Care	2 years/Baseline to 2 years (6.5%/5.6%)	All-cause death, HF-related readmission rate(12-momth), All-cause hospital admissions, Heart failure hospital admissions Length of hospital stay, NYHA class, MLWHF questionnaire, b-blocker prescription rate,	Length of hospital stay, NYHA class, MLWHF questionnaire, and cost-effectiveness ↑ at 2 years. All-cause death, heart failure hospital admissions, All-cause hospital admissions,

						general practitioners); combined hospital outpatient follow-up, home visits, and telephone follow-up **Setting:** Hospital heart failure clinic + home			Cost-effectiveness	HF-related readmission rate ↑ at 2 years.
15.Angermann et al. (2012)/Germany	352/363	67.7±12.8/ 69.4±11.5	30.0±8.0/30.0±8.0	Class I (3.0/2.0), Class II(54.0/62), Class III(40.0/31.0), Class IV(3.0/5.0)	Hypertension (72.0/77.0), DM(36.0/36.0), COPD (18.0/21.0), CAD(55.0/61.0), AF (32.0/26.0), Renal dysfunction(42.0/41.0), Anemia(31.0/32.0)	**Content:**Nurse-coordinated multidisciplinary disease management program (HeartNetCare-HF), including telephone monitoring (19-item standardized questionnaire), patient education (medication management, symptom recognition, self-care), healthcare collaboration (communication with general practitioners and cardiologists), caregiver training, and consultation for non-cardiac issues (e.g., digestive, musculoskeletal diseases). **No./length/frequency of session:** Once weekly for the first month, then adjusted based on NYHA class (every 2 weeks for NYHA III-IV, once monthly for NYHA I-II); 10-15 minutes per session. **Format/delivery mode:**Telephone-based, combined with face-to-face training during hospitalization; using standardized modules (e.g., START monitoring module, education module). **Setting:** Hospital-led call center, patients' homes	Usual care	6 months/6 months (12.8%/6.0%)	Composite of all-cause death or readmissions, All-cause mortality, cardiovascular mortality, HF-related readmission rate(6-momth), NYHA class, SF-36, LVEF, medication adherence	All-cause death ↑ at 6 months. NYHA class and SF-36 ↑ at 6 months. No significant differences were found in all-cause readmissions, , HF-related readmission rate, and medication adherence.
16. Riegel et al. (2006)/USA	69/65	71.6±10.8/ 72.7±11.2	42.3±18.3/44.1±18.1	Class II (17.4/20.0),Class III (44.9/47.7), Class IV(37.7 / 32.3)	Hypertension (84.1/73.8); COPD(21.7/33.8); History of MI(26.1/29.2); Diabetes(55.1/63.1)%;Diabetes with end-organ damage (17.4/18.5);Renal disease with creatinine >3 mg(8.7/4.6)	**Content:** Standardized telephone case management including education on self-care skills (medication/diet adherence, symptom monitoring), cultural adaptation (personalismo, family inclusion, concrete problem-solving), and coordination with healthcare providers. **No./length/frequency of session:** Average 13.5 patient contacts (SD 5.9) + 8.4 family contacts (SD 6.3) over 6 months; most calls in the first month. **Format/delivery mode:** Telephone calls by bilingual/bicultural Mexican-American registered nurses; educational materials in Spanish/English. **Setting:** Community-based (post-hospital discharge).	Usual care	6 months /Baseline, 1 month, 3 months, 6 months (17.4%/15.4%)	HF hospitalizations, All-cause hospitalizations, HF-related readmission rate(3month), HF hospital days, All-cause hospital days, HF inpatient costs, All-cause inpatient costs, MLHFQ, EQ-5D, PHQ-9, Mortality	No significant differences.

17. Piamjariyakul et al. (2015)/USA	10/10	65.1±8.0/57.3±10.9	Not reported	Not reported	The overall distribution of IG and EG(55), MI or cardiovascular disease (20), DM (20), osteoarthritis/pain (20), depression(5); thyroid problems, asthma(5), and HIV(5).There was no statistically significant difference in the baseline between the two groups	**Content:** Participation in a home-based HF care plan (e.g., medication adherence, fluid and sodium restriction). Development of daily HF care skills. Management of caregiver stress and burden, and facilitation of seeking professional help. Emergency preparedness and end-of-life discussions**.**<br>**No./length/frequency of session:** 4-week telephone coaching, 1 session per week, 60-90 minutes per session.<br>**Format/delivery mode:** Telephone coaching combined with educational materials (AHA guidelines, low-sodium diet handbook, pill organizers, etc.).<br>**Setting:** home	Usual care	4 weeks/6 months. (20%/10%)	HF-related readmission rate (6month), confidence, social support, depression, preparedness, burden.	HF-related readmission rate and depression ↑ at 6 months. Confidence, and social support ↑ at 6 months. No significant differences were found in preparedness and burden.
18. Dunagan et al. (2005)/USA	76/75	70.5±12.7/69.4±13.9	<25:(38/48);25-40% (36/31);41-50:(8/7); >50(18/15)	Class II (22.0/17.0);Class III (71.0/72.0);Class IV (7.0/11.0)	There was no significant difference in baseline comorbidities between the intervention and control groups. The median Charlson Comorbidity Index score was 3 in both groups (range: intervention group 1–8, control group 1–9). Over 90% of patients had at least one	**Content:** Nurses provided telephone-based self-management education (covering HF etiology, treatment principles, and symptom monitoring), guideline-based medication adherence promotion, HF deterioration screening (using standardized tools), and diuretic adjustment or physician contact for acute exacerbations.<br>**No./length/frequency of session:** Initial call within 3 days post-discharge, followed by at least one call per week for the next 2 weeks. Subsequent call frequency was adjusted based on the patient's clinical status and self-management capability (no fixed session duration recorded).<br>**Format/delivery mode:** Telephone follow-up; supplemented with home visits for a subset of patients (n=20), and provision of weight scales to 18 patients.<br>**Setting:**Hospital-led telephone follow-up, combined with home visits for selected patients.	Usual care	1 year/ 6 months, 12 months (0%)	Time to first hospitalization event, all-cause mortality, number of hospitalizations and associated costs, NYHA class, SF-12 scale, care satisfaction. HF-related readmission rate(6month), all-cause readmission rate, and hospital length of stay.	Time to first hospitalization event, all-cause readmission rate and HF-related readmission rate ↓. Number of hospitalizations, associated costs, and hospital length of stay ↑ at 6 months. No significant differences were found in number of hospitalizations, associated costs, and hospital length of stay at 12 months. No

					serious comorbidity, and 31% of patients exhibited depressive symptoms.					significant differences were found in all-cause mortality, SF-12, NYHA class, and care satisfaction.
19. Jiang et al. (2021)/Singapore A	49/56	69.1±10.5/ 68.8±13.1	Not reported	NYHA I-II(32.7/30.4); NYHA III-IV(67.3/69.6)	CHD(55.1/50.9), Hypertension (65.3/91.1), T2DM (59.2/60.7)	**Content:** home-based self-management intervention (HF self-care knowledge and skills, motivational interviewing, self-management toolkit, and home visit)<br>**No./length/frequency of session:** three 40-min~1 h biweekly home visits.<br>**Format/delivery mode:** individual; face to face<br>**Setting:** home	Allied health and follow up services	6weeks/Baseline-1.5-3-6 months (26.6%)	QOL/MLHFQ. Anxiety/HADS. HF-related readmission rate, Cardiac-related emergency department visits.	QOL at 1.5 and 3 months ↑. No significant differences were found in anxiety, HF-related readmission rate, cardiac-related emergency department visits.
19.Jiang et al. (2021)/Singapore B	57/56	66.8±11.8/ 68.8±13.1	Not reported	NYHA I-II(28.1/30.4); NYHA III-IV(71.9/69.6)	CHD(64.9/50.9), Hypertension (65.3/91.1), T2DM (52.6/60.7)	**Content:** home-based self-management intervention (HF self-care knowledge and skills, motivational interviewing, self-management toolkit, home visit, and mhealth)<br>**No./length/frequency of session:** three 40-min~1 h biweekly home visits.<br>**Format/delivery mode:** individual; face to face and smartphone application<br>**Setting:** home	Allied health and follow up services	6weeks/Baseline-1.5-3-6 months (20.4%)	QOL, MLHFQ, Anxiety, HADS. HF-related readmission rate, Cardiac-related emergency department visits.	QOL ↑. HF-related readmission rate, and Cardiac-related emergency department visits ↑ at 6 months. No significant differences were found in anxiety.
20. Boyde et al. (2018)/Australia	100/100	64.0±12.4/ 64.0±12.9	< 15: (14/18),16-25(35/32),26-35(26/33),≥36(25/17)	Class II (30/34); Class III (64.0/60.0);Class IV (5.0/5.0)	≤2:(38/28), 3-4:(38/47), ≥5:(24/25)	**Content:** self-care educational intervention (needs assessment, HF self-care knowledge and skills, manual, and DVD)<br>**No./length/frequency of session:** one 60~90-min session<br>**Format/delivery mode:** individual; face to face and DVD<br>**Setting:** hospital	Standard education	12-month/Baseline, 28 days, 3-12 months (14.5%)	All-cause hospital readmission. HF-related readmission ratemonth)	All-cause hospital readmission ↑ at 12 months. No significant differences were found in HF-related readmission rate

21.Atienza et al. (2004)/Spain	164/174	(median [IQR]): 69 (61–74) / 67 (58–74)	(median [IQR]): 36(30–53) / 40 (30–55)	Class I (11.0/10.0), Class II(39.0/40), Class III(40.0/40.0), Class IV(10.0/10.0)	DM(35/38), Hypertension(54/53),IHD(33/31), Valvular heart disease (24/29)	**Content:**Comprehensive discharge planning + outpatient HF management, including patient education (disease knowledge, symptom recognition, self-monitoring, medication adherence, self-adjustment of diuretics), family caregiver training, and 24-hour telephone consultation support.<br>**No./length/frequency of session:** Comprehensive discharge planning + outpatient HF management, including patient education (disease knowledge, symptom recognition, self-monitoring, medication adherence, self-adjustment of diuretics), family caregiver training, and 24-hour telephone consultation support.<br>**Format/delivery mode:** Nurse-led individualized education (supplemented with brochures); outpatient follow-up (cardiologist-led); telephone consultation.<br>**Setting:**Hospital (pre-discharge education), outpatient HF clinic (follow-up)	Usual care	509 days/ 12 month (Not explicitly reported)	Time to first event (readmission or death). All-cause readmission, HF-related readmission(6month), mortality, quality of life (MLHFQ), cost of care.	Time to first event (readmission or death). All-cause readmission, HF-related readmission, mortality, quality of life (MLHFQ), cost of care ↑.
22.Kotooka et al. (2018)/Japan	90/91	67.1±12.8/ 65.4±15.6	40.5±14.8/39.2±16.5	Class II (77.8/79.1), Class III(22.2/20.9)	History of IHD(31.1/29.7)	**Contents:** Home-based remote monitoring of physiological data (body weight, blood pressure, pulse); nurses monitored data daily via a central server and alerted physicians if thresholds were exceeded.<br>**No./length/frequency of session:** At least one measurement per day; nurse monitoring available 7 days/week, 9:00-17:00 daily.<br>**Format/delivery mode:** Electronic body-composition scale, blood pressure monitor, and wireless data-transmission device; patients performed self-measurements, and data were automatically uploaded to the central server.<br>**Setting:** home	Usual care	Until the end of follow-up (mean follow-up 15 months, range 0–31 months)./ Baseline to study end (mean 15 months). (17.8/14.3)	All-cause death or HF-related readmission rate, All-cause death, cardiovascular death, all-cause readmissions, cardiovascular readmissions, readmissions for worsening HF, changes in NT-proBNP level, changes in LVEF, MMSE score, GSES score, MLWHF score, PHQ-9 score.	No significant differences.

23.Cui et al. (2019)/China	48/48	55.1±13.4/ 56.6±12.8	43.5±3/ 42.1±2. 3	Class II (29.2/70. 8), Class III(25.0/ 75.0)	CHD(41.7/43.8) , Dllated cardiomyopath y(18.8/14.6), Valvular heart disease(12.5/14 .6), AF (18.8/14.6), Hypertension(27.1/31.3), DM(20.8/16.7)	**Content:** A structured education program covering etiology and risk factors of chronic heart failure, short- and long-term management goals; medication identification, side effects, and recognition of worsening symptoms; lifestyle modifications (diet, exercise), medication adherence and follow-up strategies; self-monitoring skills (blood pressure, pulse, body weight, fluid intake/output recording).<br>**No./length/frequency of session:** One 1-hour education session during hospitalization (after symptom stabilization); one 1-hour education session before discharge; follow-up every 8 weeks post-discharge in outpatient clinic, with monthly 15–30 minute phone or face-to-face consultations.<br>**Format/delivery mode:** Face-to-face teaching combined with printed materials and illustrations; family involvement encouraged; recording charts provided.<br>**Setting:** During hospitalization and after discharge (hospital, outpatient clinic, telephone follow-up).	Usual care	12 months / 12 months (0%)	HF-related readmission rate, medication adherence, dietary adjustment, social support, symptom control scores, daily weight measurement rate.	HF-related readmission rate, medication adherence, dietary adjustment, social support, symptom control scores, daily weight measurement rate↑ at 12-month. No significant differences were found in death rate and duration of readmission.
24.Mizukawa et al. (2019)/Japan A	18/19	69.4 ± 12.9/74.5 ± 12.1	(Mean) 42/42.1	NYHA III or IV(38.9/ 31.6)	Hypertension(50.0/63.2), DM (50.0/36.8)	**Content:**Self-management education (HF etiology, symptom management, diet/medication guidance)<br>**No./length/frequency of session:** Monthly 30-min face-to-face education sessions for the first 6 months, followed by monthly follow-up for the next 12 months<br>**Format/delivery mode:** Face-to-face education<br>**Setting:** hospital	Usual care	12 months/ 24 months (22.2%/16.1%)	MLWHFQ, Self-efficacy (CD-SES), self-care behavior (EHFScBS), HF-related readmission rate(24month), all-cause mortality, readmission + mortality)	No significant differences.
24.Mizukawa et al. (2019)/Japan B	20/19	70.5 ± 13.3/74.5 ± 12.1	(Mean) 42.2/42 .1	NYHA III or IV(55/3 1.6)	Hypertension(70.0/63.2), DM (45.0/36.8)	**Content:** Self-management education (including the etiology of heart failure, symptom management, dietary and medication guidance, etc.) + remote monitoring (daily body weight, blood pressure, and pulse measurement) + nurse-patient interactive communication (abnormal data feedback and nursing coordination).<br>**No./length/frequency of session:** One 30-minute face-to-face education session per month for the first 6 months, followed by monthly follow-up visits for	Usual care	12 months/ 24 months (25.0%/16.1%)	MLWHFQ, Self-efficacy (CD-SES), self-care behavior (EHFScBS), HF-related readmission rate(24month), all-cause mortality, readmission +	MLWHFQ and HF-related readmission rate↑. No significant differences were found in Self-efficacy (CD-SES), self-care behavior

						the subsequent 12 months; remote monitoring was implemented continuously for 12 months, with nurses reviewing the data daily and delivering telephone interventions as required.<br>**Format/delivery mode:** Face-to-face education + remote wireless data transmission + telephone communication.<br>**Setting:** Community-based home settings and hospital.			mortality)	(EHFScBS), and all-cause mortality, readmission + mortality).
25.Wonggom et al. (2020)/Australia	17/19	68.7±11.6/ 66.6±11.3	HFpEF (LVEF ≥50):(52.9/21.1), HFmEF(LVEF 40-49):(17.6/42.1),HFrEF(LVEF<40):(29.4/36.8)	Class I (52.9/47.4), Class II(47.1/36.8), Class III(0/15.8)	MI(35.3/42.1), PVD(11.8/5.3), Cerebrovascular(11.8/21.1), Chronic pulmonary disease(29.4/10.5), Peptic ulcer disease(23.5/5.3), DM (35.6/36.8), Renal disease (17.6/0)	**Content:** avatar educational application (HF self-care knowledge and skills, goal-setting, and action-planning) and usual care<br>**No./length/frequency of session:** not reported<br>**Format/delivery mode:** individual; APP<br>**Setting:** home	Usual care	3month/Baseline, 1 month, 3 months (2.8%)	Cardiac-related emergency department visits. HF-related readmission rate(3month)	No significant differences.
26.Negarandeh et al. (2019)/Iran	35/33	45-50:(42.9/42.4), 50-60:(22.9/30.3), 60-70:(34.3/27.3)	Not reported	Class II-III The specific values were not reported	Only reported as proportion with other diseases, without specifying types(77.1/84.8)	**Content:**Individualized education based on patient needs and HF self-care questionnaires, covering self-care behavior assessment, advice, education, follow-up, and re-evaluation; topics included symptom monitoring, diet, fluid management, exercise, etc.<br>**No./length/frequency of session:** Twice weekly (20 min/session) for the first month, then weekly (20 min/session) for the second month (adjustable based on patient needs).<br>**Format/delivery mode:** Telephone-based remote monitoring (tele-monitoring) with interactive education.<br>**Setting:** home	Usual care	2 months/1 month post-intervention (IG); final telephone follow-up (CG). (12.5/17.5)	HF self-care behavior (EHFScBS), HF-related readmission rate.	HF self-care behavior ↑. No significant differences were found in HF-related readmission rate.
27.Riegel et al. (2002)/USA	130/228	72.52±13.05/74.63±12	41.91±17.01/43.21±19.07	Class II (2.3/3.6) ,Class III (35.9/38.4), Class	COPD (24.6/42.0),CAD(55.4/64.2),CVA (10.8/9.3) DM (43.1/41.6), PVD	**Content:** Standardized telephone case management using decision-support software, focusing on HF readmission predictors (medication adherence, dietary management, symptom recognition, etc.) and integrating guideline-based best practices, patient education, data collection, and	Usual care	6 months/3 months and 6 months.<br>(Not reported)	HF-hospitalization rate(3month), All-cause hospitalization rate	HF-hospitalization rate ↑ at 3 months. HF-hospitalization rate, All-cause

				IV (61.7 / 58.0)	(19.2/15.5) Non-dialysis renal disease (32.3/25.7), Thyroid disease (12.3/17.3)	documentation.<br>**Number/Length/Frequency:** Average of 17 calls (median 14, range 11-22), total duration ~16 hours; first call within 5 days post-discharge, with subsequent frequency adjusted based on symptoms, knowledge, and needs (e.g., next-day follow-up for rapid weight gain, same-day follow-up for dyspnea).<br>**Format/Delivery Mode:** Nurse-led telephone follow-up guided by software algorithms and case-manager judgment.<br>**Setting:** Home environment post-discharge.			HF-related readmission rate, All-cause readmission rate, HF- hospital days, All-cause hospital days, Inpatient heart failure costs, Days to first readmissions, Multiple readmissions, Outpatient resource use (physician office visits, emergency department visits) , Patient satisfaction	hospitalization rate, HF-related readmission rate, All-cause readmission rate, HF-hospital days, All-cause hospital days, Inpatient heart failure costs, Days to first readmissions, Multiple readmissions, Outpatient resource use , and Patient satisfaction ↑ at 6 months. No significant differences were found in Outpatient resource use and Days to first readmissions.
28.Dizdarevic-Hudic et al. (2025)/Bosnia-Herzegovina [1]	32/32	most participants aged 56-65 years	HFrEF ≤40%	Not reported	Ischemic post infarction heart disease (53.13/46.88), VHD (21.88/28.13), Dilated Cardiomiopathy (15.63/18.75)	**Content:** Structured heart failure management education covering: disease knowledge (definition, etiology, symptoms); medication adherence (importance and side-effect management); lifestyle modification (sodium/fluid restriction, diet, exercise, smoking/alcohol cessation); self-monitoring (daily weight monitoring, symptom tracking); guidance on medical visits; psychosocial support; end-of-life planning.<br>**Number/Length/Frequency:** Not explicitly reported (only described as "structured education from trained nurses").<br>**Format/Delivery Mode:** Nurse-led structured education (mode not specified; presumably face-to-face).	Usual care	3 months/3 months(Not reported)	QoL (assessed via a questionnaire adapted from ESC, covering daily activity ability, depression, chest pain, etc.), HF-related readmission rate(3month).	QoL (assessed via a questionnaire adapted from ESC, covering daily activity ability, depression, chest pain, etc.), HF-related readmission rate ↑ at 3 months.

						**Setting:** Hospital-based (inpatient education during hospitalization for admitted patients; outpatient education during clinic visits for others).				
29.Yu et al. (2022)/China	118/118	69.1±7.7/70.7±8.3	43.0±14.2%/44.0±13.7%	Class II 81.4% Class III 18.6%/Class II 80.5% Class III 19.5%	CAD(50.0/45.8),Dilated cardiomyopathy (21.2/19.7), VHD (25.4/22.2), AF (40.7/38.5), Hypertension (53.4/58.5)	**Content:** empowerment-based self-care education program (HF manifestations and symptom monitoring, dietary and fluid modification, medication management, deteriorating symptom recognition and management, and advice on remaining physically active)<br>**No./length/frequency of session:** five 90-min weekly education sessions and three weekly and two bi-weekly telephone follow-ups<br>**Format/delivery mode:** group; face to face and telephone<br>**Setting:** hospital and home	Usual care	12 weeks/Baseline-3-6 months (16.1%)	QOL,MLHFQ, Cardiac-related emergency department visit, HF-related readmission rate(3month)	QOL ↑ at 3 months. Cardiac-related emergency room visit and HF-related readmission rate ↑.
30.Dekker et al. (2012)/USA	20/21	68.0±10.0/64.0±12.0	40.8±17/38.1±16.1	Class II (29/10), Class III (71/76), Class IV (0/14)	COPD (57/25) CAD (62/62), history of MI (38/43), AF(38/52), DM(43/48), renal insufficiency (43/33), stroke (19/19)	**Content:** brief cognitive therapy session (description of depression, connection between thoughts, feelings, and behaviors, description of a stressful event, thought-stopping, affirmations, and homework) and telephone booster session<br>**No./length/frequency of session:** one 30-min session and 5~10-min telephone booster session<br>**Format/delivery mode:** individual; face to face and telephone<br>**Setting:** hospital and home	Usual care	1 week/Baseline-1 week-3 months (17.1%)	QOL,MLHFQ, HF-related readmission rate Cardiac-related emergency department visits.	No significant differences.
31.Chew et al. (2021)/Singapore	72/72	58.4±14.0/62.8±10.5	33.5±13.1/33.9±12.0	Class I (43.1/47.2), Class II (55.6/45.8), Class III (1.4/ 7.0)	MI (58.3/65.3), Hypertension (58.3/72.2), DM (38.9/61.1), AF(23.6/41.7), AF(23.6/41.7), CKD(19.4/29.2)	**Content:** temporal self-regulation theory-based intervention (provision of HF self-care information, identification of motivation, goal-setting, action-planning, self-monitoring, self-regulation skills training, and coping planning, print booklet, and reinforcement telephone follow-up)<br>**No./length/frequency of session:** one 30-min face to face session and three 10-min reinforcement telephone follow-up sessions (week 3,6, and 9)<br>**Format/delivery mode:** individual; face to face and telephone<br>**Setting:** hospital and home	Usual care	3months/Baseline- 3-6 months (44.4%)	QOL ,MLHFQ, All-cause hospital readmission, HF-related readmission rate ,All-cause emergency department visits.	No significant differences.

32.Yanicelli et al. (2021)/Argentina	15/15	Not reported	35.93 ±12.13/ 29.8 ± 7.23	Class I (13.0/20.0),Class II (53.0/53.0),Class III (26.0/26.0),Class IV (6.0/0)	Hypertension (53.0/66.0), VHD(6.0/6.0), Dilated cardiomyopathy (40.0/46.0), Restrictive cardiomyopathy(6.0/6.0),DM(53.0/40.0)	**Content:** Daily collection of weight, blood pressure, heart rate, and symptoms (e.g., ankle swelling, dyspnea); provision of health education (FAQs, HF quiz game, daily health tips); alerts triggered by abnormal data. **Number/Length/Frequency:** Daily data collection and health education. **Format/Delivery Mode:** Mobile application (app) + web platform (clinician side); patient self-monitoring + clinician remote monitoring. **Setting:** Home	Usual care	3months/3months(33.3%/33.3%)	Self-care behavior (EHFScB), Treatment adherence (MMS) HF-related readmission rate	Self-care behavior (EHFScB) ↑ at 3 months. No significant differences were found in treatment adherence (MMS) And HF-related readmission rate.
33.Soran et al. (2008)/USA	160/155	76.9±7.1/76.0±6.8	24.3%±8.8/23.8%±8.7	Class II (57.5/59.3), Class III(42.5/40.7)	History of MI (74.7/82.4)	**Content:** Computer-assisted telephone-monitoring system (Alere DayLink HFMS), including daily weight monitoring, HF symptom assessment (e.g., nocturnal dyspnea, edema, fatigue), daily nurse data review, and alerts for abnormal findings. **Number/Length/Frequency:** Daily monitoring (weight + symptom questionnaire); nurse data review 7 days/week. **Format/Delivery Mode:** Home-based remote monitoring (electronic scale + telephone data transmission); nurse remote assessment + physician notification. **Setting:** Community-based primary care clinics.	patient education	6 months/6 months (2.5%)	Treatment failure (composite of cardiovascular death or HF-related readmission rate), hospital length of stay among patients readmitted for HF, All-cause hospitalization rate, HF-related readmission, emergency department visits, quality of life (SF-12, KCCQ).	No significant differences.
34. Dar et al. (2009)/UK	91 / 91	70±12.8 / 72 ±10.4	39% of all assessed patients had EF ≥40% (Preserved LV	Class II-IV at discharge (specific distribution not provided)	Previous myocardial infarction (44/53), Valve disease (5/5), Hypertension (60/63), Stroke (9/13), Diabetes (34/37),	**Content:** Daily home telemonitoring of weight, BP, heart rate, oxygen saturation, and 4 symptom questions. Data transmitted daily (Mon-Fri) and reviewed by HF nurse. Alerts triggered predefined responses (advice, medication change, contact GP, early review). **No./length/frequency of session:** Daily (weekday mornings). **Format/delivery mode:** Honeywell HomMed monitor connected to domestic phone line.	Usual care	6 months (4 withdrew post-consent; no loss to follow-up)	Days alive and out of hospital, number/duration of HF-related readmission rate(6 month), all-cause hospitalizations, clinic/ER visits, quality of life (MLwHF, EQ-5D),	Emergency HF hospitalizations and outpatient/ER visits ↓. No difference in days alive/out of hospital, all-cause hospitalization,

			systolic function: EG 39%, CG 40%)		COPD (9/9), Chronic renal failure (69/69)	**Setting:** home			costs	mortality, or quality of life.
35. Cleland et al. (2005)/United Kingdom, Germany, Netherlands A	168/85	67.0±13.0/ 68.0±10.0	25.0±8.0/24.0±8.0	Class I(5.0/ 6.0), Class II (16.0/13.0), Class III (18.0/30.0), Class IV (61.0/51.0)	CAD(61/68), Hypertension(44/40), Chronic AF(47/39),Chronic lung disease (24/29) DM(35/35), Stroke (9/8), CVD (38/36)	**Content:** Twice-daily self-monitoring of weight, blood pressure, heart rate and rhythm; data transmitted automatically via tele-device to a cardiac centre; plus nurse telephone support (identical to NTS group). **Number/Length/Frequency:** Twice daily (morning and evening). **Format/Delivery Mode:** Patient self-measurement (electronic scale, automatic BP monitor, single-lead ECG wrist-band electrode); automatic data transmission via telephone line to central server; nurse telephone support (monthly proactive calls + patient-initiated contact). **Setting:** Home.	Usual care	120 days, 240 days ,and 450 days/120 days, 240 days ,and 450days(1.8%/0%)	Death or days hospitalized , All-cause mortality, HF-related readmission rate, cardiovascular and non-cardiovascular hospital days, NYHA functional class, medication adherence.	Death or days hospitalized,NYHA functional class ↑ at 240 days. All-cause mortality ↑ at 12-month. No significant differences were found in HF-related readmission rate, cardiovascular and non-cardiovascular hospital days.
35.Cleland et al. (2005)/United Kingdom, Germany, Netherlands B	173/85	67.0±11.0/ 68.0±10.0	25.0±8.0/24.0±8.0	Class I(3.0/ 6.0), Class II (15.0/13.0), Class III (22.0/30.0), Class IV (61.0/51.0)	CAD(61/68), Hypertension(44/40), Chronic AF(47/39),Chronic lung disease (24/29) DM(35/35), Stroke (9/8), CVD (38/36)	**Content:** Specialist nurse telephone support (monthly proactive symptom and medication review, plus patient-initiated calls). **Number/Length/Frequency:** Monthly telephone follow-up; patients could call as needed. **Format/Delivery Mode:** Telephone communication. **Setting:** Home (via telephone).	Usual care	120 days, 240 days ,and 450 days/120 days, 240 days ,and 450 days(0.6%/0%)	Death or days hospitalized , All-cause mortality, HF-related readmission rate, cardiovascular and non-cardiovascular hospital days, NYHA functional class,	Death or days hospitalized, NYHA functional class ↑ at 240 days. All-cause mortality ↑ at 12-month.No significant differences were found in HF-related readmission rate, cardiovascular and non-cardiovascular

										hospital days.
36. Olivari et al. (2018)/Italy	229 / 110	79.6±6.8/ 80.9±7.3	39.1±12.9 / 39.1±14.6	Class II(48.0/48.2), Class III(47.2/46.4), Class IV(4.8/5.4)	Atrial fibrillation(34.9/48.2), Cerebrovascular diseases(9.2/6.4), COPD(19.7/19.1), Diabetes(38.9/26.4), Renal disease(29.7/31.8), Malignancy(9.6/12.7)	**Content:** Daily (5 days/week) self-measurement of heart rate, BP, 1-lead ECG, pulse oximetry, and weight using a Wrist-Clinic device and scale. Data transmitted to a regional eHealth center. Alerts reviewed by operators and clinicians for elective action.<br>**No./length/frequency of session:** Daily on weekdays.<br>**Format/delivery mode:** Wearable Wrist Clinic device + digital scale, telephone transmission.<br>**Setting:** home, linked to a regional monitoring centre	Usual care	12 months (39/229 (17%) in EG group did not receive the device post-randomization)	Primary: the combined occurrence of 12-month all-cause mortality or HF-related readmission rate(6month) Secondary: 12-month all cause mortality, number of hospitalisations for all causes and for heart failure, duration of hospitalisations, number of scheduled and urgent outpatient controls and health related quality of life (SF-36 v2)	Quality of life (SF-36 PCS and MCS) , trend for reduced primary endpoint and all-cause mortality ↑.No difference in primary endpoint or mortality.

37. Villani et al. (2014)/Italy	40 / 40	71±4 / 73±5	31±6 / 32±8	At enrolment: 3.08±0.57)/ 2.90±0.69. At 12 months: 2.08±0.38 / 2.40±0.45	Ischaemic heart disease(42.5/65), Diabetes(45/50), Hypercholesterolaemia(20/35)	**Content:** Daily use of a dedicated PDA for transmission of body weight, BP, HR; monthly psychological assessment (anxiety, depression, well-being); daily pill reminders; automatic questionnaires on compliance. Data reviewed daily by staff, therapy modified by cardiologist as needed.<br>**No./length/frequency of session:** Daily (vitals), monthly (psych assessment).<br>**Format/delivery mode:** Wireless PDA device with interactive software.<br>**Setting:** Patient's home, connected to monitoring centre.	Usual care	12 months (1 drop-out in EG group after a few days)	Mortality, hospital re-admissions for CHF(6month), NYHA class, plasma BNP, psychological status (STAI-6, PHQ-9, PGWBI), adherence (MMAS), drug titration, costs	HF-related readmission rate, composite endpoint of mortality+hospitalizations, NYHA class , anxiety/depression, perceived well-being and drug adherence ↑.
38. Antonicelli et al. (2008)/Italy	28 / 29	77±8/79±6	35±6/37±7	Class I(54/62), Class II(43/31), Class IV (4/7)	Not reported	**Content:** Weekly telephone contact by CHF team to collect symptoms, adherence, BP, HR, weight, urine output; weekly ECG transmission. Therapeutic regimen reassessed based on parameters.<br>**No./length/frequency of session:** At least once per week.<br>**Format/delivery mode:** Telephone (transtelephonic).<br>**Setting:** home.	Usual care	12 months (Attrition rate not explicitly stated; 1 lost in EG group before installation, implied low attrition).	Combined rate of mortality and hospitalization, mortality, hospitalization, quality of life (SF-36), therapy compliance, clinical variables, HF-related readmission rate (6month)	The composite endpoint of mortality and hospitalizations, hospital re-admission , therapy compliance and health perception score ↑.
39. Giordano et al. (2008)/Italy	230 / 230	58±10/56±10	28±7/26±8	Class II(54/65), Class III–IV(46/35)	Previous MI(53/51), Chronic atrial fibrillation(15/17), Chronic lung disease(28/26), Hypertension(19/23), Diabetes(29/27)	**Content:** 1) Scheduled telemonitoring: Weekly/fortnightly nurse-led standardized interview on clinical condition, diet, drugs, self-measured weight/BP; optional ECG transmission. 2) Teleassistance: Patient-initiated call for symptoms/doubts. Nurse could provide advice, adjust pre-planned therapy, or consult cardiologist/GP.<br>**No./length/frequency of session:** Scheduled: weekly/fortnightly; Unscheduled: as needed.<br>**Format/delivery mode:** Telephone with transtelephonic 1-lead ECG device (Card-Guard 2206).<br>**Setting:** Call Center supported by hospital nurses/cardiologists	Usual care	1 year (Median 296±91 days; EG: 4 lost, 3 discontinued; CG: 1 lost)	Unplanned hospital readmission for cardiovascular reasons, HF-related readmission rate (24 month), episodes of hemodynamic instability, cardiovascular mortality, costs	Cardiovascular readmission, HF-related readmission rate , episodes of hemodynamic instability, cardiovascular mortality, and mean cost for hospital readmission ↑.

Note: No., number; EG, experimental group; CG, control group; HF, heart failure; QOL, quality of life; ↑, significant improvement; ↓, significant deterioration; HADS, Hospital Anxiety and

Depression Scale; MLHFQ, Minnesota Living with Heart Failure Questionnaire; KCCQ, Kansas City Cardiomyopathy Questionnaire; MacNew, MacNew Heart Disease Health-related QOL Instrument; HFSD Scale, Heart Failure Symptom Distress Scale; HFSS, Heart Failure Symptom Survey; MSAS-HF, Memorial Symptom Assessment Scale–Heart Failure; PSQI, Pittsburgh Sleep Quality Index; SF-36, 36-Item Short-Form Health Survey; EQ-5D, EuroQol Five-Dimensional Questionnaire; DCM, dilated cardiomyopathy; RHD, rheumatic heart disease; ICM, ischemic cardiomyopathy; CPET, cardiopulmonary exercise test; 6MWT, 6-minute walk test; VO_2 peak, Peak oxygen consumption; AT, Anaerobic threshold; PVD, Peripheral vascular disease; CVA, cerebrovascular accident; COPD, chronic obstructive pulmonary disease; AMI, acute myocardial infarction; MI, Previous myocardial infarction; NYHA, New York Heart Association Classification; AF, atrial fibrillation; DM, diabetes mellitus; T2DM, Type 2 Diabetes Mellitus; HFNAQ, Heart Failure Needs Assessment Questionnaire; HFFSI, Heart Failure Functional Status Inventory; DFI, Dyspnea-Fatigue Index; PGRS, postglobal rating of symptoms; PGACQoL, Patient Global Assessment of Change in Quality of Life; CAD, coronary artery disease; IHD, ischemic heart disease; EDV, End-diastolic volume; ESV, End-systolic volume; EHFScBS, European Heart Failure Self-care Behavior Scale; SF-8, Short Form 8 Health Survey; PHQ-9, Patient Health Questionnaire-9; SPPB, Short Physical Performance Battery; ; SF-36 v2, 36-Item Short Form Health Survey, version 2.0; PDA, Personal Digital Assistant; BP, Blood Pressure; HR, Heart Rate; CHF, Chronic Heart Failure; BNP, B-type Natriuretic Peptide; STAI-6, the Spielberger's State-Trait Anxiety Inventory, 6-item version; PGWBI, the Perceived General Well-Being Index; MMAS, the Morisky Medication Adherence Scale; ECG, ElectroCardioGram; HIV, Human Immunodeficiency Virus; MMSE, Mini-Mental State Examination; GSES, General Self-Efficacy Scale; CD-SES, Chronic Disease Self-Efficacy Scale; MMS, Morisky Modified Scale.

(a)

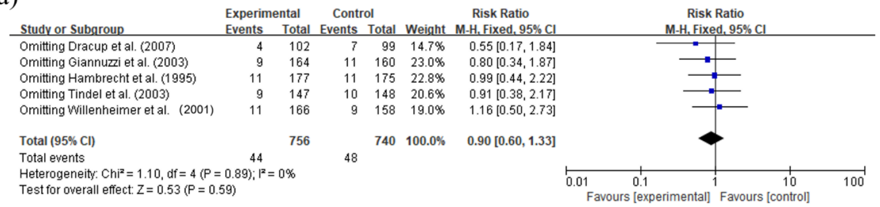

(b)

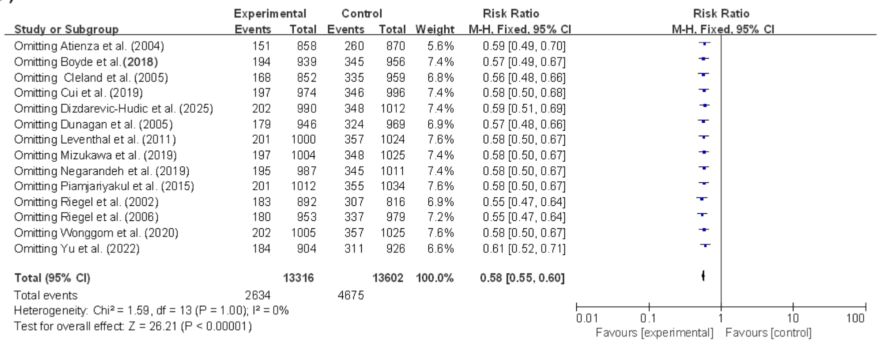

(c)

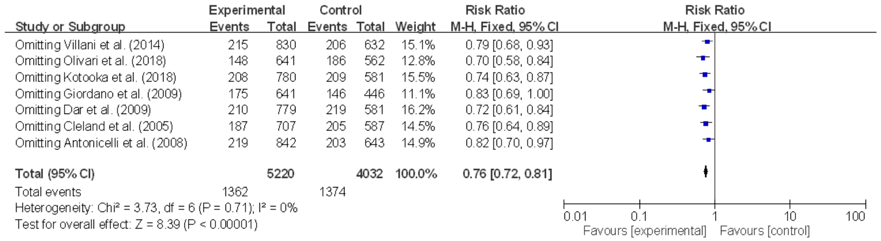

(d)

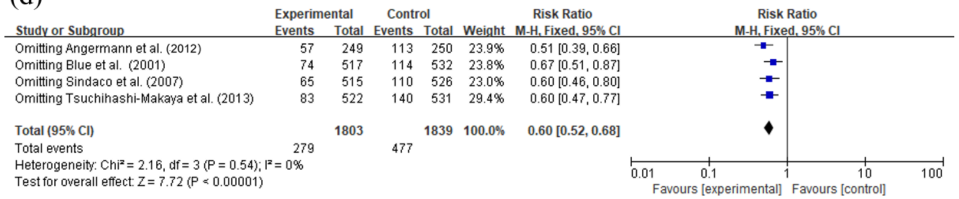

(e)

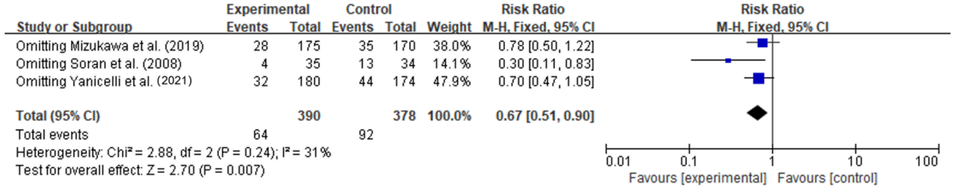

Figure S1. Sensitivity analysis of NPIs effects on HF-related readmission rates.

(a) Exercise therapy, (b) patient education, (c) health information tracking, (d) integrated care + patient education, and (e) health information tracking + patient education.

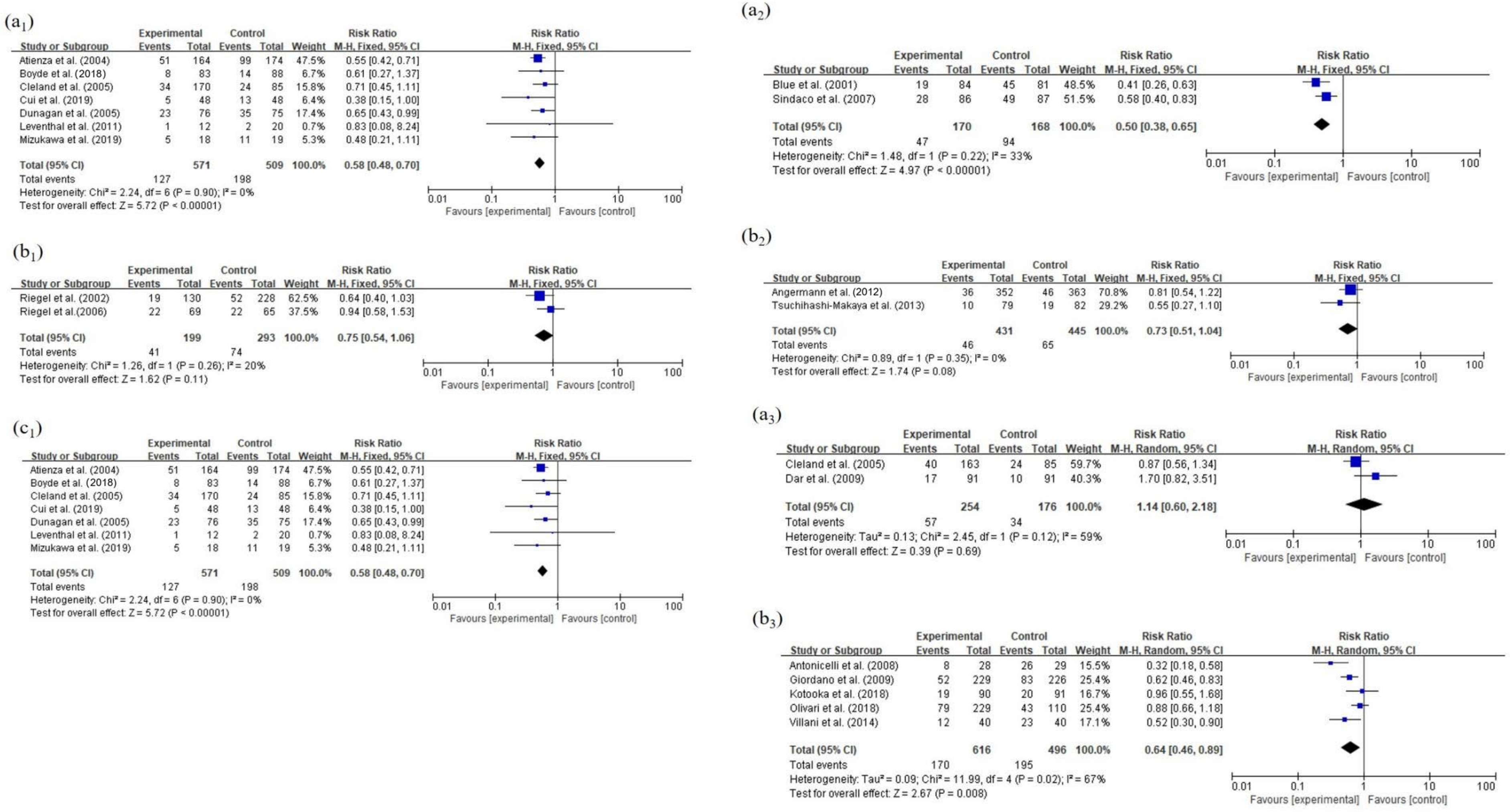

Figure S2. Subgroup analyses of the effects of NPIs on HF-related readmission rates.

Panels (a_1, b_1, c_1) show the effects of patient education stratified by intervention duration: (a_1) 12 months, (b_1) 6 months, and (c_1) 3 months.

Panels (a_2 and b_2) show integrated care + patient education stratified by the proportion of NYHA class I/II patients: (a_2) <40% and (b_2) >40%.

Panels (a_3 and b_3) show health information tracking stratified by intervention duration: (a_3) 6 months and (b_3) ≥12 months. Note: All comparisons are versus usual care.

Table S4. Sensitivity analysis of NPIs effects on HF-related readmission rates.

(a) Exercise therapy, (b) patient education, (c) health information tracking, (d) integrated care + patient education, and (e) health information tracking + patient education

(a)

Study	RR	95%CI	P-value	Chi^2	I^2(%)
Omitting Dracup et al. (2007)	0.61	[0.36; 1.84]	0.38	2.14	0
Omitting Giannuzzi et al. (2003)	0.82	[0.20; 1.86]	0.63	2.47	0
Omitting Hambrecht et al. (1995)	1.00	[0.45; 2.23]	1.00	2.14	0
Omitting Tindel et al. (2003)	0.91	[0.40; 2.11]	0.83	2.83	0
Omitting Willenheimer et al. (2001)	1.15	[0.50; 2.64]	0.73	1.19	0

(b)

Study	RR	95%CI	P-value	Chi^2	I^2(%)
Omitting Atienza et al. (2004)	0.57	[0.48; 0.68]	<0.001	15.73	17
Omitting Boyde et al. (2018)	0.56	[0.48; 0.65]	<0.001	15.96	19
Omitting Cleland et al. (2005)	0.55	[0.47; 0.64]	<0.001	15.20	14
Omitting Cui et al. (2019)	0.57	[0.49; 0.66]	<0.001	15.15	14
Omitting Dizdarevic-Hudic et al. (2025)	0.58	[0.50; 0.67]	<0.001	12.37	0
Omitting Dunagan et al. (2005)	0.55	[0.47; 0.65]	<0.001	15.68	17
Omitting Leventhal et al. (2011)	0.56	[0.49; 0.65]	<0.001	15.90	18
Omitting Mizukawa et al. (2019)	0.57	[0.49; 0.66]	<0.001	15.74	17
Omitting Negarandeh et al. (2019)	0.57	[0.49; 0.66]	<0.001	15.64	17
Omitting Piamjariyakul et al. (2015)	0.57	[0.49; 0.66]	<0.001	15.23	15
Omitting Riegel et al. (2002)	0.55	[0.47; 0.65]	<0.001	15.87	18
Omitting Riegel et al. (2006)	0.54	[0.46; 0.63]	<0.001	11.82	0
Omitting Wonggom et al. (2020)	0.56	[0.49; 0.65]	<0.001	15.51	16
Omitting Yu et al. (2022)	0.59	[0.51; 0.69]	<0.001	12.19	0

(c)

Study	RR	95%CI	P-value	Chi^2	I^2(%)
Omitting Antonicelli et al. (2008)	0.81	[0.63; 1.05]	0.11	10.41	52
Omitting Cleland et al. (2005)	0.71	[0.50; 1.01]	0.06	17.78	72
Omitting Dar et al. (2009)	0.68	[0.51; 0.89]	0.006	13.04	62
Omitting Giordano et al. (2009)	0.76	[0.53; 1.10]	0.15	16.61	70
Omitting Kotooka et al. (2018)	0.71	[0.51; 0.99]	0.04	17.48	71
Omitting Olivari et al. (2018)	0.71	[0.49; 1.02]	0.06	16.40	70
Omitting Villani et al. (2014)	0.52	[0.56; 1.08]	0.13	16.78	70

(d)

Study	RR	95%CI	P-value	Chi^2	I^2(%)
Omitting Angermann et al. (2012)	0.50	[0.39; 0.65]	<0.001	1.52	0
Omitting Blue et al. (2001)	0.66	[0.52; 0.86]	<0.001	1.75	0
Omitting Sindaco et al. (2007)	0.60	[0.45; 0.78]	<0.001	5.02	60
Omitting Tsuchihashi-Makaya et al. (2013)	0.60	[0.47; 0.75]	<0.001	4.99	60

(e)

Study	RR	95%CI	P-value	Chi^2	I^2(%)
Omitting Mizukawa et al. (2019)	0.78	[0.50; 1.22]	0.27	0.87	0
Omitting Soran et al. (2008)	0.32	[0.13; 0.80]	0.01	0.12	0
Omitting Yanicelli et al. (2021)	0.70	[0.47; 1.05]	0.09	2.58	61

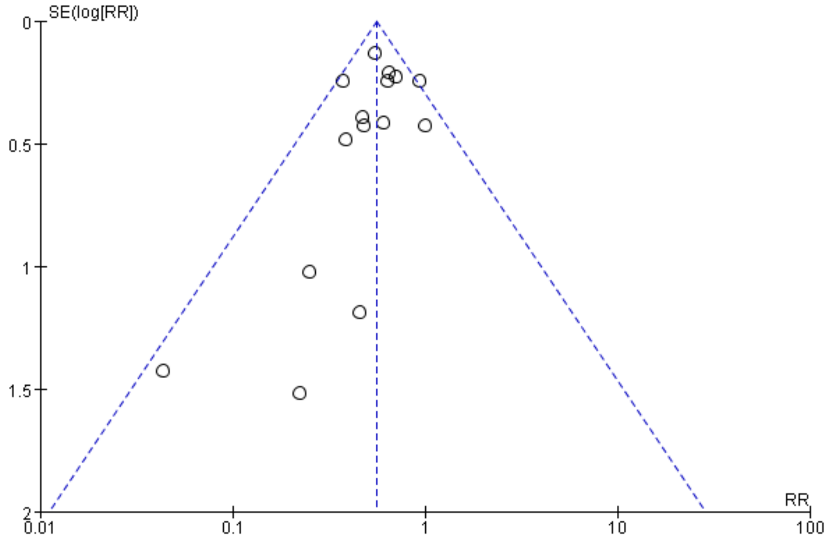

Figure S3. Funnel plots for the effects of patient education on HF-related readmission rates

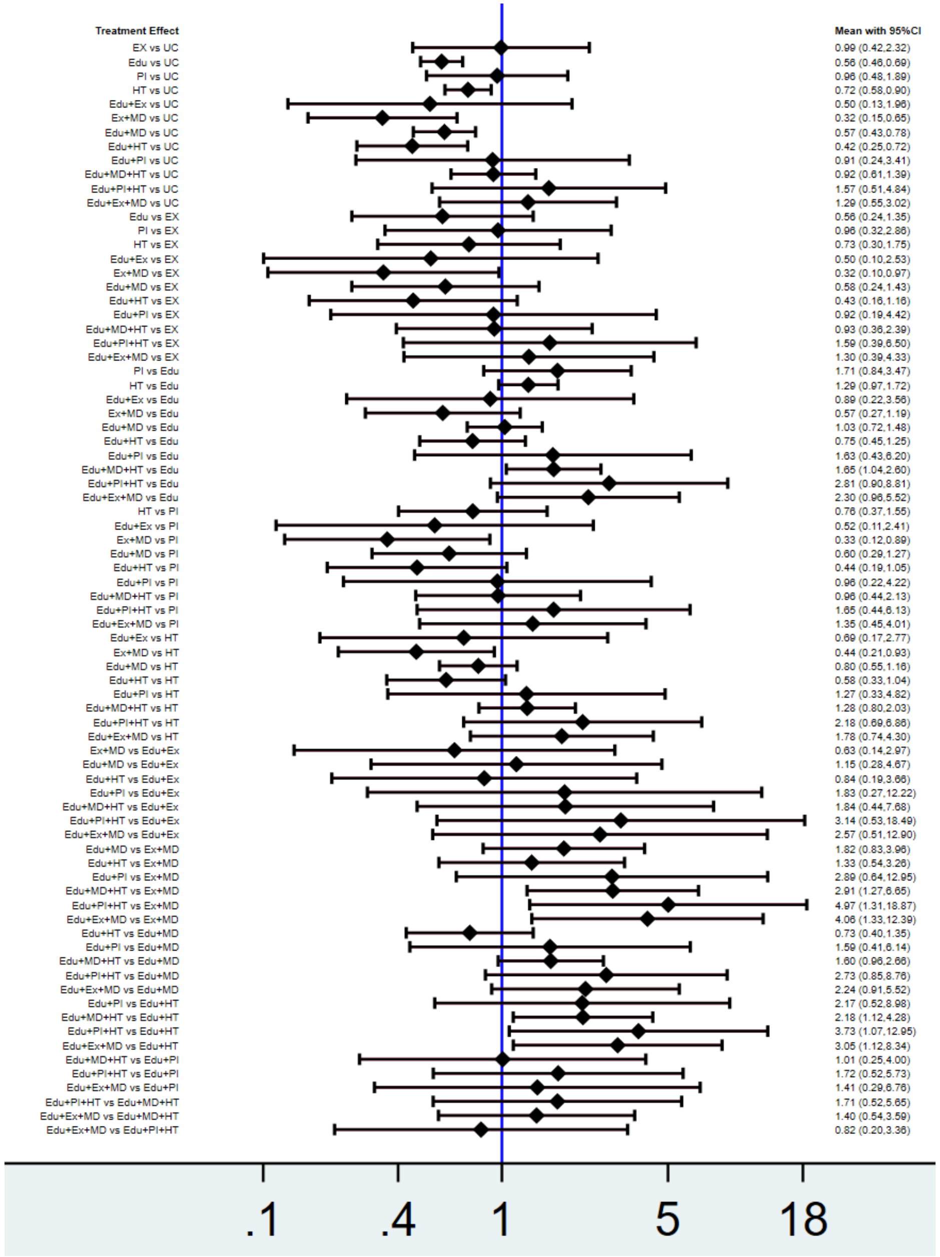

Figure S4. Forest plots for NPIs on overall HF-related readmission rates

UC	0.96 (0.48,1.89)	**0.72 (0.58,0.90)**	**0.32 (0.15,0.65)**	1.57 (0.51,4.84)	0.91 (0.24,3.41)	0.92 (0.61,1.39)	**0.57 (0.43,0.78)**	**0.42 (0.25,0.72)**	1.29 (0.55,3.02)	0.50 (0.13,1.96)	**0.56 (0.46,0.69)**	0.99 (0.42,2.32)
	PI	0.76 (0.37,1.55)	**0.33 (0.12,0.89)**	1.65 (0.44,6.13)	0.96 (0.22,4.22)	0.96 (0.44,2.13)	0.60 (0.29,1.27)	0.44 (0.19,1.05)	1.35 (0.45,4.01)	0.52 (0.11,2.41)	0.59 (0.29,1.19)	1.04 (0.35,3.09)
		HT	**0.44 (0.21,0.93)**	2.18 (0.69,6.86)	1.27 (0.33,4.82)	1.28 (0.80,2.03)	0.80 (0.55,1.16)	0.58 (0.33,1.04)	1.78 (0.74,4.30)	0.69 (0.17,2.77)	0.78 (0.58,1.03)	1.37 (0.57,3.31)
			Ex+MD	4.97 (1.31,18.87)	2.89 (0.64,12.95)	2.91 (1.27,6.65)	1.82 (0.83,3.96)	1.33 (0.54,3.26)	4.06 (1.33,12.39)	1.58 (0.34,7.41)	1.77 (0.84,3.73)	3.13 (1.03,9.53)
				Edu+PI+HT	0.58 (0.17,1.94)	0.59 (0.18,1.94)	0.37 (0.11,1.17)	**0.27 (0.08,0.93)**	0.82 (0.20,3.36)	0.32 (0.05,1.87)	0.36 (0.11,1.12)	0.63 (0.15,2.58)
					Edu+PI	1.01 (0.25,4.00)	0.63 (0.16,2.43)	0.46 (0.11,1.91)	1.41 (0.29,6.76)	0.55 (0.08,3.65)	0.61 (0.16,2.32)	1.08 (0.23,5.20)
						Edu+MD+HT	0.62 (0.38,1.04)	**0.46 (0.23,0.90)**	1.40 (0.54,3.59)	0.54 (0.13,2.26)	**0.61 (0.39,0.96)**	1.08 (0.42,2.77)
							Edu+MD	0.73 (0.40,1.35)	2.24 (0.91,5.52)	0.87 (0.21,3.53)	0.97 (0.68,1.40)	1.72 (0.70,4.25)
								Edu+HT	3.05 (1.12,8.34)	1.19 (0.27,5.15)	1.33 (0.80,2.21)	2.35 (0.86,6.41)
									Edu+Ex+MD	0.39 (0.08,1.95)	0.43 (0.18,1.04)	0.77 (0.23,2.57)
										Edu+Ex	1.12 (0.28,4.46)	1.98 (0.40,9.94)
											Edu	1.77 (0.74,4.25)
												EX

Table S5. Network Meta-Analysis of Effectiveness of NPIs (*RR* and *95%CI*)

(a)

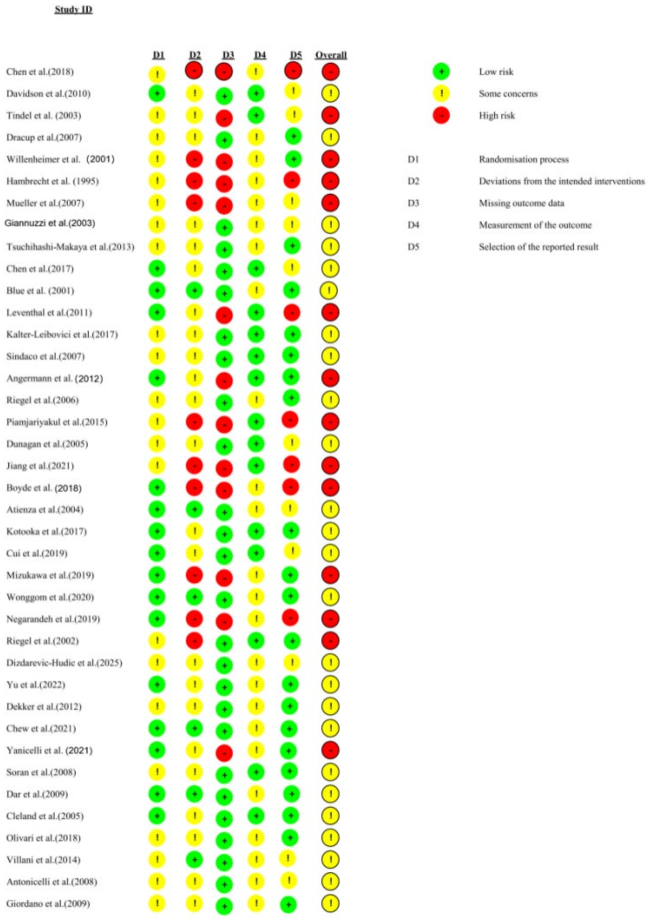

(b)

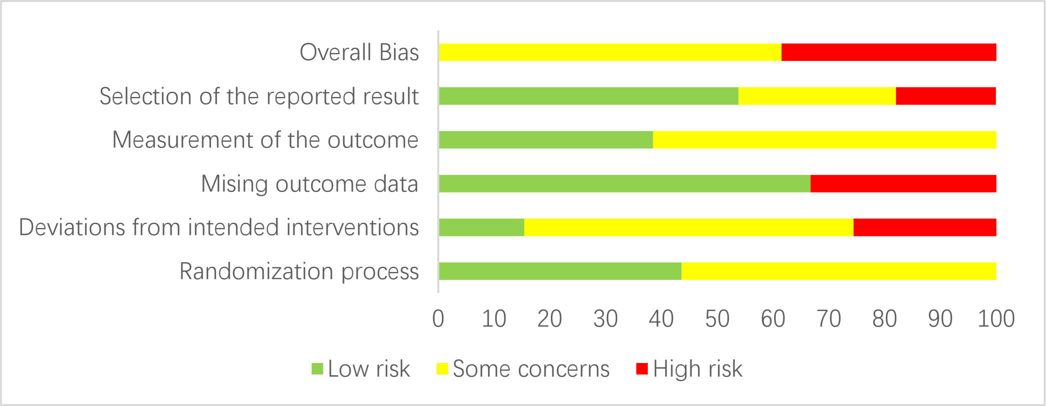

Figure S5. (a)Risk of bias summary
(b)Risk of bias graph.

Table S6. The evidence findings for all comparisons

Certainty assessment							№ of patients		Effect	Certainty
№ of studies	Study design	Risk of bias	Inconsistency	Indirectness	Imprecision	Publication bias	Intervention	Comparison	RR with 95% CI	
**Pairwise meta-analysis<br>Ex vs UC (overall)**										
5	RCT	Serious	Not serious	Not serious	Serious	Undetected	11/189	12/185	0.91 (0.42,1.96)	Low
**Pairwise meta-analysis<br>Edu vs UC (overall)**										
14	RCT	Not serious	Not serious	Not serious	Not serious	Undetected	194/992	351/1014	0.55 (0.48,0.64)	High
**Pairwise meta-analysis<br>HT vs UC (overall)**										
7	RCT	Not serious	Serious	Not serious	Not serious	Undetected	227/870	229/672	0.73 (0.54,0.99)	Moderate
**Pairwise meta-analysis<br>MD+Edu vs UC (overall)**										
4	RCT	Serious	Not serious	Not serious	Serious	Undetected	93/601	159/613	0.59 (0.47, 0.74)	Moderate
**Pairwise meta-analysis<br>HT+Edu vs UC (overall)**										
3	RCT	Serious	Not serious	Not serious	Serious	Undetected	32/195	46/189	0.68 (0.45,1.01)	Low
**NMA Ex vs UC**										
-	RCT	Serious	Not serious	Not serious	Serious	Undetected	-	-	0.99(0.42,2.32)	Low
**NMA Edu vs UC**										
-	RCT	Not serious	Not serious	Not serious	Not serious	Undetected	-	-	0.56 (0.46,0.69)	High
**NMA PI vs UC**										
-	RCT	Not serious	Not serious	Not serious	Serious	Undetected	-	-	0.96 (0.48,1.89)	Moderate
**NMA HT vs UC**										
-	RCT	Not serious	Serious	Not serious	Not serious	Undetected	-	-	0.72 (0.58,0.90)	Moderate
**NMA Edu+Ex vs UC**										

Certainty assessment							№ of patients		Effect	Certainty
№ of studies	Study design	Risk of bias	Inconsistency	Indirectness	Imprecision	Publication bias	Intervention	Comparison	RR with 95% CI	
-	RCT	Very serious	Not serious	Not serious	Serious	Undetected	-	-	0.50 (0.13,1.96)	Low
**NMA Ex+MD vs UC**										
-	RCT	Not serious	Not serious	Not serious	Not serious	Undetected	-	-	0.32 (0.15,0.65)	High
**NMA Edu+MD vs UC**										
-	RCT	Serious	Not serious	Not serious	Not serious	Undetected	-	-	0.57 (0.43,0.78)	Moderate
**NMA Edu+HT vs UC**										
-	RCT	Serious	Not serious	Not serious	Not serious	Undetected	-	-	0.42 (0.25,0.72)	Moderate
**NMA Edu+PI vs UC**										
-	RCT	Very serious	Not serious	Not serious	Very serious	Undetected	-	-	0.91 (0.24,3.41)	Low
**NMA Edu+MD+HT vs UC**										
-	RCT	Not serious	Not serious	Not serious	Serious	Undetected	-	-	0.92 (0.61,1.39)	Moderate
**NMA Edu+PI+HT vs UC**										
-	RCT	Very serious	Not serious	Not serious	Very serious	Undetected	-	-	1.57 (0.51,4.84)	Very Low
**NMA Edu+EX+MD vs UC**										
-	RCT	Not serious	Not serious	Not serious	Very serious	Undetected	-	-	1.29 (0.55,3.02)	Low
**NMA Edu vs Ex**										
-	RCT	Not serious	Not serious	Not serious	Serious	Undetected	-	-	0.56 (0.24,1.35)	Moderate
**NMA PI vs Ex**										
-	RCT	Not serious	Not serious	Not serious	Very serious	Undetected	-	-	0.96 (0.32,2.86)	Low
**NMA HT vs Ex**										
-	RCT	Not serious	Not serious	Not serious	Serious	Undetected	-	-	0.73 (0.30,1.75)	Moderate
**NMA Edu+Ex vs Ex**										
-	RCT	Not serious	Not serious	Not serious	Serious	Undetected			0.50 (0.10,2.53)	Moderate
**NMA Ex+MD vs Ex**										
-	RCT	Not serious	Not serious	Not serious	Not serious	Undetected	-	-	0.32 (0.10,0.97)	High

Certainty assessment							№ of patients		Effect	Certainty
№ of studies	Study design	Risk of bias	Inconsistency	Indirectness	Imprecision	Publication bias	Intervention	Comparison	RR with 95% CI	
**NMA Edu+MD vs Ex**										
-	RCT	Not serious	Not serious	Not serious	Serious	Undetected	-	-	0.58 (0.24,1.43)	Moderate
**NMA Edu+HT vs Ex**										
-	RCT	Not serious	Not serious	Not serious	Serious	Undetected	-	-	0.43 (0.16,1.16)	Moderate
**NMA Edu+PI vs Ex**										
-	RCT	Not serious	Not serious	Not serious	Very serious	Undetected	-	-	0.92 (0.19,4.42)	Low
**NMA Edu+MD+HT vs Ex**										
-	RCT	Not serious	Not serious	Not serious	Very serious	Undetected	-	-	0.93 (0.36,2.39)	Low
**NMA Edu+PI+HT vs Ex**										
-	RCT	Not serious	Not serious	Not serious	Very serious	Undetected	-	-	1.59 (1.39,6.50)	Low
**NMA Edu+Ex+MD vs Ex**										
-	RCT	Not serious	Not serious	Not serious	Very serious	Undetected	-	-	1.30 (0.39,4.33)	Low
**NMA PI vs Edu**										
-	RCT	Not serious	Not serious	Not serious	Very serious	Undetected	-	-	1.71 (0.84,3.47)	Low
**NMA HT vs Edu**										
-	RCT	Not serious	Not serious	Not serious	Serious	Undetected	-	-	1.29 (0.97,1.72)	Moderate
**NMA Edu+Ex vs Edu**										
-	RCT	Not serious	Not serious	Not serious	Very serious	Undetected	-	-	0.89 (0.22,3.56)	Low
**NMA Ex+MD vs Edu**										
-	RCT	Not serious	Not serious	Not serious	Serious	Undetected	-	-	0.57 (0.27,1.19)	Moderate
**NMA Edu+MD vs Edu**										
-	RCT	Not serious	Not serious	Not serious	Serious	Undetected	-	-	1.03 (0.72,1.48)	Moderate
**NMA Edu+HT vs Edu**										
-	RCT	Not serious	Not serious	Not serious	Serious	Undetected	-	-	0.75 (0.45,1.25)	Moderate
**NMA Edu+PI vs Edu**										

Certainty assessment							№ of patients		Effect	Certainty
№ of studies	Study design	Risk of bias	Inconsistency	Indirectness	Imprecision	Publication bias	Intervention	Comparison	RR with 95% CI	
-	RCT	Not serious	Not serious	Not serious	Very serious	Undetected	-	-	1.63 (0.43,6.20)	Low
**NMA Edu+MD+HT vs Edu**										
-	RCT	Not serious	Not serious	Not serious	Very serious	Undetected	-	-	1.65 (1.04,2.60)	Low
**NMA Edu+PI+HT vs Edu**										
-	RCT	Not serious	Not serious	Not serious	Very serious	Undetected	-	-	2.81 (0.90,8.81)	Low
**NMA Edu+Ex+MD vs Edu**										
-	RCT	Not serious	Not serious	Not serious	Very serious	Undetected	-	-	2.30 (0.96,5.52)	Low
**NMA HT vs PI**										
-	RCT	Not serious	Not serious	Not serious	Serious	Undetected	-	-	0.76 (0.37,1.55)	Moderate
**NMA Edu+Ex vs PI**										
-	RCT	Not serious	Not serious	Not serious	Very serious	Undetected	-	-	0.52 (0.11,2.41)	Low
**NMA Ex+MD vs PI**										
-	RCT	Not serious	Not serious	Not serious	Not serious	Undetected	-	-	0.33 (0.12,0.89)	High
**NMA Edu+MD vs PI**										
-	RCT	Not serious	Not serious	Not serious	Serious	Undetected	-	-	0.60 (0.29,1.27)	Moderate
**NMA Edu+HT vs PI**										
-	RCT	Not serious	Not serious	Not serious	Serious	Undetected	-	-	0.44 (0.19,1.05)	Moderate
**NMA Edu+PI vs PI**										
-	RCT	Not serious	Not serious	Very serious	Very serious	Undetected	-	-	0.96 (0.22,4.22)	Very Low
**NMA Edu+MD+HT vs PI**										
-	RCT	Not serious	Not serious	Not serious	Very serious	Undetected	-	-	0.96 (0.44,2.13)	Low
**NMA Edu+PI+HT vs PI**										
-	RCT	Not serious	Not serious	Not serious	Very serious	Undetected	-	-	1.65 (0.44,6.13)	Low
**NMA Edu+Ex+MD vs PI**										
-	RCT	Not serious	Not serious	Not serious	Very serious	Undetected	-	-	1.35 (0.45,4.01)	Low

Certainty assessment							№ of patients		Effect	Certainty
№ of studies	Study design	Risk of bias	Inconsistency	Indirectness	Imprecision	Publication bias	Intervention	Comparison	RR with 95% CI	
**NMA Edu+Ex vs HT**										
-	RCT	Not serious	Not serious	Not serious	Very serious	Undetected	-	-	0.69 (0.17,2.77)	Low
**NMA Ex+MD vs HT**										
-	RCT	Not serious	Not serious	Not serious	Not serious	Undetected	-	-	0.44 (0.21,0.93)	High
**NMA Edu+MD vs HT**										
-	RCT	Not serious	Not serious	Not serious	Serious	Undetected	-	-	0.80(0.55,1.16)	Moderate
**NMA Edu+HT vs HT**										
-	RCT	Not serious	Not serious	Not serious	Serious	Undetected	-	-	0.58 (0.33,1.04)	Moderate
**NMA Edu+PI vs HT**										
-	RCT	Not serious	Not serious	Not serious	Very serious	Undetected	-	-	1.27 (0.33,4.82)	Low
**NMA Edu+MD+HT vs HT**										
-	RCT	Serious	Not serious	Not serious	Very serious	Undetected	-	-	1.28 (0.80,2.03)	Low
**NMA Edu+PI+HT vs HT**										
-	RCT	Not serious	Not serious	Not serious	Very serious	Undetected	-	-	2.18 (0.69,6.86)	Low
**NMA Edu+Ex+MD vs HT**										
-	RCT	Not serious	Not serious	Not serious	Very serious	Undetected	-	-	1.78 (0.74,4.30)	Low
**NMA Ex+MD vs Edu+Ex**										
-	RCT	Not serious	Not serious	Not serious	Very serious	Undetected	-	-	0.63 (0.14,2.97)	Low
**NMA Edu+MD vs Edu+Ex**										
-	RCT	Not serious	Not serious	Not serious	Very serious	Undetected	-	-	1.15 (0.28,4.67)	Low
**NMA Edu+HT vs Edu+Ex**										
-	RCT	Not serious	Not serious	Not serious	Very serious	Undetected	-	-	0.84 (0.19,3.66)	Low
**NMA Edu+PI vs Edu+Ex**										
-	RCT	Not serious	Not serious	Not serious	Very serious	Undetected	-	-	1.83 (0.27,12.22)	Low
**NMA Edu+MD+HT vs Edu+Ex**										

Certainty assessment							№ of patients		Effect	Certainty
№ of studies	Study design	Risk of bias	Inconsistency	Indirectness	Imprecision	Publication bias	Intervention	Comparison	RR with 95% CI	
-	RCT	Not serious	Not serious	Not serious	Very serious	Undetected	-	-	1.84 (0.44,7.68)	Low
**NMA Edu+PI+HT vs Edu+Ex**										
-	RCT	Not serious	Not serious	Not serious	Very serious	Undetected	-	-	3.14 (0.53,18.49)	Low
**NMA Edu+Ex+MD vs Edu+Ex**										
-	RCT	Not serious	Not serious	Not serious	Very serious	Undetected	-	-	2.57 (0.51,12.90)	Low
**NMA Edu+MD vs Ex+MD**										
-	RCT	Not serious	Not serious	Not serious	Very serious	Undetected	-	-	1.82 (0.83,3.96)	Low
**NMA Edu+HT vs Ex+MD**										
-	RCT	Not serious	Not serious	Not serious	Very serious	Undetected	-	-	1.33 (0.54,3.26)	Low
**NMA Edu+PI vs Ex+MD**										
-	RCT	Not serious	Not serious	Not serious	Very serious	Undetected	-	-	2.89 (0.64,12.95)	Low
**NMA Edu+MD+HT vs Ex+MD**										
-	RCT	Not serious	Not serious	Not serious	Very serious	Undetected	-	-	2.91 (1.27,6.65)	Low
**NMA Edu+PI+HT vs Ex+MD**										
-	RCT	Not serious	Not serious	Not serious	Very serious	Undetected	-	-	4.97 (1.31,18.87)	Low
**NMA Edu+Ex+MD vs Ex+MD**										
-	RCT	Not serious	Not serious	Not serious	Very serious	Undetected	-	-	4.06 (1.33,12.39)	Low
**NMA Edu+HT vs Edu+MD**										
-	RCT	Not serious	Not serious	Not serious	Serious	Undetected	-	-	0.73 (0.40,1.35)	Moderate
**NMA Edu+PI vs Edu+MD**										
-	RCT	Not serious	Not serious	Not serious	Very serious	Undetected	-	-	1.59 (0.41,6.14)	Low
**NMA Edu+MD+HT vs Edu+MD**										
-	RCT	Not serious	Not serious	Not serious	Very serious	Undetected	-	-	1.60 (0.96,2.66)	Low
**NMA Edu+PI+HT vs Edu+MD**										
-	RCT	Not serious	Not serious	Not serious	Very serious	Undetected	-	-	2.73 (0.85,8.76)	Low

Certainty assessment							№ of patients		Effect	Certainty
№ of studies	Study design	Risk of bias	Inconsistency	Indirectness	Imprecision	Publication bias	Intervention	Comparison	RR with 95% CI	
**NMA Edu+Ex+MD vs Edu+MD**										
-	RCT	Not serious	Not serious	Not serious	Very serious	Undetected	-	-	2.24 (0.91,5.52)	Low
**NMA Edu+PI vs Edu+HT**										
-	RCT	Not serious	Not serious	Not serious	Very serious	Undetected	-	-	2.17 (0.52,8.98)	Low
**NMA Edu+MD+HT vs Edu+HT**										
-	RCT	Not serious	Not serious	Not serious	Very serious	Undetected	-	-	2.18 (1.12,4.28)	Low
**NMA Edu+PI+HT vs Edu+HT**										
-	RCT	Not serious	Not serious	Not serious	Very serious	Undetected	-	-	3.73 (1.07,12.95)	Low
**NMA Edu+Ex+MD vs Edu+HT**										
-	RCT	Not serious	Not serious	Not serious	Very serious	Undetected	-	-	3.05 (1.12,8.34)	Low
**NMA Edu+MD+HT vs Edu+PI**										
-	RCT	Not serious	Not serious	Not serious	Very serious	Undetected	-	-	1.01 (0.25,4.00)	Low
**NMA Edu+PI+HT vs Edu+PI**										
-	RCT	Serious	Not serious	Not serious	Very serious	Undetected	-	-	1.72 (0.52,5.73)	Very Low
**NMA Edu+Ex+MD vs Edu+PI**										
-	RCT	Not serious	Not serious	Not serious	Very serious	Undetected	-	-	1.41 (0.29,6.76)	Low
**NMA Edu+PI+HT vs Edu+MD+HT**										
-	RCT	Not serious	Not serious	Not serious	Very serious	Undetected	-	-	1.71 (0.52,5.65)	Low
**NMA Edu+Ex+MD vs Edu+MD+HT**										
-	RCT	Not serious	Not serious	Not serious	Very serious	Undetected	-	-	1.40 (0.54,3.59)	Low
**NMA Edu+Ex+MD vs Edu+PI+HT**										
-	RCT	Not serious	Not serious	Not serious	Very serious	Undetected	-	-	0.82 (0.20,3.36)	Low

Table S7. Transitivity assessment of potential effect modifiers across intervention nodes

Intervention node	No. of studies	Included studies	Participants (EG/CG)					Intervention	Duration/Follow-up
			Sample size	Mean age	LVEF (%)	NYHA (%)	Comorbidities (%)	the specific content of the intervention	
Exercise therapy + Multidisciplinary team management (Ex+MD)	(n=1)	Davidson et al. (2010)	53/52	71.6/73.9	Not reported	Class I(3.8/0),Class II(37.7/32.7),Class III(60.4/67.3)	COPD(11.0/13.0), DM(26.4/26.9), PVD(28.3/32.7), AF(47.1/46.1), Previous AMI(62.3/55.8), OSA(11.3/7.7), Stroke(5.7/11.5), Arthritis(60.4/53.9), Hypertension(79.3/69.2),	**No./length/frequency of session:** 12-week program; weekly 30-min hospital-based exercise sessions; monthly telephone follow-up. **Format/delivery mode:** Group education and individualized exercise guidance with a home-based exercise plan; intermittent monitoring of heart rate, blood pressure, and oxygen saturation. **Setting:** Hospital outpatient clinic	3 months/ 3 months, 12 months
Patient education + Health information tracking (Edu+HT)	(n=3)	Soran et al. (2008)	160/155	76.9±7.1/76.0±6.8	24.3%±8.8/23.8%±8.7	Class II (57.5/59.3), Class III(42.5/40.7)	History of MI (74.7/82.4)	**No./Length/Frequency:** Daily monitoring (weight + symptom questionnaire); nurse data review 7 days/week. **Format/Delivery Mode:** Home-based remote monitoring (electronic scale + telephone data transmission); nurse remote assessment + physician notification. **Setting:** Community-based primary care clinics.	6 months/6 months
		Mizukawa et al. (2019)	20/19	70.5 ± 13.3/74.5 ± 12.1	(Mean)42.2/42.1	Class III or IV (55/31.6)	Hypertension(70.0/63.2), DM (45.0/36.8)	**No./length/frequency of session:** One 30-minute face-to-face education session per month for the first 6 months, followed by monthly follow-up visits for the subsequent 12 months; remote monitoring was implemented continuously for 12 months, with nurses reviewing the data daily and delivering telephone interventions as required. **Format/delivery mode:** Face-to-face education + remote wireless data transmission + telephone communication. **Setting:** Community-based home settings and hospital.	12 months/ 24 months
		Yanicelli et al. (2020)	15/15	Not reported	35.93 ±12.13 /29.8 ±	Class I (13.0/20.0) ,Class II	Hypertension (53.0/66.0), VHD(6.0/6.0),	**Number/Length/Frequency:** Daily data collection and health education. **Format/Delivery Mode:** Mobile application (app) + web	3months/3months

					7.23	(53.0/53.0),Class III (26.0/26.0),Class IV (6.0/0)	Dilated cardiomyopathy (40.0/46.0), Restrictive cardiomyopathy (6.0/6.0),DM(53.0/40.0)	platform (clinician side); patient self-monitoring + clinician remote monitoring.<br>**Setting:** Home	
Patient education (Edu)	(n=14)	Mizukawa et al. (2019)	20/19	70.5 ± 13.3/74.5 ± 12.1	(Mean)42.2/42.1	Class III or IV (55/31.6)	Hypertension(70.0/63.2), DM (45.0/36.8)	**No./length/frequency of session:** One 30-minute face-to-face education session per month for the first 6 months, followed by monthly follow-up visits for the subsequent 12 months; remote monitoring was implemented continuously for 12 months, with nurses reviewing the data daily and delivering telephone interventions as required.<br>**Format/delivery mode:** Face-to-face education + remote wireless data transmission + telephone communication.<br>**Setting:** Community-based home settings and hospital.	12 months/ 24 months
		Dunagan et al. (2005)	76/75	70.5±12.7/69.4±13.9	<25:(38/48); 25-40% (36/31);41-50:(8/7); >50 (18/15)	Class II (22.0/17.0);Class III (71.0/72.0);Class IV (7.0/11.0)	There was no significant difference in baseline comorbidities between the intervention and control groups	**No./length/frequency of session:** Initial call within 3 days post-discharge, followed by at least one call per week for the next 2 weeks. Subsequent call frequency was adjusted based on the patient's clinical status and self-management capability (no fixed session duration recorded).<br>**Format/delivery mode:** Telephone follow-up; supplemented with home visits for a subset of patients (n=20), and provision of weight scales to 18 patients.<br>**Setting:** Hospital-led telephone follow-up, combined with home visits for selected patients.	1 year/ 6 months, 12 months
		Piamjariyakul et al. (2015)	10/10	65.1±8.0/57.3±10.9	Not reported	Not reported	The overall distribution of IG and EG(55), MI or cardiovascular disease (20), DM (20), osteoarthritis/pain (20), depression(5); thyroid problems, asthma(5), and HIV(5).There was no statistically	**No./length/frequency of session:** 4-week telephone coaching, 1 session per week, 60-90 minutes per session.<br>**Format/delivery mode:** Telephone coaching combined with educational materials (AHA guidelines, low-sodium diet handbook, pill organizers, etc.).<br>**Setting:** home	4 weeks/6 months

					significant difference in the baseline between the two groups		
Riegel et al. (2002)	130/228	72.52±13.05/74.63±12	41.91±17.01/43.21±19.07	Class II (2.3/3.6), Class III (35.9/38.4), Class IV (61.7 / 58.0)	COPD (24.6/42.0),CAD(55.4/64.2),CVA (10.8/9.3) DM (43.1/41.6), PVD (19.2/15.5) Non-dialysis renal disease (32.3/25.7), Thyroid disease (12.3/17.3)	**Number/Length/Frequency:** Average of 17 calls (median 14, range 11-22), total duration ~16 hours; first call within 5 days post-discharge, with subsequent frequency adjusted based on symptoms, knowledge, and needs (e.g., next-day follow-up for rapid weight gain, same-day follow-up for dyspnea).<br>**Format/Delivery Mode:** Nurse-led telephone follow-up guided by software algorithms and case-manager judgment.<br>**Setting:** Home environment post-discharge.	6 months/3 months and 6 months
Riegel et al. (2006)	69/65	71.6±10.8/72.7±11.2	42.3±18.3/44.1±18.1	Class II (17.4/20.0),Class III (44.9/47.7), Class IV(37.7 / 32.3)	Hypertension (84.1/73.8); COPD(21.7/33.8); History of MI(26.1/29.2); Diabetes(55.1/63.1)%;Diabetes with end-organ damage (17.4/18.5);Renal disease with creatinine >3 mg(8.7/4.6)	**No./length/frequency of session:** Average 13.5 patient contacts (SD 5.9) + 8.4 family contacts (SD 6.3) over 6 months; most calls in the first month.<br>**Format/delivery mode:** Telephone calls by bilingual/bicultural Mexican-American registered nurses; educational materials in Spanish/English.<br>**Setting:** Community-based (post-hospital discharge).	6 months /Baseline, 1 month, 3 months, 6 months
Cui et al. (2019)	48/48	55.1±13.4/56.6±12.8	43.5±3/42.1±2.3	Class II (29.2/70.8), Class III(25.0/75.0)	CHD(41.7/43.8), Dllated cardiomyopathy (18.8/14.6), Valvular heart disease(12.5/14.6), AF (18.8/14.6), Hypertension(27.1/31.3), DM(20.8/16.7)	**No./length/frequency of session:** One 1-hour education session during hospitalization (after symptom stabilization); one 1-hour education session before discharge; follow-up every 8 weeks post-discharge in outpatient clinic, with monthly 15–30 minute phone or face-to-face consultations.<br>**Format/delivery mode:** Face-to-face teaching combined with printed materials and illustrations; family involvement encouraged; recording charts provided.<br>**Setting:** During hospitalization and after discharge (hospital, outpatient clinic, telephone follow-up).	12months/12months
Yu et al. (2022)	118/118	69.1±7.7/70.7±8.3	43.0±14.2%/44.0±1	Class II 81.4% Class III	CAD(50.0/45.8), Dilated cardiomyopathy	**No./length/frequency of session:** five 90-min weekly education sessions and three weekly and two bi-weekly telephone follow-ups	

			3.7%	18.6%/Class II 80.5% Class III 19.5%	(21.2/19.7), VHD (25.4/22.2), AF (40.7/38.5), Hypertension (53.4/58.5)	**Format/delivery mode:** group; face to face and telephone<br>**Setting:** hospital and home	
Boyde et al. (2018)	100/100	64.0±12.4/64.0±12.9	＜15: (14/18),16-25(35/32),26-35(26/33),≥36(25/17)	Class II (30/34);Class III (64.0/60.0);Class IV (5.0/5.0)	≤2:(38/28), 3-4:(38/47), ≥5:(24/25)	**No./length/frequency of session:** one 60~90-min session<br>**Format/delivery mode:** individual; face to face and DVD<br>**Setting:** hospital	12-month/Baseline, 28 days, 3-12 months
Wonggom et al. (2020)	17/19	68.7±11.6/66.6±11.3	HFpEF(LVEF≥50):(52.9/21.1), HFmEF(LVEF 40-49):(17.6/42.1),HFrEF(LVEF<40):(29.4/36.8)	Class I (52.9/47.4), Class II(47.1/36.8), Class III(0/15.8)	MI(35.3/42.1), PVD(11.8/5.3),Cerebrovascular(11.8/21.1), Chronic pulmonary disease(29.4/10.5), Peptic ulcer disease(23.5/5.3), DM (35.6/36.8), Renal disease (17.6/0)	**No./length/frequency of session:** not reported<br>**Format/delivery mode:** individual; APP<br>**Setting:** home	3month/Baseline, 1 month, 3 months
Leventhal et al. (2011)	22/20	76.7±7.1/77.6±6.0	45.0 (30.0~60.0)/42.0(28.0~57.5)	Class I (16.7/15.0), Class II (35.0/56.3), Class III(50.0/25.0)	DM(31.8/20.0)	**No./length/frequency of session:**1 home visit (approximately 1 week post-intervention) + 17 telephone follow-ups (decreasing frequency: once weekly for the first 4 weeks, once every 2 weeks for the next 4 weeks, and once monthly for the subsequent 6 months)<br>**Format/delivery mode:** Home visits (face-to-face) + telephone follow-up; individualized care plan<br>**Setting:** Outpatient clinic/home	12 months/12months
Dizdarevic-Hudic et al.	32/32	most participants	HFrEF ≤40%	Not reported	Ischemic post infarction heart disease	**Number/Length/Frequency:** Not explicitly reported (only described as "structured education from trained nurses").	3 months/3 months

	(2025)		aged 56-65 years			(53.13/46.88), VHD (21.88/28.13), Dilated Cardiomiopathy (15.63/18.75)	**Format/Delivery Mode:** Nurse-led structured education (mode not specified; presumably face-to-face).<br>**Setting:** Hospital-based (inpatient education during hospitalization for admitted patients; outpatient education during clinic visits for others).	
	Negarandeh et al. (2019)	35/33	45-50:(42.9/42.4), 50-60:(22.9/30.3), 60-70:(34.3/27.3)	Not reported	Class II-III The specific values were not reported	Only reported as proportion with other diseases, without specifying types(77.1/84.8)	**No./length/frequency of session:** Twice weekly (20 min/session) for the first month, then weekly (20 min/session) for the second month (adjustable based on patient needs).<br>**Format/delivery mode:** Telephone-based remote monitoring (tele-monitoring) with interactive education.<br>**Setting:** home	2 months/1 month
	Atienza et al. (2004)	164/174	(median [IQR]):69 (61–74) / 67 (58–74)	(median [IQR]) :36(30–53) / 40 (30–55)	Class I (11.0/10.0), Class II(39.0/40), Class III(40.0/40.0),Class IV(10.0/10.0)	DM(35/38), Hypertension(54/53),IHD(33/31), Valvular heart disease (24/29)	**No./length/frequency of session:** Comprehensive discharge planning + outpatient HF management, including patient education (disease knowledge, symptom recognition, self-monitoring, medication adherence, self-adjustment of diuretics), family caregiver training, and 24-hour telephone consultation support.<br>**Format/delivery mode:** Nurse-led individualized education (supplemented with brochures); outpatient follow-up (cardiologist-led); telephone consultation.<br>**Setting:** Hospital (pre-discharge education), outpatient HF clinic (follow-up)	509 days/12-month.
	Cleland et al. (2005)	168/85	67.0±13.0/68.0±10.0	25.0±8.0/24.0±8.0	ClassI(5.0/ 6.0), Class II (16.0/13.0), Class III(18.0/30.0),Class IV(61.0/51.0)	CAD (61/68), Hypertension (44/40), Chronic AF(47/39),Chronic lung disease (24/29) DM (35/35), Stroke (9/8), CVD (38/36)	**Number/Length/Frequency:** Twice daily (morning and evening).<br>**Format/Delivery Mode:** Patient self-measurement (electronic scale, automatic BP monitor, single-lead ECG wrist-band electrode); automatic data transmission via telephone line to central server; nurse telephone support (monthly proactive calls + patient-initiated contact).<br>**Setting:** Home.	120 days, 240 days, and 450 days/120 days, 240 days, and 450 days
(n=4)	Del Sindaco et al. (2007)	86/87	77.4±5.9/77.5±5.7	33.5±11/32.5±10	Class II (37.2/39.1), Class III(51.2/56.3) Class IV(11.6/4.6)	Hypertension (67.4/62.1), DM(32.6/31.0), COPD(35.9/33.3), previous MI(51.2/55.1)	**No./length/frequency of session:**<br>Hospital follow-up: Initiated within 14 days after discharge, followed by visits at 1 month, 3 months, and every 6 months (mean 4.9 times per patient);<br>Nurse telephone follow-up: Mean 8.5 times per patient (total 731 sessions, ~15 minutes per session)<br>**Format/delivery mode:** Multidisciplinary collaboration (cardiologists + nurse coordinators + general practitioners); combined hospital outpatient follow-up, home visits, and	2 years/Baseline to 2 years

								telephone follow-up<br>**Setting:** Hospital heart failure clinic + home	
		Tsuchihashi-Makaya et al. (2013)	79/82	76.9±10.9/75.8±12.1	47.4±16.6/47.4±15.7	Class I(10.1/17.1),Class II(84.8/76.8),Class III(5.1/6.1)	Hypertension (51.9/50.0), DM(25.3/22.0), Hyperuricemia (44.3/42.7), previous MI(21.5/18.3), stroke (17.7/14.6), COPD (10.1/4.9), AF (43.0/62.2)	**No./length/frequency of session:** First home visit within 14 days after discharge; home visits every 2 weeks for the first 2 months; monthly telephone follow-up thereafter until 6 months<br>**Format/delivery mode:** Nurse-led; home visits (face-to-face) + telephone follow-up<br>**Setting:** home	1 year / Baseline-2 months-6 months-12 months
		Angermann et al. (2012)	352/363	67.7±12.8/69.4±11.5	30.0±8.0/30.0±8.0	Class I (3.0/2.0), Class II(54.0/62), Class III(40.0/31.0), Class IV(3.0/5.0)	Hypertension (72.0/77.0), DM(36.0/36.0), COPD (18.0/21.0), CAD(55.0/61.0), AF (32.0/26.0), Renal dysfunction(42.0/41.0), Anemia(31.0/32.0)	**No./length/frequency of session:** Once weekly for the first month, then adjusted based on NYHA class (every 2 weeks for NYHA III-IV, once monthly for NYHA I-II); 10-15 minutes per session.<br>**Format/delivery mode:**Telephone-based, combined with face-to-face training during hospitalization; using standardized modules (e.g., START monitoring module, education module).<br>**Setting:** Hospital-led call center, patients' homes	6 months/6 months
		Blue et al. (2001)	84/81	74.4±8.6/75.6±7.9	Not reported	Class II (23.0/20.0), Class III(34.0/42.0),Class IV(43.0/38.0)	Hypertension (43.0/52.0), DM(18.0/19.0), previous MI(55.0/51.0), Chronic lung disease(27.0/22.0), AF (35.0/30.0), Angina(29.0/41.9), Valve disease(18.0/15.0)	**No./length/frequency of session:** Planned home visits (decreasing frequency) and telephone follow-up as needed for 1 year<br>**Format/delivery mode:** Individualized; home visits + telephone follow-up<br>**Setting:** home	12 months/12months
Patient education + Exercise therapy (Edu+MD)	(n=2)	Chen et al. (2018)	19/18	61±11/60±16	36±9/32±11	Not reported	ICM(31.6/16.7), RHD(0/5.6), DCM(68.4/83.3)	**No./length/frequency of session:** at least 3 sessions per week/30 minutes per session<br>**Format/delivery mode:** individualized; developed based on CPET and 6MWT results; telephone follow-up once every 2	3 months/3 months

								weeks<br>**Setting:** home	
		Mueller et al. (2007)	25/25	55.0±10	<40	Not reported	Not reported	**No./length/frequency of session:** 1-month intervention period; 2 hours of exercise daily (indoor cycling 5 times/week, 30 minutes per session; outdoor walking twice daily, 45 minutes per session)<br>**Format/delivery mode:** Individualized exercise prescription combined with educational sessions and dietary guidance<br>**Setting:** Residential rehabilitation centre	1 month/ 6.2±1.4 years
Health information tracking (HT)	(n=7)	Antonicelli et al. (2008)	28 / 29	77±8/79±6	35±6/37±7	ClassI(54/62),ClassII(43/31),Class IV (4/7)	Not reported	**No./length/frequency of session:** At least once per week.<br>**Format/delivery mode:** Telephone (transtelephonic).<br>**Setting:** home.	12 months / 12 months
		Giordano et al. (2008)	230 / 230	58±10/56±10	28±7/26±8	Class II(54/65), Class III–IV(46/35)	Previous MI(53/51), Chronic atrial fibrillation(15/17), Chronic lung disease(28/26), Hypertension(19/23), Diabetes(29/27)	**No./length/frequency of session:** Scheduled: weekly/fortnightly; Unscheduled: as needed<br>**Format/delivery mode:** Telephone with transtelephonic 1-lead ECG device (Card-Guard 2206).<br>**Setting:** Call Center supported by hospital nurses/cardiologists Usual care	1 year (Median 296±91 days)
		Olivari et al. (2018)	229 / 110	79.6±6.8/80.9±7.3	39.1±12.9 / 39.1±14.6	ClassII (48.0/48.2), Class III(47.2/46.4),Class IV(4.8/5.4)	Atrial fibrillation(34.9/48.2), Cerebrovascular diseases(9.2/6.4), COPD(19.7/19.1), Diabetes(38.9/26.4), Renal disease(29.7/31.8), Malignancy(9.6/12.7)	**No./length/frequency of session:** Daily on weekdays<br>**Format/delivery mode:**Wearable Wrist Clinic device + digital scale, telephone transmission<br>**Setting:** home, linked to a regional monitoring centre	12 months
		Villani et al.(2014)	40 / 40	71±4 / 73±5	31±6 / 32±8	At enrolment:3.08±0.57)/2.90±0.69.	Ischaemic heart disease(42.5/65), Diabetes(45/50), Hypercholesterolaemia(20/35)	**No./length/frequency of session:** Daily (vitals), monthly (psych assessment).<br>**Format/delivery mode:** Wireless PDA device with interactive software.<br>**Setting:** Patient's home, connected to monitoring centre.	12 months

				At 12 months: 2.08±0.38 /2.40±0.45			
Kotooka et al. (2018)	90/91	67.1±12.8/65.4±15.6	40.5±14.8/39.2±16.5	Class II (77.8/79.1), Class III(22.2/20.9)	History of IHD(31.1/29.7)	**No./length/frequency of session:** At least one measurement per day; nurse monitoring available 7 days/week, 9:00-17:00 daily.<br>**Format/delivery mode:** Electronic body-composition scale, blood pressure monitor, and wireless data-transmission device; patients performed self-measurements, and data were automatically uploaded to the central server.<br>**Setting:** home	Until the end of follow-up (mean follow-up 15 months, range 0–31 months)./ Baseline to study end (mean 15 months)
Dar et al. (2009)	91 / 91	70±12.8 / 72 ±10.4	39% of all assessed patients had EF ≥40% (Preserved LV systolic function: EG 39%, CG 40%)	Class II-IV at discharge (specific distribution not provided)	Previous myocardial infarction (44/53), Valve disease (5/5), Hypertension (60/63), Stroke (9/13), Diabetes (34/37), COPD (9/9), Chronic renal failure (69/69)	**No./length/frequency of session:** Daily (weekday mornings)<br>**Format/delivery mode:** Honeywell HomMed monitor connected to domestic phone line<br>**Setting:** home	6 months
Cleland et al. (2005)	168/85	67.0±13.0/68.0±10.0	25.0±8.0/24.0±8.0	Class I(5.0/ 6.0), Class II (16.0/13.0), Class III (18.0/30.0), Class IV	CAD(61/68), Hypertension(44/40), Chronic AF(47/39),Chronic lung disease (24/29) DM(35/35), Stroke (9/8), CVD (38/36)	**Number/Length/Frequency:** Twice daily (morning and evening).<br>**Format/Delivery Mode:** Patient self-measurement (electronic scale, automatic BP monitor, single-lead ECG wrist-band electrode); automatic data transmission via telephone line to central server; nurse telephone support (monthly proactive calls + patient-initiated contact).<br>**Setting:** Home.	120 days, 240 days ,and 450 days/120 days, 240 days ,and 450days

						(61.0/51.0)			
Patient education + Psychological interventions (Edu+PI)	(n=1)	Jiang et al. (2021)	49/56	69.1±10.5/68.8±13.1	Not reported	NYHA I-II(32.7/30.4); NYHA III-IV(67.3/69.6)	CHD(55.1/50.9), Hypertension (65.3/91.1), T2DM (59.2/60.7)	**No./length/frequency of session:** three 40-min~1 h biweekly home visits.<br>**Format/delivery mode:** individual; face to face<br>**Setting:** home	6weeks/Baseline-1.5-3-6 months
Patient education + Multidisciplinary team Management + Health information tracking (Edu+MD+HT)	(n=1)	Kalter-Leibovici et al. (2017)	682/678	70.8±11.6/70.7±11.0	Preserved(>50):(20.2/16.6), Reduced(<50): (79.8/83.4)	Class I (0.7/0.6), Class II(11.9/17.1), Class III(79.9/78.0), Class IV(7.5/4.3)	Hypertension (74.2/74.7), DM(52.2/50.0), COPD (18.0/15.5), Chronic lung disease(27.0/22.0), Chronic AF (23.4/26.9), Renal failure (56.5/56.8)	**No./length/frequency of session:**Remote nurse contacts (27.3±17.8 times in the first year; 18.8±13.7 times per year overall); follow-up at the cardiac center ≥once every 6 months<br>**Format/delivery mode:** Multidisciplinary team (nurses, cardiologists, dietitians, social workers); remote monitoring (Medic4All® device); telephone/video communication.<br>**Setting:** Community cardiac center, home.	2.7 years (range: 0-5.0 years)/2.7 years (range: 0-5.0 years)
Psychological interventions (PI)	(n=2)	Dekker et al. (2012)	20/21	68.0±10.0/64.0±12.0	40.8±17/38.1±16.1	Class II (29/10), Class III (71/76), Class IV (0/14)	COPD (57/25) CAD (62/62), history of MI (38/43), AF(38/52), DM(43/48), renal insufficiency (43/33), stroke (19/19)	**No./length/frequency of session:** one 30-min session and 5~10-min telephone booster session<br>**Format/delivery mode:** individual; face to face and telephone<br>**Setting:** hospital and home	1 week/Baseline-1 week-3 months
		Chew et al. (2021)	72/72	58.4±14.0/62.8±10.5	33.5±13.1/33.9±12.0	Class I (43.1/47.2), Class II (55.6/45.8), Class III (1.4/7.0)	MI (58.3/65.3), Hypertension (58.3/72.2), DM (38.9/61.1), AF(23.6/41.7),AF (23.6/41.7), CKD(19.4/29.2)	**No./length/frequency of session:** one 30-min face to face session and three 10-min reinforcement telephone follow-up sessions (week 3,6, and 9)<br>**Format/delivery mode:** individual; face to face and telephone<br>**Setting:** hospital and home	3months/Baseline- 3-6 months
Exercise therapy (Ex)	(n=5)	Corvera-Tindel et al. (2003)	42/37	63.8±10.1/61.3±11.1	29.1±8.5/24.7±8.8	ClassII(76.2/83.8), Class III-IV(21.4/16.2)	Arthritis(42.9/32.4),Hypertension(76.2/73.0), DM(40.5/29.7)	**No./length/frequency of session:** 5 days/week, once daily, progressive increase from 10 to 60 minutes<br>**Format/delivery mode:** Individual; pedometer for tracking, weekly nurse home visits (first 6 weeks) and biweekly visits (last 6 weeks)<br>**Setting:** home	3 month/3 month

		Dracup et al. (2007)	86/87	53.3±12.7/54.6±12.5	26.7±6.7/26.1±7.0	Class II(32.3/21.8),Class III(59.3/66.7), Class IV(8.1/11.5)	CAD(53.5/34.9), Hypertension(45.3/45.3), DM(31.4/19.8)	**No./length/frequency of session:** Aerobic exercise 4 times weekly; resistive training 3 days/week (on non-walking days)<br>**Format/delivery mode:** Home-based; self-administered with initial research nurse guidance; pedometer and daily log for compliance monitoring<br>**Setting:** home	12 months / Baseline-3 months-6 months-12 months
		Giannuzzi et al. (2003)	45/45	60±7/61±7	25/25	Class II (62/73), Class III (38/27)	Hypertension(66.0/60.0)	**No./length/frequency of session:** 3–5 sessions per week, 30 minutes per session (gradually increasing intensity and duration initially); Home-based training: Brisk walking for ≥30 minutes daily + intermittent gymnastics (30 minutes per session)<br>**Format/delivery mode:** Supervised group training + individual home-based training; combined with exercise diary documentation<br>**Setting:** Cardiac rehabilitation center (supervised training) + home (unsupervised training)	6months/Baseline-6 months
		Hambrecht et al. (1995)	12/10	50±12/52±8	26±9/27±10	Class II (50/60), Class III (50/40)	Not reported	**No./length/frequency of session:** Initial 3-week in-hospital training: 6 sessions per day, 10 minutes per session, intensity at 70% of peak oxygen consumption (VO_2peak);<br>Post-discharge home-based training: 2 sessions per day, total ≥40 minutes; at least 2 sessions of 60-minute group training per week; target heart rate corresponding to 70% of VO_2peak<br>**Format/delivery mode:** Individualized supervision (in-hospital) + home-based self-administered training (combined with ECG heart rate monitoring) + group training<br>**Setting:** Hospital (first 3 weeks) + home + group training venue	6months/Baseline-6 months
		Willenheimer et al. (2001)	17/20	64±5/64±8	35±12/38±10	2.1±0.7/2.4±0.7	IHD(85.0/78.0)	**No./length/frequency of session:** Gradually increased from 15 min twice/week to 45 min three times/week (total 16 weeks)<br>**Format/delivery mode:** Group-based, supervised by physiotherapist<br>**Setting:** hospital	4-month /6-month follow-up
Patient education + Multidisciplinary team management + Exercise therapy (Edu+Ex+MD)	(n=1)	Chen et al. (2017)	31/31	61.1±14.2/62.4±14.9	39.9/47.1	Class II (3.2/3.2), Class III (48.4/51.6), Class IV (48.4/45.2)	Hypertension (48.4/54.8), DM(19.4/25.8), previous MI(19.4/12.9), stroke (9.7/6.5), Renal dysfunction (12.9/6.5), AF	**No./length/frequency of session:** Home visit at 2 weeks post-discharge; telephone calls every 2 weeks; intensified education at 90 and 180 days; physical training 3 sessions/week (progressing from 20 to 40 min/session)<br>**Format/delivery mode:**Multidisciplinary team (cardiologists, nurses, dietitian, psychiatrist); individual; face-to-face (discharge education, home visits, clinic follow-up), telephone calls<br>**Setting:** Hospital, home, outpatient clinic	6 month/Baseline-3 month- 6 month

							(38.7/35.3), Anemia(29.0/41.9)		
Patient education + Psychological interventions + Health information tracking (Edu+PI+HT)	(n=1)	Jiang et al. (2021)	57/56	66.8±11.8/68.8±13.1	Not reported	ClassI-II(28.1/30.4;ClassIII-IV(71.9/69.6)	CHD(64.9/50.9), Hypertension (65.3/91.1), T2DM(52.6/60.7)	**No./length/frequency of session:** three 40-min~1 h biweekly home visits.<br>**Format/delivery mode:** individual; face to face and smartphone application<br>**Setting:** home	6weeks/Baseline-1.5-3-6 months

Note: No., number; EG, experimental group; CG, control group; LVEF, left ventricular ejection fraction; NYHA, New York Heart Association classification; COPD, chronic obstructive pulmonary disease; DM, diabetes mellitus; PVD, peripheral vascular disease; AF, atrial fibrillation; AMI, acute myocardial infarction; OSA, obstructive sleep apnea; MI, myocardial infarction; CAD, coronary artery disease; IHD, ischemic heart disease; RHD, rheumatic heart disease; DCM, dilated cardiomyopathy; ICM, ischemic cardiomyopathy; CPET, cardiopulmonary exercise test; 6MWT, 6-minute walk test; VO_2 peak, peak oxygen consumption; AT, anaerobic threshold; T2DM, type 2 diabetes mellitus; HFpEF, heart failure with preserved ejection fraction; HFmEF, heart failure with mid-range ejection fraction; HFrEF, heart failure with reduced ejection fraction; CHD, coronary heart disease; VHD, valvular heart disease; CKD, chronic kidney disease; ECG, electrocardiogram; BP, blood pressure; HR, heart rate; BNP, B-type natriuretic peptide; ICD, implantable cardioverter-defibrillator; WCD, wearable cardioverter-defibrillator; NMA, network meta-analysis; GDMT, guideline-directed medical therapy; QOL, quality of life; Ex+MD, exercise therapy + multidisciplinary team management; Edu+HT, patient education + health information tracking; Edu, patient education alone; Edu+MD, patient education + exercise therapy; HT, health information tracking alone; Edu+PI, patient education + psychological interventions; Edu+MD+HT, patient education + multidisciplinary team management + health information tracking; PI, psychological interventions alone; Ex, exercise therapy alone; Edu+Ex+MD, patient education + exercise therapy + multidisciplinary team management; Edu+PI+HT, patient education + psychological interventions + health information tracking.
